# High-throughput characterization of transcription factors that modulate UV damage formation and repair at single-nucleotide resolution

**DOI:** 10.1038/s41467-026-75115-4

**Published:** 2026-07-10

**Authors:** Hana I. Wasserman, Bo Chi, Kaitlynne A. Bohm, Mingrui Duan, Harshit Sahay, Alexias Safi, Gregory E. Crawford, Peng Mao, John J. Wyrick, Miles A. Pufall, Raluca Gordân

**Affiliations:** 1 Program in Computational Biology and Bioinformatics, Duke University, Durham, NC USA; 2 Center for Advanced Genomic Technologies, Duke University, Durham, NC USA; 3 Department of Computer Science, Duke University, Durham, NC USA; 4 School of Molecular Biosciences, Washington State University, Pullman, WA USA; 5 Department of Pathology, Stony Brook University Renaissance School of Medicine, Stony Brook, NY USA; 6 Department of Pediatrics, Duke University School of Medicine, Durham, NC USA; 7 Department of Molecular Genetics and Microbiology, Duke University Medical Center, Durham, NC USA; 8 Department of Biochemistry and Molecular Biology, Holden Comprehensive Cancer Center, Carver College of Medicine, University of Iowa, Iowa City, IA USA; 9 Department of Biostatistics & Bioinformatics, Duke University Medical Center, Durham, NC USA; 10 Department of Genomics and Computational Biology, University of Massachusetts Chan Medical School, Worcester, MA USA

**Keywords:** DNA adducts, Transcriptional regulatory elements, Computational models

## Abstract

Genomic studies revealed elevated DNA damage and mutation rates at transcription factor (TF) binding sites in UV-linked cancers. While TFs can promote UV-induced mutagenesis by altering both damage formation and repair, these mechanisms have not been systematically characterized across TFs at high resolution. Using genome-wide UV damage maps from skin fibroblasts, we develop a scalable statistical framework to analyze TF-mediated mutagenic mechanisms across hundreds of TFs. We identify numerous previously unreported TFs that significantly enhance or suppress UV damage formation within their binding sites. A systematic survey of TF-DNA complexes reveals that damage modulation often coincides with TF-induced DNA distortions that either protect against or promote photodimer formation. Additionally, we analyze repair efficiency in TF binding sites at high resolution, identifying TFs likely to compete with repair. Comparisons with skin cancer mutations distinguish mutation enrichment driven by increased damageability versus attenuated repair, revealing the highly contextual nature of TF-mediated mutagenesis.

## Abstract (web summary)

Transcription factors can promote UV-induced mutagenesis by altering both DNA damage formation and repair. Here, the authors systematically dissect these mechanisms across hundreds of human transcription factors, revealing widespread and highly context-dependent mutagenic effects.

## Introduction

The vast majority of the tens of thousands of DNA damages that occur daily in a human cell are repaired by conserved repair mechanisms^1^. Despite these repair pathways, some DNA damages are left unrepaired, resulting in downstream mutagenesis. DNA mutations are not uniformly distributed across the genome and are instead associated with structural genomic features and regulatory processes^2–4^. Genomic studies have shown elevated rates of mutations in active transcription factor (TF) binding sites in several tumor types^2–8^, with the trend being most prominent in melanomas, which are linked to ultraviolet (UV) light exposure^5–7,9,10^. Upon UV irradiation, adjacent pyrimidines (dipyrimidines) in DNA can dimerize, creating photoproduct lesions, such as cyclobutane pyrimidine dimers (CPD) and 6–4 pyrimidine-pyrimidones (6–4 PP), which in turn are precursors to C>T mutations if left unrepaired^11–13^.

Active TF binding sites are generally depleted for nucleotide excision repair (NER) activity, and this depletion correlates with somatic mutation enrichment in skin cancers^5,9,14–16^, leading to the hypothesis that DNA-bound TFs compete with NER proteins that must recognize UV lesions to initiate the repair process; in turn, slower repair then leads to increased mutagenesis. This model, however, requires that TFs bind strongly to DNA binding sites containing UV damage in order to occlude recognition by repair factors. Previously, Sivapragasam et al. demonstrated that CTCF can bind a putative binding site containing a site-specific CPD lesion in vitro and inhibit repair by a model repair enzyme^17^. Using high-throughput in vitro protein-DNA binding experiments, our group also demonstrated that TFs can indeed bind to UV-damaged sites and compete with UV-DDB, a major repair protein that recognizes UV lesions in human cells^18^. However, our data also showed that TF binding specificity is different for UV-damaged versus undamaged DNA, and most TFs bind more weakly to their binding sites after UV irradiation. Thus, although our prior work supports the theory that direct TF-NER competition can attenuate repair and promote mutagenesis of UV lesions, it also suggests that other mechanisms may be at play to explain the widespread mutation enrichment observed at TF binding sites in skin cancer.

Another critical component in understanding the mutagenic role of TFs in UV-exposed cells is characterizing how TF binding enhances UV damage formation. Early locus-specific studies by Pfeifer and colleagues demonstrated that DNA-bound TFs can locally modulate UV photoproduct formation and create hotspots of UV damage within regulatory elements, thus establishing the principle that TF-DNA interactions can influence UV lesion formation at nucleotide resolution^19,20^. More recently, genome-wide UV damage mapping methods in human cells showed that binding sites of specific TFs, like E26-transformation (ETS) factors, exhibit elevated CPD formation immediately after UV irradiation^16,17,21–23^. Structural analyses of ETS-bound DNA suggest this increase is due to the structural distortion of dipyrimidines upon protein binding, which increases the likelihood of photodimerization at specific positions within the binding site^22^. Consequently, mutation enrichment observed in melanoma at ETS binding sites has been largely attributed to increased UV damage formation from TF binding, resulting in UV lesion hyperaccumulation that is insufficiently repaired by NER^16,22,24^. However, beyond select TFs like ETS and CCCTC-binding factor (CTCF), the impact of TF binding on UV damage formation remains poorly characterized. We hypothesize that the effect of TF-binding on UV damage formation is likely widespread, given that many TFs significantly distort B-form DNA upon binding^25^. This hypothesis is consistent with the early observations of TF-dependent modulation of UV photoproduct formation^19,20^, but has yet to be systematically assessed across a broad range of TFs.

While collectively there is compelling evidence that TFs contribute to UV-induced mutagenesis by both increasing damageability and attenuating repair^26^, and despite the considerable number of TFs that show enrichment of UV-induced mutations in their binding sites^2–4,8,16^, there has yet to be a systematic analysis to identify the specific TFs and binding site positions that exhibit these mutagenic effects. We present such an analysis here, leveraging high-resolution maps of UV-induced lesions from CPD-seq and UVDE-seq assays of UV-irradiated human skin fibroblasts. We develop statistical models of UV damage formation and repair that rigorously control for DNA sequence bias. Applying these models to ~600 human TFs revealed widespread and varied effects of TF binding on CPD damage formation and repair. Additionally, we report a large-scale, single-nucleotide resolution assessment of 6–4 PP lesion formation at TF binding sites in human cells, showing dramatic enhancement from binding of specific TFs, significantly above the level of 6–4 PP formation expected based on DNA sequence alone. Structural analyses revealed that these effects on UV damage formation coincide with structural changes induced by TF-binding that predispose DNA to CPD or 6–4 PP photodimerization. The increased resolution and sensitivity of our models, compared to previous work^5,9,14,16,17,21–23^, allowed us to identify 197 and 289 TFs that are likely to modulate CPD and 6–4 PP formation, respectively, and 75 TFs that are likely to compete with NER for the repair of CPD lesions. Our study enables prioritization of TFs and binding site sequences for future experimentation to further demonstrate and explore TF-induced mutagenic mechanisms, and provides a scalable methodology that can be adapted for analyzing TF interactions with other forms of DNA damage.

## Results

### Statistical modelling of CPD formation identifies both enhancement and depletion of CPD formation in TF binding sites

The recently developed CPD-seq v2.0 assay maps CPD lesions genome-wide at single nucleotide resolution^27^, producing less background and requiring a lower UV irradiation dose than the original CPD-seq protocol^22^. Importantly, unlike alternative damage-mapping assays, such as HS-Damage-seq^21^, CPD-seq assays do not use anti-lesion antibodies, which can introduce biases in the data^28^. Here, we use CPD-seq v2.0 data in human skin fibroblast (CS1AN/CSB^WT^) cells to analyze the CPD profiles of human TF binding sites.

To perform an expansive characterization of TF binding effects on CPD formation, we leveraged genome-wide TF binding site calls published by Vierstra et al.^29^. Briefly, Vierstra and colleagues took >2000 publicly available human and mouse TF binding motif models and grouped them by motif similarity to create nonredundant motif clusters of proteins with highly similar binding specificity. Genome-wide binding sites were then called for each motif cluster, providing a set of comprehensive, putative binding sites for hundreds of human TFs. We intersected these sites with chromatin accessibility data in CS1AN/CSB^WT^ cells to curate active TF binding sites for our study (“Methods”). In total, we analyzed 225 of these TF motif clusters, which include the putative binding sites of 597 distinct human TF proteins (“Methods” and Supplementary Data 1), and we used CPD-seq data of cells processed immediately after UV irradiation to assess the effects of TF binding on CPD formation.

**Fig. 1:**
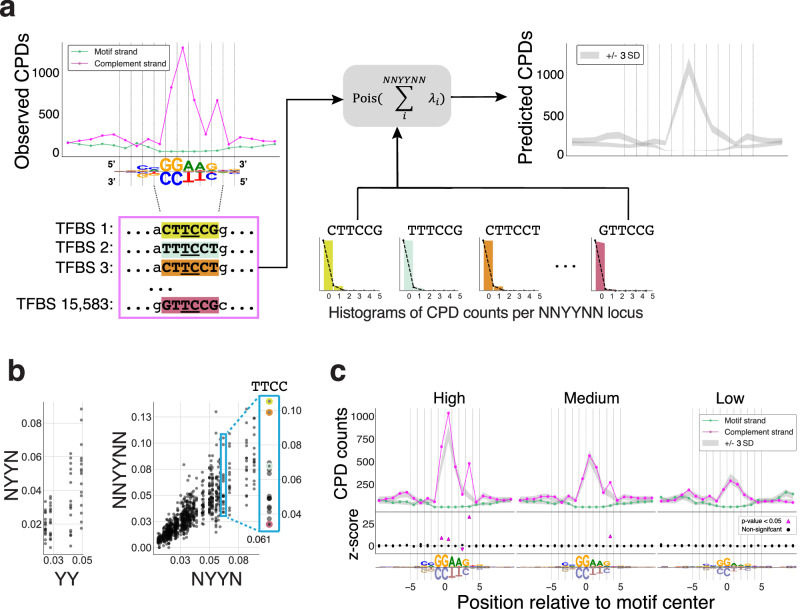
Assessing enrichment and depletion of CPD formation in TF binding sites. **a** Statistical modeling of CPD formation across active ETS binding sites (TF motif cluster: ETS/1) using CPD-seq v2.0 data from human skin fibroblasts after irradiation with 6 J/m^2^ UVC. Of the 31,153 ETS sites called, the top 50% highest scoring sites, including ties, were used for statistical analyses (*n* = 15,583, “Methods” and Supplementary Data 1). Green and pink lines in the observed CPD profile plot (left) represent raw CPD counts per position in the ETS GGAA-motif and motif-complement strands, respectively. CPD markers are positioned between nucleobase positions, indicating the photodimer location. Gray shaded region in the predicted CPD profile plot (right) denotes ±3 standard deviations of the expected CPD levels, according to the background Poisson model of CPD formation, conditioned on the cumulative hexanucleotide sequence content at each position. At the bottom are example histograms of CPD count per genomic locus grouped by hexanucleotide sequence from CPD-seq data, with dashed lines showing the Poisson fits. **b** Scatter plots illustrating the effect of tetranucleotide (left) and hexanucleotide (right) sequence context on CPD formation frequency, calculated from CPD-seq data from all chromatin-accessible intergenic regions (“Methods”). Cutout shows a magnified view of CPD formation frequency variation across hexanucleotides containing the same central TTCC tetranucleotide; colors mark the four hexanucleotides highlighted in (**a**). **c** Full analysis of CPD formation in all ETS binding sites (*n* = 31,153), stratified by binding site strength. Binding sites were divided into equal thirds by motif quality score (“Methods”), with higher scores indicating stronger ETS binding. As in **a**, the green and pink lines are the observed CPD counts aggregated per position for the motif (green) and the motif-complement (pink) DNA strands. Gray shaded regions are the predicted CPD levels ±3 standard deviations. Below are z-scores per position, colored by strand. Triangle markers show positions with significantly enriched or depleted CPD counts after Benjamini–Hochberg (BH) correction of one-sided Poisson test *p*-values (*p* < 0.05), with marker orientation indicating the direction of effect. Sequence logos show motif content of sites used in each binding strength tier. X-axis denotes position relative to the binding motif center. Source data are provided in the Source data file.

We illustrate our analysis framework using the ETS/1 motif cluster, which contains 15,583 active ETS binding sites. Consistent with previous studies^16,21–23^, aggregating CPD lesions across ETS binding sites reveals a pronounced CPD peak on the strand complementary to the ETS GGAA-binding motif (i.e., the TTCC strand; Fig. 1a, left), which is expected given its high dipyrimidine content. To determine to what extent this signal is induced by TF binding, as opposed to being explained by sequence alone, we developed a background model of CPD damage formation. The frequency of CPD formation at any specific dipyrimidine (YY; where Y = pyrimidine base) depends on its immediate sequence context, through a combination of structural and electronic factors^30–37^. Indeed, when we calculated the frequency of CPDs formed for each UV-damageable tetranucleotide sequence (NYYN; *n* = 64), we found that the identity of nucleobases immediately flanking the central dipyrimidine had a significant effect on the frequency of CPD formation (Kruskal–Wallis test *p* = 2.26e-95), consistent with literature^14,30,34–37^ (Supplementary Fig. 1a and Supplementary Data 2). We also found that the hexanucleotide sequence context of dipyrimidines continued to significantly influence damageability (Kruskal–Wallis test *p* = 2.84e-09), with as much as an 8.9-fold difference in CPD formation frequency between hexanucleotides containing the same central NYYN (Fig. 1b). Thus, we used hexanucleotide information to develop our background models of UV-induced damageability.

**Fig. 2:**
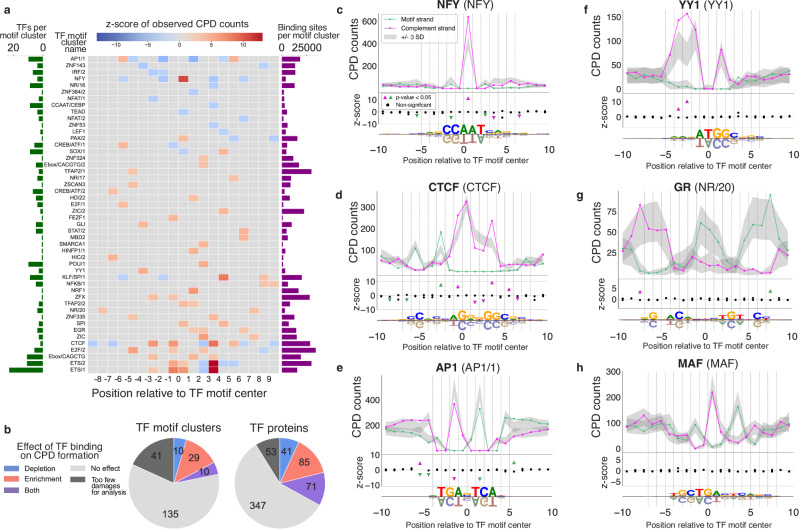
Modulation of CPD damage formation at active TF binding sites in human skin fibroblasts. **a** Heat map showing enrichment and/or depletion of CPD formation in TF binding sites, for 49 binding motif clusters with significant signals (one-sided Poisson test, BH-corrected *p* < 0.05). The z-score of the observed CPD signal was determined per position, for each strand of each TF cluster. At each position, the z-score with the largest magnitude (for either the motif strand or the motif-complement strand) was used as the cell value. Each row is a specific TF cluster. To focus on the highest-confidence results and correct for any additional bias not captured by our background model, we additionally spot-checked for type 1 error by performing the same analysis on CPD-seq v2.0 data from naked DNA and excluding any significant results that were also present at the same position in the naked DNA condition (Supplementary Fig. 5b). The bar plots on each side of the heatmap illustrate the number of distinct TFs mapped to each TF cluster (left) and the number of binding sites attributed to each TF cluster (right). **b** Pie charts summarizing the effect of TF binding on CPD formation across all 225 TF clusters and all distinct 597 TF proteins curated for analyses. TF binding sites for each TF cluster were defined as the sequence matching the binding motif ±5 base pairs in either direction. **c**–**h** Full CPD formation analysis for CTCF (*n* = 10,000), NFY (*n* = 4195), AP1 (*n* = 18,553), YY1 (*n* = 3650), GR (*n* = 3374), and MAF (*n* = 10,221) binding sites. Green and pink lines show observed CPD counts aggregated by position on the motif and motif-complement strands, respectively. Gray shading denotes predicted CPD levels ±3 standard deviations. Z-scores for each position are shown below, colored by strand. Triangles indicate positions with significant CPD enrichment or depletion (one-sided Poisson test, BH-corrected *p* < 0.05), with orientation indicating the direction of effect. The TF cluster used for the analysis of each TF is included in parentheses. Source data are provided in the Source data file.

To model the expected level of CPD formation at TF binding sites based on the DNA sequence alone, we developed an aggregate Poisson model of CPD formation conditioned on all dipyrimidine-centered hexanucleotides (NNYYNN; *n* = 1024) and trained on all chromatin accessible regions. We focused on these regions, as opposed to using the entire genome, to more accurately reflect the expected damageability of promoters and enhancers, where most of the TF binding events occur^38^. In addition, we excluded all genic regions to avoid biases in DNA damageability linked to transcriptional activities, such as a higher level of DNA single-strandedness in transcribed regions, which has been noted to modulate CPD formation^37^. Next, we used the hexamer-based background model to calculate the expected CPD formation profiles across ETS binding sites, based on sequence alone (Fig. 1a, right). For each position on both strands of the ETS binding motif, we then asked whether the observed CPD level significantly deviated from the predicted level based on our Poisson model (at corrected *p* < 0.05). Positions with significant CPD enrichment or depletion across the ETS binding motif, as well as the magnitude of the deviations at each position, calculated as z-scores, are shown in Fig. 1c. Our results confirm the CPD enrichment previously reported at ETS sites based on comparisons to naked DNA^22–24^. For example, we found CPD peaks at positions 3/4, −1/0, and 0/1, and quantified the magnitude of the CPD enrichment at these positions (z-scores of 31.99, 8.16, and 6.73, respectively, Fig. 1c, left). Interestingly, we also found significant CPD depletion at position 2/3 (z-score = −4.01), indicating that ETS binding actually suppresses UV damage formation at this position. This depletion, which has not been previously reported in the literature, is consistent with our subsequent analysis of ETS-bound DNA structures. Alongside ETS, CTCF has also been studied extensively, compared to naked DNA^17,21^. Our results closely aligned with the trends presented previously by Sivapragasam et al.^17^, with significant damage enhancement at positions −3/−2, −1/0, 3/4, and 5/6 and depletion at positions 1/2 and 2/3 (Fig. 2d and Supplementary Fig. 4a).

Before extending our analysis framework to all TFs with available binding site predictions, we performed two additional analyses to gain further confidence that the significant signal identified by our models is attributable to TF binding. First, we stratified ETS and CTCF binding sites by their binding strength (high, medium, and low). Effects due to TF binding are expected to become weaker as the TF binding strength decreases; indeed, we observed this trend for both ETS and CTCF binding sites (Fig. 1c and Supplementary Fig. 4a). Second, we applied our analysis to CPD-seq v2.0 data generated for naked DNA (“Methods”), and filtered out any positions of significant CPD enrichment or depletion that were also observed in naked DNA (Supplementary Figs. 4–6), ensuring that the effects we identified are induced by factors present in the cell.

### High-throughput survey reveals widespread effects of TF binding on CPD formation

We applied our CPD formation analysis to 597 human TF proteins grouped into 225 clusters by motif specificity^29^ (Supplementary Data 1). To our knowledge, 537 of these TFs have not been previously assessed for their ability to modulate CPD formation. We identified 49 motif clusters, encompassing 197 TF proteins (33% of TFs analyzed), that exhibited significantly enhanced and/or depleted CPD formation within their binding site (Fig. 2a, b), where we set the width of the binding site to include the sequence matching the binding motif plus 5 base pairs flanking in either direction. Among the TFs that have significant effects, over one-third show both enrichment and depletion, depending on the binding site position and the strand. This finding underscores the importance of evaluating CPD formation at a single-nucleotide level, rather than aggregating signals across entire binding sites.

Second only to ETS, the largest CPD enrichment signal was observed at a TT dipyrimidine in the binding motif of nuclear factor Y (NFY), opposite to the AA in NFY’s CCAAT binding core motif (z-score = 10.77, Fig. 2c). This prominent effect has been noticed previously in comparisons to naked DNA analyses^16,20–23^, but a complete statistical analysis of CPD formation across the entirety of the NFY binding site has not been reported until now. Interestingly, this enrichment peak in the NFY CPD profile is flanked by positions with significant CPD depletion (z-scores = −4.43 and −4.35). The strongest depletion of CPD formation (z-score = −6.18) was observed at the binding sites of FOS/JUN factors (TF motif cluster AP1/1; Fig. 2e). This depletion has been mentioned in the literature, but its exact position was not consistent across previous studies^16,17,20,21^. Our analysis showed a clear and significant symmetrical suppression of CPD formation, specifically at the conserved TCs at positions −2/−1 and 1/2 in the AP1 binding sites, consistent with the palindromicity of the FOS/JUN binding motif. Thus, for TFs that have been previously reported to potentially modulate CPD damage formation, our analyses provide quantification of the TF-induced effects, at single-nucleotide resolution, and allow us to rigorously assess the statistical significance of the observed trends.

A critical advantage of our statistical framework is that it enables assessment of CPD enrichment and depletion even at positions with less conserved dipyrimidine content, which were not considered in previous comparisons of cellular versus naked DNA, as those relied on sufficient CPD counts to assess differences^16,23^. For example, we found CPD enrichment for TF YY1 at positions −4/−3 and −3/−2, directly 5′ to its CCAT-binding motif, which lacks a strong consensus dipyrimidine sequence (Fig. 2f). In addition, even for the well-studied ETS transcription factor family, we found that the largest CPD enrichment is at position 3/4, which is not a conserved dipyrimidine in the ETS consensus motif (Fig. 1a).

To our knowledge, 85% (174/197) of the human TFs we identified as having the potential to significantly modulate CPD formation have not been previously reported^16,23^. Most of these TFs have subtler effects and lower overall CPD signal in their aggregated binding sites, requiring more sensitive analyses to detect significant signals, compared to TFs like ETS. For example, the human glucocorticoid receptor (GR) has only 3374 putative active binding sites in its motif cluster (NR/20), as opposed to the 15,583 and 10,000 sites in the ETS/1 and CTCF clusters, respectively. Despite having relatively few putative binding sites, we are still able to detect symmetrical CPD formation enrichment (z-scores = 3.67 and 3.43) at positions −8/−7 and 7/8 in GR’s palindromic binding motif (Fig. 2g).

Not all TFs analyzed show significant effects on CPD damage formation. In fact, approximately 57% of TFs included in our analysis do not have any binding site positions with significant CPD enrichment or depletion (e.g., MAF, Fig. 2h), which is not unexpected given that very specific DNA distortions are required to induce CPD formation^30,32,33^. To complement our analysis, we also evaluated CPD formation in TF binding sites using an orthogonal, simulation-based approach where we shuffled all CPD damages from open chromatin regions by their tetranucleotide sequence context to create a null distribution of expected CPD formation (“Methods”). Significant signals identified using the simulation approach were consistent with our analytical Poisson method (Supplementary Fig. 7). In Supplementary Data 3 we provide the complete catalog of CPD formation profiles for all 597 TFs.

### Linking CPD formation in TF binding sites to skin cancer mutation profiles

We next asked if we could connect TF-induced CPD formation to somatic mutation enrichment curated from skin tissue samples of 183 skin cancer donors^39^ (“Methods”). As the primary mutagenic outcome of UV photodimers^11–13^, we focused our analysis on C>T base substitutions in TF binding sites, across the same set of 597 TFs described above. While other mutations can also occur from UV-induced damages^40,41^, C>T mutations from CPD lesions are the most prevalent^11–13^. For each TF cluster, we computed a “projected” C>T mutation profile by taking the observed CPD counts and assuming that all CPDs are converted into mutations (i.e., C>T mutations for all cytosines within CPD lesions, “Methods” and Supplementary Fig. 8).

**Fig. 3:**
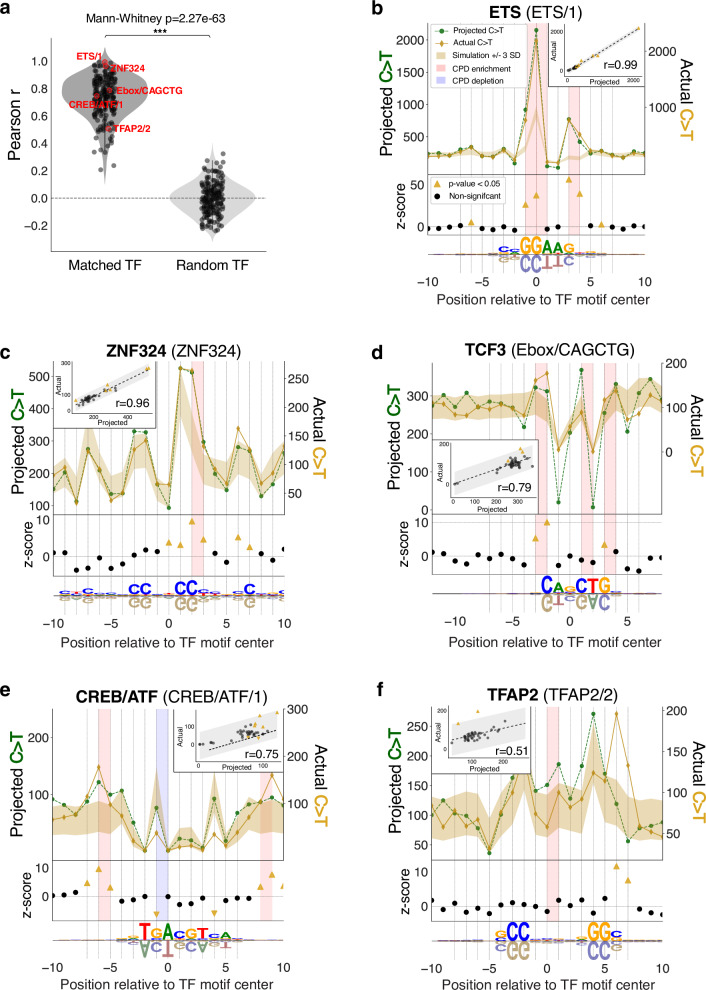
Comparative analysis of skin cancer mutations versus mutations projected from CPD formation. **a** (Left) Distribution of Pearson correlation coefficients comparing the actual skin cancer C>T mutation profile vs. the C>T mutation profile projected from initial CPD formation alone, in the binding sites of 225 TF clusters. Each marker represents the correlation for an individual TF cluster, where the Pearson correlation was computed across each position in the given TF’s binding site window between actual skin cancer C>T mutation counts and projected C>T mutation counts. Example TF clusters are annotated for reference. (Right) Distribution of Pearson correlation coefficients when the correlation is computed between the actual skin cancer C>T mutation profile of a TF cluster versus the projected C>T mutation profile of randomly-selected non-matching clusters (Supplementary Fig. 8). One-sided Mann–Whitney *U* test *p*-value is shown. Source data are provided in the Source data file. **b** Comparative analysis between the actual and projected C>T mutation profiles in ETS binding sites (*n* = 15,583). The dark green line shows the mutation counts projected from CPD counts (right y-axis), and the gold line represents actual mutation counts from skin cancer samples (left y-axis). Mutations were aggregated across both DNA strands for a total mutation count per TF binding site position. The shaded gold region denotes the ±3 standard deviations of the mean from 10,000 bootstrapped simulations of C>T mutation counts based on a background mutation model (“Methods”). Z-scores of the observed mutation counts, according to the same background model, are shown for each position. Gold triangle markers denote positions with significantly modulated C>T mutation levels (one-sided bootstrap test, BH-corrected *p* < 0.05), pointing in the direction of effect. Positions with vertical red shading indicate CPD formation enrichment. The inset shows a direct comparison between the actual C>T mutation counts vs. the projected C>T mutation counts; each point is a position in the ETS binding site. The gray shaded region is ±3 standard deviations from the ordinary least squares fit of the data (dashed line, “Methods”). **c**–**f** Similar to (**b**), but for TFs ZNF324 (*n* = 11,678), TCF3 (TF cluster Ebox/CAGCTG, *n* = 19,222), CREB/ATF (*n* = 5613), and TFAP2 (*n* = 8438) binding sites. Positions with vertical blue shading indicate CPD formation depletion. Source data are provided in the Source data file.

The actual and projected C>T mutation profiles showed overall high concordance across the 225 TF clusters analyzed (Fig. 3a), with a median Pearson’s correlation of 0.75. This correlation was substantially and significantly higher than a randomized comparison between actual mutations and TF-shuffled predicted mutations (Mann–Whitney *p* = 2.27e-63, median Pearson *r* ≈ 0, Supplementary Fig. 8) and is consistent with CPD formation being a primary driver of UV-linked mutagenesis^11–13^.

We used this comparative analysis to pinpoint mutation hotspots in TF binding sites that correspond to CPD formation enrichment identified by our prior analysis, directly implicating the mutagenic role of TF binding. To evaluate the statistical significance of mutation enrichment for each TF cluster, we simulated the expected C>T mutation level at each binding motif position, conditioned on the trinucleotide context of potential C>T substitutions (“Methods” and Supplementary Data 4). We again used ETS as our model TF to illustrate our analysis approach, as shown in Fig. 3b: the projected mutation profile for ETS exhibits strong concordance with the actual mutation profile (Pearson *r* = 0.993, inset). Prominent C>T mutation enrichment is observed at positions −1, 0, 3, and 4, which align with the positions of pronounced CPD formation enrichment (shaded red). These results are consistent with prior studies indicating that CPDs are the predominant mutagenic mechanism in ETS binding sites^16,22–24^.

We further identified mutation enrichment peaks that have not been previously attributed to TF-induced CPD formation. For example, both zinc finger protein 324 (ZNF324) and enhancer-box CAGCTG (EBOX/CAGCTG) factors have enriched mutations in their binding sites that coincide with CPD formation hotspots (Fig. 3c, d). Conversely, we observed mutation depletion in positions with suppressed CPD formation (shaded blue), as seen with activating transcription factor (ATF) and cAMP response element binding protein (CREB) binding sites, where both stimulatory and inhibitory effects on CPD formation are consistent with position-specific mutation enrichment and depletion, respectively (Fig. 3e).

Mutation enrichment that seemingly lacks precursory TF-induced CPD formation enrichment as a mutagenic driver was also identified for specific TFs. While the strongest mutation enrichment in ETS, ZNF324, and CREB/ATF binding sites directly aligned with CPD formation hotspots, there is also subtler, but still significant, mutation enrichment in positions flanking these peaks that do not have direct corresponding CPD formation enrichment (Fig. 3b, c, e). These discrepancies are especially visible for TFs with lower correlation between their actual and projected mutation profiles, such as transcription factor family activator protein 2 (TFAP2) (Pearson *r* = 0.507; Fig. 3f). This low correlation for TFAP2 is in part due to prominent mutation peaks at positions 6 and 7, where concordant CPD formation enrichment is absent. Interestingly, while there is CPD formation enrichment elsewhere in TFAP2 binding sites (position 0/1), it does not align with any mutation enrichment directly (Fig. 3f). These instances of mutation enrichment that are not explained by initial CPD formation suggest that alternative mechanisms are likely driving mutagenesis in these binding sites.

### High-resolution simulation identifies positions of reduced CPD repair in TF binding sites

Previous studies of NER of UV-induced damages have noted a general depletion of NER around aggregated TF binding regions after UV irradiation, indicating that TFs are somehow attenuating repair. One mechanistic explanation of this attenuation is that TFs bind strongly to their putative binding sites even when these sites contain UV lesions, thus outcompeting lesion-recognition repair factors that would otherwise initiate NER. With the precision of the CPD-seq v2.0 assay, we saw an opportunity to identify position-specific repair attenuation effects attributable to direct TF binding. Most prior analyses of DNA repair in relation to TF binding use data from XR-seq experiments^5,7,9,14,17,21,22^, providing a snapshot of repair activity in TF binding regions over time. While XR-seq offers powerful insight into NER trends generally, it has lower resolution than methods like CPD-seq, making precise mapping between repair and UV lesions difficult. This becomes especially limiting in many TF binding motifs where there are multiple damageable positions in close proximity. A key advantage of CPD-seq v2.0^27^ is that it produces less background signal and thus requires a reduced UV irradiation dose than the original 2018 CPD-seq method^22^. This lower UV irradiation allows for cell viability after treatment, enabling CPD-seq data to be collected from live cells at later timepoints following initial UV exposure. CPD-seq v2.0 thus allows characterization of the UV damage landscape given varying repair time, while maintaining the resolution necessary to identify specific binding site positions with reduced repair.

**Fig. 4:**
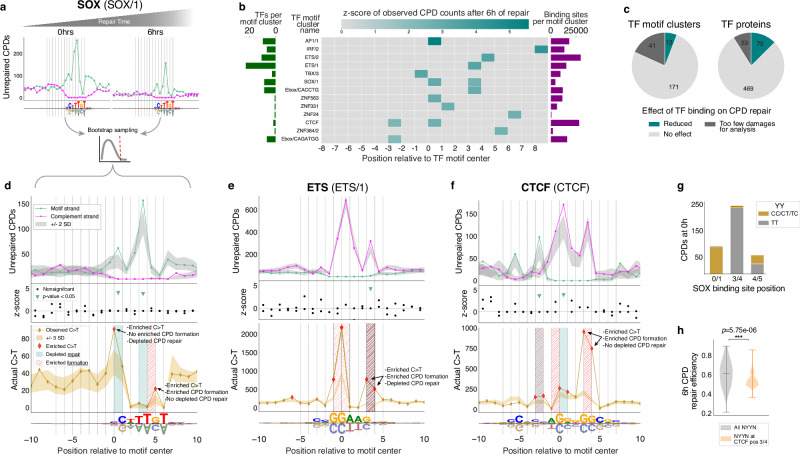
Analysis of CPD repair efficiency in TF binding sites using CPD-seq timecourse data. **a** Unrepaired CPD counts in SOX binding sites (*n* = 4787), from CPD-seq v2.0 experiments of cells irradiated with 6 J/m^2^ UVC and either 0 h or 6 h of repair. Repair efficiency was assessed by bootstrap simulation of 0 and 6 h timepoints, conditioned on tetranucleotide sequence content and CPD formation burden at each binding site position (Supplementary Fig. 14 and Supplementary Methods). **b** Heatmap summarizing the 13 TF clusters with significant repair depletion in their binding sites (one-sided bootstrap test *p* < 0.05, BH-corrected). Cell values represent the largest-magnitude z-score of unrepaired CPDs at 6 h from either DNA strand, with higher z-scores indicating elevated levels of unrepaired CPDs, signifying reduced repair. **c** Pie charts summarizing TF binding effects on CPD repair across 225 TF clusters and 597 TF proteins. **d** Full analysis of CPD repair in SOX binding sites (*n* = 4787) at 6 h. (Top) Observed unrepaired CPD counts aggregated by position on the motif (green) and motif-complement (pink) strand. The gray shaded region denotes ±2 standard deviations of the mean from 10,000 bootstrap simulations under the background repair model. (Middle) Scatter plot of z-scores per position, colored by strand. Triangle markers denote significant repair depletion. (Bottom) C>T mutation analysis for SOX binding sites. Mutations were aggregated across both DNA strands; the gold region denotes the expected C>T mutation counts (mean ±3 standard deviations) under the background mutation model. Red diamonds indicate significant C>T enrichment (BH-corrected one-sided bootstrap test *p* < 0.05), teal shading represents repair depletion, and red crosshatching represents CPD formation enrichment. **e**, **f** Repair and C>T mutation analyses of ETS (*n* = 15,583) and CTCF (*n* = 10,000) bindings sites, as in (**d**). **g** Bar plots of C>T mutagenic versus non-C>T mutagenic (i.e., TT) dipyrimidine content of CPDs formed at positions 0/1, 3/4, and 4/5 on the motif strand of SOX binding sites. **h** Violin plots showing the fraction of CPDs repaired after 6 h across all NYYN sequences (gray, *n* = 64) and NYYN sequences at position 3/4 on the motif-complement strand of CTCF binding sites (orange, *n* = 428). Center lines are medians, and whiskers indicate minimum and maximum values. One-sided Mann–Whitney *U* test *p*-value is shown. Source data are provided in the Source data file.

We leveraged timecourse CPD-seq v2.0 datasets from human skin fibroblasts collected 6 h after 6 J/m^2^ UVC irradiation to quantify CPDs over time and infer repair activity in TF binding sites (Fig. 4a). Assessing CPD repair becomes increasingly challenging as damage counts decrease with time, requiring a conservative statistical approach to distinguish true effects from noise. Accordingly, at each TF binding site position, we simulated the expected CPD levels after repair by sampling CPD damages from all open chromatin regions at the given time point (“Methods”). Because we found that CPD repair efficiency was significantly influenced by sequence context (Supplementary Fig. 9), the simulation was conditioned on both tetranucleotide sequence composition and initial CPD burden at each binding site position. Duan et al. also published a CPD-seq v2.0 dataset collected from irradiated cells given 24 h of repair time; however, we found that at this timepoint, some tetranucleotide sequences did not have sufficient unrepaired CPD signal to perform rigorous statistical modeling. For this reason, we focused our analysis in TF binding sites to the 6 h repair timepoint.

The heatmap in Fig. 4b summarizes our results across all 597 TFs in our study. Each cell value is the z-score of positions that have significant levels of unrepaired CPDs 6 h after initial irradiation, indicating repair depletion. We identified 13 TF clusters, encompassing 75 TF proteins, that have at least one TF binding site position with significantly reduced repair that is attributable to TF binding (Fig. 4c). Although fewer TF clusters exhibited effects from TF binding, compared to our CPD formation analysis, the number of TFs with detectable repair attenuation (~13%) is notable. Given the conservative nature of our approach, it is plausible that there is additional repair attenuation outside of our limit of detection. We also used our simulation framework to investigate whether TF binding may potentially enhance CPD repair efficiency in TF binding sites, and we identified 5 TF clusters, each containing one binding site position with significantly enhanced repair (Supplementary Fig. 10a). The complete catalog of effects across all 597 TFs at 6 h of repair are collated in Supplementary Data 3.

SRY-box (SOX/1) TFs exhibited some of the strongest effects on CPD repair, specifically at positions 0/1 (z-score = 4.00) and 3/4 (z-score = 3.78) in their binding sites (Fig. 4d, top). Upon examination of the mutation analysis we conducted previously, we see clearly that the reduced repair at position 0/1 coincides with C>T mutation enrichment at position 0, which was not explained by enhanced initial CPD formation alone, indicating that repair attenuation is the driving mutagenic mechanism at this position (Fig. 4d, bottom). We found no C>T mutation trend directly associated with the attenuative effect at position 3/4 in the SOX motif, but this is expected since it is at a highly conserved TT dipyrimidine, which is not C>T mutagenic (Fig. 4g).

Interestingly, the large CPD formation enhancement at position 3/4 in ETS binding sites also had significantly reduced repair (z-score = 4.24, Fig. 4e), suggesting that a combination of TF-induced CPD formation followed by TF-inhibited repair are collectively driving the skin cancer mutation enrichment at ETS positions 3 and 4 in skin cancer. An intriguing feature of these poorly repaired positions (e.g., SOX/1 position 0/1 and ETS/1 position 3/4) is that they are highly degenerate in the TF binding motifs, indicating the lack of a strong preference for any specific bases. It is possible that, because these positions are less critical to binding specificity, UV lesions at these positions can be better tolerated, allowing for the successful TF binding necessary to attenuate repair. Similar trends of repair attenuation signals corresponding to mutation peaks were also seen in the binding sites of other TFs, like T-box transcription factor 3 (TBX3) and Ebox/CACCTG factors (Supplementary Fig. 10b, c). Some of the significant repair attenuation signals did not directly coincide with a position of C>T mutation enrichment; however, these cases almost all occurred at positions with low starting levels of CPDs (average CPDs at 0 h = 78). Even with repair attenuation, it is possible that the amount of initial CPDs at these positions is insufficient for significant mutation enrichment to be observed.

We also found TF binding site positions where high enrichment of C>T mutations is explained by the intrinsically inefficient repair of dipyrimidines in certain sequence contexts. Like ETS, CTCF binding sites exhibit C>T mutation enrichment that aligns to both elevated CPD formation and depleted CPD repair (positions −3, −2, 0, and 1), largely consistent with literature^9,16,17,21^ (Fig. 4f, bottom plot). However, this mutation enrichment is overshadowed by the large C>T mutation peak at positions 3 and 4 (C>T z-scores = 55.68 and 47.86). Although position 3/4 does have significant CPD formation enrichment (Fig. 2d), the corresponding C>T mutation profile far exceeds what is expected based on this initial CPD formation alone (ordinary least squares regression residual *p* < 0.0001, Supplementary Fig. 11a, plot inset). We initially hypothesized that the discordance of this mutation peak was also due to a combination of CTCF-induced CPD formation and repair attenuation, in a manner similar to the rest of the CTCF binding site. Surprisingly, though, we saw no significant repair reduction attributable to CTCF binding at positions 3 and 4 at 6 h of repair (Fig. 4f, top plot). Closer examination revealed that CPDs at this position are primarily occurring at a conserved CC in a G-C sequence context (Supplementary Fig. 11b). When analyzing CPD repair efficiency by tetranucleotide sequence context, we noted that GCCC has the third-lowest CPD repair efficiency out of all 64 NYYNs at 24 h of repair (Supplementary Figs. 9, 11c, and Supplementary Data 2). Furthermore, across all tetranucleotides at this position, the intrinsic repair efficiency is significantly lower when compared to general NYYN tetranucleotides (Fig. 4h). Therefore, our analyses suggest that the poor lesion recognition by repair factors at position 3/4, in conjunction with the sizable damage burden from initial CPD formation enrichment, is driving the outsized mutation peak. This finding underscores the importance of incorporating sequence composition into analyses of DNA repair to distinguish between sequence-driven and TF-driven effects on repair.

### 6–4 PP formation is significantly enhanced by transcription factor binding

**Fig. 5:**
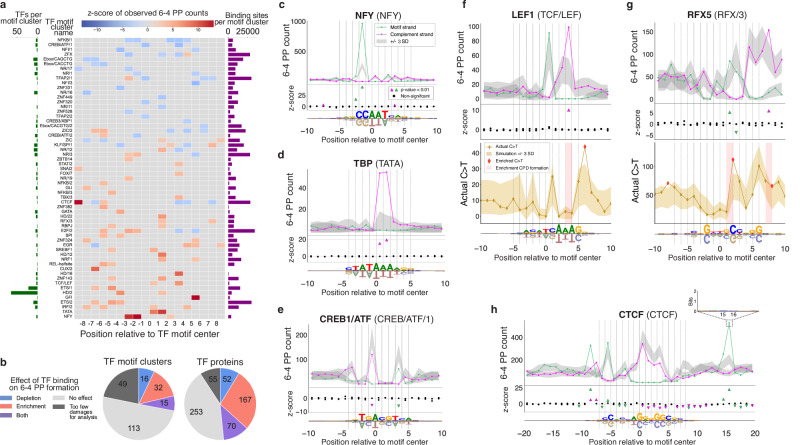
Modulation of 6–4 PP damage formation at active TF binding sites in human skin fibroblasts. **a** Heat map showing enrichment and/or depletion of 6–4 PP formation in TF binding sites, for 63 binding motif clusters with significant signals (one-sided Poisson test, BH-corrected *p* < 0.01). The z-score of the observed 6–4 PP signal was determined per position, for each strand of each TF cluster. At each position, the z-score with the largest magnitude (for either the motif strand or the motif-complement strand) was used as the cell value. Each row is a specific TF cluster. To reduce potential type I errors, positions showing significant effects were excluded if the same effect was also observed in an equivalent analysis of 6–4 PP formation in naked DNA (Supplementary Fig. 5b). The bar plots on each side of the heatmap illustrate the number of distinct TFs mapped to each TF cluster (left) and the number of binding sites attributed to each TF cluster (right). **b** Pie charts summarizing TF binding effects on 6–4 PP formation across 225 TF clusters and 597 TF proteins. **c**–**g** Full 6–4 PP formation analyses for NFY (*n* = 4195), TBP (*n* = 487), CREB1/ATF (*n* = 5613), LEF1 (*n* = 1858), RFX5 (*n* = 7908), and CTCF (*n* = 10,000) binding sites. Green and pink lines show observed 6–4 PP counts aggregated by position on the motif and motif-complement strands, respectively. Gray shading denotes predicted CPD levels ±3 standard deviations. Z-scores for each position are shown below, colored by strand. Triangles indicate positions with significant 6–4 PP enrichment or depletion (one-sided Poisson test, BH-corrected *p* < 0.01), with orientation indicating the direction of effect. The TF cluster used for the analysis of each TF is included in parentheses. Position weight matrix showing the NNYYNN sequence composition at the 6–4 PP hotspot at position 15/16 for CTCF is included in (**h**). Source data are provided in the Source data file.

Another driver of skin cancer mutation enrichment in TF binding sites could be non-CPD UV lesions. After CPDs, 6–4 PPs are the second most common UV lesion type, comprising 10–20% of UV damage, and are also mutagenic, preferentially forming between TC dipyrimidines^13,37,41,42^. However, their formation in relation to TF binding in human cells is poorly characterized. This is in part because, in addition to occurring less frequently^37,40^, isolating the 6–4 PP signal without using anti-lesion antibodies requires a CPD-specific repair step, making it more challenging to detect these lesions compared to CPDs. Recently, Bohm et al. developed UVDE-seq to map 6–4 PPs in yeast, similarly to CPD-seq^43,44^. Briefly, UVDE-seq first incubates UV-irradiated DNA with CPD photolyase to remove CPD lesions prior to treatment with ultraviolet damage endonuclease (UVDE), which cleaves a wide range of UV damages, allowing for mapping of non-CPD photolesions like 6–4 PPs at single-nucleotide resolution. Using UVDE-seq data from human skin fibroblasts irradiated with 500 J/m^2^, we adapted our analysis of CPD formation to assess the role of TF binding on 6–4 PP formation across our cohort of 597 TFs (Fig. 5a), of which only 19 have been previously examined for their impact on 6–4 PP formation across the human genome^21^. Overall, we identified 63 TF clusters, encompassing 289 human TFs that significantly modulated 6–4 PP formation (Fig. 5b). As observed with CPD formation, the widespread effects of TF binding on 6–4 PP formation are highly TF- and position-specific.

Our analysis revealed striking 6–4 PP formation enrichment in the active binding sites of several TFs, the most prominent being the conserved CC at position −2/−1 in the NFY motif (z-score = 28.52), with a lesser peak at −3/−2 (Fig. 5c). Although increased 6–4 PP formation in NFY binding sites was noted previously by Hu et al.^21^, this effect was reported to be located on the motif complement strand and was not quantified at the time. Interestingly, this CC 6–4 PP enrichment is adjacent to, but on the strand opposite of the TT CPD peak we noted in our CPD formation analysis. Bohm et al. saw similar enrichment in the binding sites of yeast NFY orthologs, Hap3 and Hap5, which also recognize a CCAAT-binding motif^44^. TATA-box binding protein (TBP), which had no effect on CPD formation, showed the second largest enrichment of 6–4 PP formation across all TFs analyzed, at positions 0/1 and 1/2 in the T-tract of the TBP binding motif (Fig. 5d). This finding is consistent with 6–4 PP formation trends seen in UVDE-seq data from the binding sites of yeast TBP^44^ and lower resolution studies that reported TBP increasing 6–4 PP formation levels generally^21,45,46^.

While enrichment was the predominant effect, some TFs, like CREB/ATF proteins (Fig. 5e), significantly protect their binding sites from 6–4 PP formation (z-score = −5.74) in a manner consistent with significantly depleted C>T mutation enrichment seen previously in Fig. 3e. As with CPD formation, a portion of TFs (12%) exhibited both enrichment and depletion of 6–4 PPs within their binding sites. A considerable amount of the 6–4 PP formation enrichment we identified was at TT dipyrimidines in TF binding motifs with T-tracts, such as TBP and LEF1 (Fig. 5d, f). While these TT 6–4 PPs are not precursors to C>T mutations emblematic of UV mutation signatures, we did identify select cases of 6–4 PP formation enrichment that directly corresponded to significant C>T mutation peaks, such as regulatory factor X5 (RFX5, Fig. 5g). To our surprise, the TFs with the largest stimulatory effect on 6–4 PP formation, like NFY, did not. However, these prominent 6–4 PP hotspots are sometimes accompanied by C>T mutation enrichment in neighboring positions (e.g. LEF1, Fig. 5f). Intriguingly, for TFs that otherwise showed no C>T mutation enrichment in their binding site (e.g. NFY, Supplementary Fig. 12), when we expanded our mutation analysis to all base pair substitution types (“Methods”), we observed highly significant enrichment (*p* < 0.001) of mutations broadly in the flanking regions, approximately ±17 base pairs from the motif center. In both cases, this mutation enrichment is also not explained by increased CPD formation or inhibited CPD repair.

We also noted instances of significantly enhanced 6–4 PP formation in the flanking DNA outside TF binding sites, as exemplified by CTCF (Fig. 5h). In contrast to the considerable inhibition of 6–4 PPs within the immediate CTCF binding site, there is a large 6–4 PP peak at position 15/16 (z-score = 24.03) on the motif strand, which is 8 base pairs past the 3′-end of the binding motif. This peak is well outside the binding core and is not a discernable binding motif for any other TF to our knowledge (Fig. 5h, inset). However, CTCF is known to interact with the clamp protein complex, cohesin, to promote DNA loop extrusion, 5–10 base pairs downstream of the 3′ end of the CTCF motif, approximately at this location^47–51^.

### Structural distortion of TF binding dictates both CPD and 6–4 PP formation

**Fig. 6:**
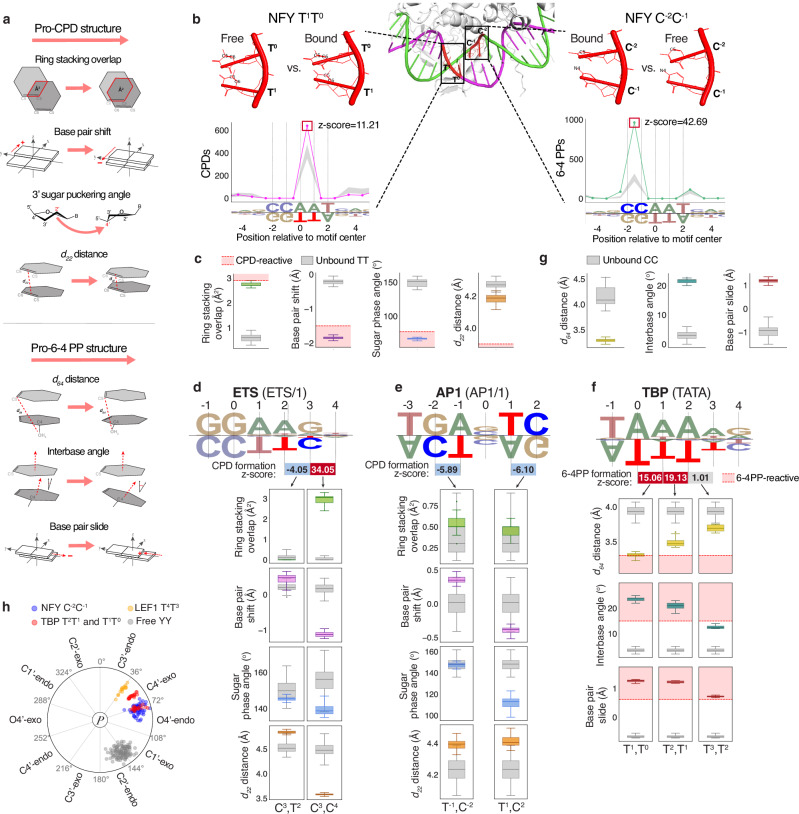
TF binding induces structural distortions favorable to UV dimerization. **a** Structural parameters that predispose dipyrimidines to CPD or 6–4 PP formation. **b** Example of a bound high-affinity NFY binding site structure predicted with AlphaFold 3 and visualized in PyMOL, using canonical human NFY protein sequences (ModelArchive ID: ma-wasserm-tfbs). The motif T^1^T^0^ and motif-complement C^−2^C^−1^ are highlighted as CPD and 6–4 PP hotspots, respectively, in bound and unbound states. Key atoms are annotated: C_5_ and C_6_ atoms for T^1^T^0^ and C_5_ and O_4_ for C^−2^C^−1^. **c** Boxplots of CPD-relevant structural parameters for T^1^T^0^ across NFY binding sites when bound by NFY (colored box plots, *n* = 60) versus TT steps in corresponding unbound structures (gray boxplots, *n* = 46). Only structures for which all DNA residues had a mean predicted local distance test score ≥70 were used in the analysis (“Methods”). Center line: median; boxes limits: first and third quartiles; whiskers: most extreme points within 1.5× the interquartile range. Red shaded regions signify the CPD-reactivity thresholds characteristic of TT photodimer formation in unbound, duplexed DNA reported by Conti et al.^33^. **d**, **e** As in **c**, but for positions of interest in the binding sites of ETS (bound C^3^T^2^ *n* = 58; unbound CT *n* = 99; bound C^4^C^3^ *n* = 20; unbound CC *n* = 177) and AP1 (bound T^−1^C^−2^ *n* = 19; bound T^1^C^2^ *n* = 19; unbound TC *n* = 70). CPD formation Z-scores calculated from a Poisson background model are shown above each plot. CPD-reactivity thresholds are not included here because their generalizability to non-TT dipyrimidines is unknown. **f** As in **c**–**e**, but for 6–4 PP-relevant structural parameters in the motif-complement T-tract in TBP binding sites (bound T^1^T^0^ *n* = 19, bound T^2^T^1^ *n* = 19, bound T^3^T^2^ *n* = 11, unbound TT *n* = 49). Red shading indicates 6–4 PP-reactivity thresholds for TT dipyrimidines from Conti et al.^33^. **g** As in **f**, but for C^−2^C^−1^ in NFY binding sites (bound C^−2^C^−1^ *n* = 59, unbound CC *n* = 30). **h** Pseudorotation angles and corresponding sugar pucker conformations for NFY, TBP, and LEF1 binding site positions with significantly enriched 6–4 PP formation. Gray markers are measurements from the equivalent positions in predicted unbound structures for the same binding site sequences. Source data are provided in the Source data file.

Despite the large number of TFs that exhibited both CPD and 6–4 PP modulation within their binding sites (*n* = 137), there is almost no overlap (*n* = 1) in actual position and effect between the 99 and 138 TF binding site positions with significant CPD or 6–4 PP signal, respectively. The only overlap was at the NRF1 binding site position 1/2 on the motif-complement strand, where both CPD and 6–4 PP formation enrichment is seen (Supplementary Data 3). The dissimilarity of these two groups reflects that, although both UV lesions form at dipyrimidines, the TF-induced structural distortion that predisposes DNA to CPDs versus 6–4 PPs is fundamentally distinct. Prior structural analysis of dipyrimidines in ETS and CTCF binding sites^17,22^, focused on shortened C_5_–C_6_ bond distance length (i.e., *d*_*22*_ distance) and decreased C_5_–C_6_ dihedral angle, based on molecular dynamics simulations reporting that these parameters capture the compaction and alignment of the adjacent C_5_–C_6_ bonds needed for CPD dimerization^30^. A more recent paper by Conti et al.^33^, expanded on the electronic and structural determinants of DNA photodimer formation in thymine dimers, reporting that base stacking was additionally important in optimal CPD formation conditions (Fig. 6a, top), while 6–4 PPs instead favor structural distortions that position dipyrimidines in an oxetane-like geometry, the key intermediate structure in TT 6–4 PP formation (Fig. 6a, bottom). For both UV lesion types, the authors emphasize that photoreactivity is induced by a nuanced interplay between these pro-damage features, where no one factor is the principal driver, or even universally necessary, for damage formation. Guided by this ensemble of structural parameters, we developed a multi-parameter framework for assessing whether TFs distort binding site DNA in a manner consistent with our CPD and 6–4 PP formation analyses.

Determining TF binding effects on photodimer formation requires structural characterization of binding sites in their bound and unbound states. However, few experimentally resolved TF-DNA structures have an accompanying unbound DNA structure for this comparison, and many lack UV-damageable dipyrimidines at positions of interest. To address this limitation, we leveraged AlphaFold 3 to systematically generate predicted TF-bound and unbound high-affinity binding site structures^52^ (“Methods” and Fig. 6b).

To compare against the TT photoreactive parameters described by Conti et al.^33^, we first examined position 0/1 on the motif-complement strand of the NFY CCAAT motif, which exhibits the largest induction of CPD formation for a conserved TT (Fig. 2c). In addition to *d*_*22*_ distance, we measured base ring overlap, base pair shift, and sugar puckering configurations of T^1^T^0^ in 20 high-affinity NFY binding site sequences. We then repeated the analysis for the same binding sites when bound by canonical human NFY (UniProt IDs: P23511, P25208, and Q13952)^53^ (Fig. 6c, left). Upon binding, NFY T^1^T^0^ undergoes a 4.3-fold increase in base ring overlap and a 7.9-fold increase in negative base pair shift. Additionally, the 3′ T^0^ exhibits a very small average sugar phase angle (68.36° ± 3.05), revealing an unusual C4′-exo sugar puckering. In contrast, the 5′ T^1^ has a larger sugar phase angle (144.35° ± 3.71), adopting a C2′-endo sugar pucker typical of B-DNA. This specific configuration of a large 5′ sugar phase angle followed by a small 3′ sugar phase angle, while not fully understood, has been reported as promoting CPD formation, potentially by increasing planar stacking between the bases^33^. These three pro-CPD structural parameters all meet or exceed the thresholds for TT CPD photoreactivity determined by Conti et al.’s quantum mechanical measurements. Although these thresholds should be interpreted with caution because they are determined from TTs in differing sequence contexts and thus subject to varying structural constraints, they illustrate how NFY T^1^T^0^ undergoes dramatically CPD-favorable distortions when bound. Surprisingly, this position does not show an appreciable decrease in *d*_*22*_ distance in the bound state, indicating that NFY binding is inducing CPD formation by stabilizing an optimal stacking of T^1^T^0^ rather than by directly shortening the distance between the C_5_–C_6_ bonds. As a control, we performed the same analysis for position 1/2 on the motif-complement strand of ETS binding sites, which also has a conserved TT but no significant TF-induced CPD formation enrichment (Fig. 1c). Consistent with this position being non-CPD-reactive, ETS T^2^T^1^ does not show the same prominent increase in base stacking geometry when bound by ETS (Supplementary Fig. 13a).

We continued our analysis for non-TT CPD hotspots. Consistent with literature^22^, the most extreme CPD formation enrichment at ETS position 3/4 on the motif-complement strand corresponded to increased base stacking, accompanied this time by a drastically shortened *d*_*22*_ distance in C^4^C^3^ upon ETS binding (Fig. 6d). Conversely, at ETS C^3^T^2^ there are the inverse structural trends, with a lengthened *d*_*22*_ distance, reduced base stacking overlap, and a positive base pair shift, such that ETS binding effectively moves the adjacent C_5_–C_6_ bonds apart and increases their misalignment at this position. This anti-CPD configuration provides a structural explanation for the significant depletion of CPD formation we noted previously (Fig. 1c). Similar CPD-inhibitory distortion was observed in AP1 binding sites, which showed the largest protective effect against CPD formation (Fig. 6e).

To evaluate how TF-binding induces 6–4 PP formation through distortion, we performed a comparable analysis for positions 0/1, 1/2, and 2/3 in the T-tract of the TBP binding site motif. TBP binding strongly enhanced 6–4 PP formation at T^1^T^0^ and T^2^T^1^ but no significant effect was observed at T^3^T^2^ (Fig. 5d). Here, to assess the alignment and proximity of the 5′ T C_5_ and 3′ T O_4_ necessary to create the oxetane intermediate that precedes 6–4 PP formation, we measured the average distance between 5′ C_5_ and 3′ O_4_ (*d*_*64*_ distance), interbase angle, and base pair slide (Fig. 6a, bottom) for both TTs across 20 high-affinity TBP sites. Both T^1^T^0^ and T^2^T^1^ show markedly increased interbase angles and large positive base pair slides when bound by TBP, far exceeding the 6–4 PP reactivity thresholds for TTs reported by Conti et al.^33^ (Fig. 6f). The *d*_*64*_ distance was also shortened in both TTs, with T^1^T^0^ reaching the 6–4 PP reactive range and T^2^T^1^, while technically outside of reactivity, still significantly shortened compared to TTs in the unbound state (Mann–Whitney *p* = 6.71e-08). Notably, T^3^T^2^, with the exception of having a very positive base pair slide, did not exhibit the same large structural trends. The relatively minor distortion at T^3^T^2^, while simultaneously being directly adjacent to highly 6–4 PP susceptible dipyrimidines at T^1^T^0^ and T^2^T^1^, may explain why there is such a small effect on 6–4 PP formation at the 2/3 position.

In addition to the CPD hotspot at T^1^T^0^, NFY also has extreme 6–4 PP formation enrichment at position −2/−1 on the motif strand (Fig. 6b, right). Because cytosines have an amine group at C_4_ rather than a keto group as in thymines, it is speculated that cytosine-containing dipyrimidines form 6–4 PPs through an azetidine-like intermediate that is similarly defined by proximity of the 5′ C_5_ and 3′ N_4_ of the amine group^54^. Indeed, the NFY C^−2^C^−1^ showed the same structural trends seen in the TBP TT hotspots (Fig. 6g), providing a striking example of simultaneous structural predisposition for both photodimer types in the same TF-DNA complex. Across the dipyrimidines we analyzed for structural enhancing 6–4 PP formation, we also noted atypical sugar puckering, reflective of extreme backbone distortion upon TF binding (Fig. 6h). Together, our structural analyses confirm and greatly expand on previous models of UV damage formation via TF-induced DNA structural distortions.

## Discussion

Here, we presented an analysis framework for high-throughput characterization of UV-induced DNA damage formation and repair in the active binding sites of ~600 human TFs. Our analysis of CPD formation largely agrees with prior studies that investigated smaller cohorts of TFs, particularly for TFs with the largest effects, like ETS and AP1^16,17,21–23^. However, we do see discrepancies at TF binding site positions with less pronounced effects, many of which were not reported previously to have significant enrichment or depletion of damage. A fundamental advantage of our approach is its control of sequence bias beyond just dipyrimidine identity, allowing us to isolate and provide statistical assessment of effects we can confidently attribute to TF binding, versus inherent sequence damageability of TF motifs (Fig. 1). Some of the differences between our results and previous work may stem from this sequence bias, or from a lack of statistical power to detect small but nevertheless significant effects. We are thus able to extend the compendium to TFs and binding site positions where TF binding is likely to have a direct effect of CPD formation (Supplementary Data 3).

We also provide an expansive, statistical quantification of TF binding modulation of 6–4 PP formation in human cells, using UVDE-seq data. As with CPDs, the frequency of 6–4 PP formation is greatly influenced by TF binding and has widespread effects across the TFs studied (Fig. 5). Importantly, though, the landscape of 6–4 PP damage susceptibility is distinct from CPD damage, with almost no overlap in positional effects between the two photodimer types. For both lesions, the effect of TF binding on their formation is entirely TF-, position-, and strand-specific, with many TFs showing both enrichment and inhibition at differing positions within their binding sites. We were able to identify 6–4 PP hotspots even outside of immediate TF binding sites, as in the case of CTCF, where we saw a prominent 6–4 PP formation enrichment approximately where cohesin is believed to interact for chromatin loop coregulation with CTCF^47–51^ (Fig. 5h).

Using AlphaFold 3-predicted TF-DNA complexes, we developed a flexible framework for assessing photodimer-favorable structural distortions induced by TF binding. This in silico method allows us to investigate TFs and binding site sequences that are unavailable as solved structures, greatly expanding the scope of our analysis. In addition, we extended prior structural work that assessed pro-CPD structural distortion of dipyrimidines in ETS and CTCF binding sites^17,22^ to include a set of structural features that have been reported to also be critical for CPD and 6–4 PP formation (Fig. 6a). While it has been assumed that 6–4 PP hotspots in TF binding sites are a consequence of TF-induced DNA distortions, similarly to CPD enrichment, a structural analysis demonstrating this relationship has not been performed until now. For the five TFs that exhibited some of the strongest modulation of UV damage formation (NFY, ETS, AP1, TBP, and LEF1), the affected binding site positions exhibited clear structural distortions. The observed structural trends match the expected effects for both damage formation enrichment and inhibition, underscoring the specific structural requirements necessary for the formation of either lesion type.

NFY provides an excellent example where, upon binding, both CPD- and 6–4 PP-favorable distortions are simultaneously induced at different positions in the binding motif (Fig. 6b) that correspond to CPD and 6–4 PP formation hotspots. The relative amount of each damage type within the NFY binding motif, and whether the two types of damage ever form at the same time within the same binding site, is unknown. Importantly, the structural distortion we saw at UV damage hotspots was rarely photoreactive across all structural parameters measured, indicating that there is a nuanced interplay in structure that leads to ideal conditions for photodimer formation. This multi-parameter nature of photodimerization is consistent with existing work that emphasizes that UV photodimerization is the result of an ensemble of structural parameters, none of which is the principal driver in all cases^33^. While AlphaFold 3 enables the scalability to generate a distribution of structural measurements for evaluation, it should be emphasized that in silico-based approaches come with limitations. During our analyses, we found that AlphaFold 3 prediction quality of TF-complexed DNA was inconsistent for TFs that had no experimentally solved TF-DNA structures available in the Protein Data Bank (PDB)^55^, which AlphaFold 3 uses for model training. Accordingly, we carefully selected TFs that have good representation in the PDB and restricted our analysis to structures predicted with high confidence (“Methods”).

It has been hypothesized that TFs attenuate DNA repair by directly competing with repair factors for lesion recognition. While this mechanism has been recently demonstrated for mismatch repair of DNA replication errors^56^, direct evidence of TF-repair competition, on a per TF basis, has not been established for NER of UV-induced damage. Our study provides candidate TFs and binding site positions where TF-NER competition is likely to happen, and subsequently lead to increased mutagenesis. Using timecourse CPD-seq v2.0 datasets, we performed simulations of CPD levels over time to statistically evaluate TF binding site positions of reduced repair activity. Similar to our analysis of UV damage formation, our approach carefully controlled for sequence composition of TF binding sites, as we and others have shown that repair efficiency is dramatically impacted both by dipyrimidine type and by sequence context^57,58^ (Supplementary Fig. 9). We identified 75 TFs that appear to attenuate CPD repair and, equally importantly, identified positions of repair reduction within their binding sites (Fig. 4). The repair attenuation we see is limited to specific positions within TF binding sites, and is specific to the TF (or the TF cluster/family) in question. While at first glance, our results may seem to contrast with previous studies that reported much more widespread NER attenuation of UV damage in TF binding sites^5,9,15,16,21^, this difference actually reflects a key methodological distinction that extends and complements what has been previously reported. Most prior studies using XR-seq data examine repair in TF binding regions at a general level because XR-seq reads are difficult to map precisely to original lesion positions. With the resolution of CPD-seq v2.0 timecourse data, we were able to focus our analysis to the TFs’ binding sites on a single-nucleotide basis and differentiate effects that are attributable to direct TF binding rather than the local repair environment outside the TF’s immediate binding footprint. As such, in this study, we interrogated the mechanistic model of TFs attenuating repair specifically by binding to UV-lesions within their binding site. Importantly, our results do not preclude other mechanisms by which TFs may be contributing to reduced NER activity around their binding sites more broadly.

As an additional source of discrepancy, the effect of sequence context on DNA repair has also not been incorporated in prior methods, which is critical for differentiation of TF binding effects as opposed to slow repair inherent to the sequence composition of the binding motif being studied. We see this for the mutation peak in CTCF binding sites (Fig. 4f), where although there are high levels of CPDs after 6 h of repair, these levels are within the expected range given that the CTCF binding motif consists of sequence content that is poorly repaired in general. Overall, our results better align with a model where CPD damage is only tolerated and successfully bound when it occurs at specific binding site positions, based on the TF-specific binding dynamics in question. This suggests that TFs bound to sites with UV damage at these tolerated positions may be able to compete with factors like XPC and UV-DDB, preventing repair initiation and driving mutagenesis.

However, it is important to note that our analysis of repair covers only the first 6 h post irradiation, whereas there is evidence that CPDs continue to be repaired for up to 48 h^59^. Therefore, there could be additional repair attenuation effects that are only observable at later timepoints, outside of our current analysis’ repair window. Throughout our genomic analyses, TFs that do not exhibit significant effects may also be lowly expressed or have limited nuclear occupancy in the given cellular context. Notably, ~70% of TF clusters used in our analyses include at least one TF expressed in human skin fibroblasts (RNA-seq TPM ≥ 1, “Methods”), and TF clusters showing significant modulation of CPD formation, CPD repair, and/or 6–4 PP formation were significantly enriched for TFs with a TPM ≥ 1 (one-sided Fisher’s exact test, *p* = 4.84 × 10e-6, “Methods”). These observations suggest that the full scope of TF binding effects on UV damage formation and repair may be broader than what we observed here and could be further expanded by examining additional cell types in which distinct sets of TFs are expressed.

Furthermore, because we carefully curated our analysis for TF binding sites in cis-regulatory regions, we cannot assume that TFs modulate UV damage formation and repair uniformly in other parts of the genome. TFs that bind nucleosome-rich DNA, like pioneer TFs, or those that interact with gene bodies, which are subject to TC-NER, may exhibit distinct effects that warrant future studies.

**Fig. 7:**
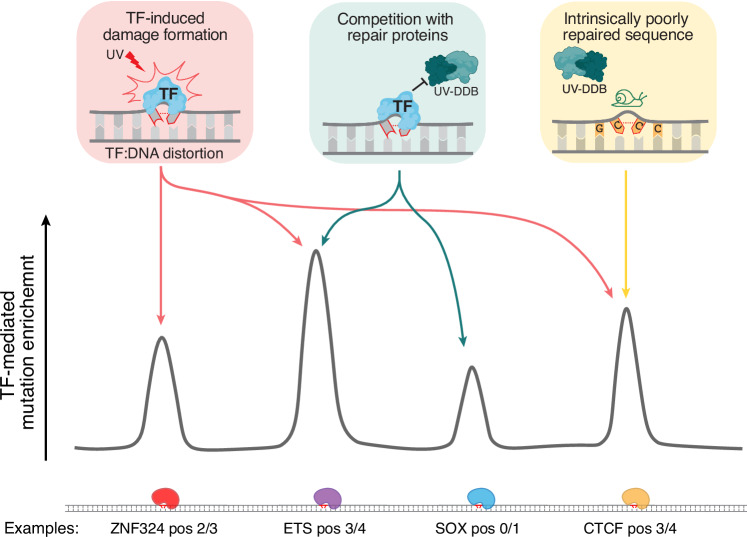
Synergistic mechanisms drive UV-linked mutagenesis in TF binding sites. Conceptual illustration of how TF-induced damage formation, TF-repair competition, and intrinsically inefficiently repaired TF binding site sequences contribute, and together compound, UV mutagenesis. Examples of TF binding site positions for each mutagenic scenario are shown at the bottom. Created in BioRender. Wasserman, H. (2026) https://BioRender.com/5fyrhno.

Finally, we compared the output of our analyses with actual mutations from skin cancers to infer the likely mechanism driving observed mutation enrichment in TF binding sites. Instead of demonstrating a single dominant mechanism, this analysis reveals a complex mosaic of TF-induced mutagenicity, where the mechanistic driver of mutation enrichment is contextual, dependent on the TF, position, and DNA sequence of the damaged locus itself (Fig. 7). For example, in SOX binding sites we observe two different positions of mutation enrichment, one that directly coincides with repair attenuation while the other is seemingly due to enhanced CPD formation (Fig. 4d). Strikingly, the strongest mutation enrichment we found in TF binding sites was often associated with multiple sources of mutagenicity. The C>T mutation peak at position 3 in ETS binding sites, which has the largest enrichment z-score according to our analysis, exhibits CPD formation enrichment in conjunction with repair attenuation, creating a hyperaccumulation of mutagenic CPDs that are then occluded from efficient repair (Fig. 4e). Similarly, the second strongest mutation enrichment peak at position 4 in CTCF binding sites, while having expected levels of repair, occurs at GCCC, which is an extremely inefficiently repaired tetranucleotide sequence in general (Supplementary Figs. 9 and 11b, c). Combined with the substantial burden of CPD damage at this position, from initial CPD formation enrichment, the inefficient repair leads to a highly pronounced mutagenic hotspot (Fig. 4f). We note that our analysis focused on single base substitution (SBS) mutations, and whether these TF-driven effects on CPD formation and repair contribute to mutations of other kinds, like insertion/deletion events, remains to be explored.

In contrast to CPDs, the mutagenicity of 6–4 PPs is less obvious, and we did not observe strong mutation enrichment that coincided solely with 6–4 PP enrichment. This is consistent with prior studies, which reported that 6–4 PPs are repaired with high efficiency by global genomic nucleotide excision repair (GG-NER)^40,60–62^. Future analysis using UVDE-seq timecourse experiments will allow us to verify this and identify mutagenic positions of repair reduction, as we did with CPDs. Interestingly, while not directly at positions of 6–4 PP or CPD formation enrichment, we see cases of C>T mutation enrichment adjacent and 3′ to UV damage formation hotspots in TF binding sites (e.g., LEF1, Fig. 5f). Collateral mutagenesis, whereby low-fidelity translesion polymerases introduce nucleotide misincorporations adjacent to DNA lesions, has been described in eukaryotes and provides a possible mechanism for these UV damage-proximal mutation peaks^63^. A study of lesion segregation in mouse models further characterized this process, reporting that collateral mutations are enriched within 1–2 base pairs of the original DNA lesion, which is consistent with what we observe^64^. Additionally, in certain contexts, such as LEF1 (Fig. 5f), the observed enrichment of C>T mutations adjacent to damage hotspots could theoretically be influenced by CpG methylation on the opposite strand, which is not incorporated in our current model and is an area for future study.

When we examined base substitution mutations beyond C>T, we found instances of general SBS enrichment that occur in the vicinity of UV damage hotspots (e.g., NFY, Supplementary Fig. 12). Recently, a model of mutagenic NER was proposed where a DNA lesion serves as a low-fidelity template during the resynthesis step of NER if it occurs within ~30 base pairs (within the excision bubble) of another DNA lesion on the opposite strand^64^. Co-occurring CPD and 6–4 PP hot spots in TF binding sites that are efficiently repaired by GG-NER may be particularly vulnerable to this mutagenic process, as illustrated by NFY binding sites, where the high burden of UV damage immediately after irradiation is efficiently repaired, theoretically driving extensive mutagenic NER resynthesis and resulting in the general mutation enrichment we observe in NFY’s flanks.

Rigorous, high-throughput studies like ours have the capacity to offer a careful and comprehensive view of the mutagenic dynamics between UV damage and TF binding. In addition to enabling the evaluation of previously uncharacterized TFs, our approach also uncovers broad trends, showing the remarkable diversity in how TF binding influences both damage formation and repair. Throughout our study, we emphasize the importance of controlling for underlying DNA sequence context in TF binding site analyses, which, although intuitive, is essential for accurate interpretation of these effects. With this high-throughput perspective, we can also identify unexpected patterns of UV-linked mutagenesis that would be otherwise difficult to classify as trends when looking at single TFs. The resulting catalog of TF binding effects we provide in this work serves as a valuable resource for future studies, as it identifies TFs that are likely to directly influence mutagenesis in cancer cells by modulating damage formation or repair. Mechanistic characterization of such TFs, and determination of which mutations in their binding sites can versus cannot be enriched due to TF binding, will be critical for future studies to identify driver regulatory mutations in cancer, i.e., mutations enriched beyond a background model that includes the contribution of TF binding to mutagenesis. Furthermore, the methodology we developed in this study can be readily extended to other types of DNA damage, as seen with our recent analysis of smoking-induced damage in TF binding sites^65^, offering a robust, flexible framework for dissecting the complex interplay between TFs, DNA damage, and repair, and mutagenesis.

## Methods

### Pre-processing of CPD-seq v2.0 datasets

All CPD-seq v2.0 datasets used in our analysis were processed from human skin fibroblast CS1AN/CSB^WT^ cells and originally published by Duan et al.^27^ (GEO accession GSE235483). The CPD-seq v2.0 BED files used were pre-filtered to exclude any CPDs that did not map to dipyrimidine sequences. CPDs were mapped to genome assembly GRCh37 (hg19) using custom Python scripts and BEDTools (v2.31.0)^66^. Genomic positions with multiple CPDs were summed for an aggregate damage count at that position. CPDs in ENCODE hg19 blacklist regions were excluded^67^.

### Generation and analysis of ATAC-seq data

To map open chromatin regions best suited for our analysis, four biological replicates of the human skin fibroblasts CS1AN/CSB^WT^ cell line were prepared with the Omni ATAC-seq protocol, using approximately 50,000 cells per library^68^. Libraries were sequenced at the Duke Sequencing and Genomic Technologies shared resource core. Sequencing QC metrics and metadata for these libraries can be found in Supplementary Data 6. Raw ATAC-seq reads were processed using the ENCODE ATAC-seq-pipeline (v2.2.3) and the hg19 reference genome for irreproducibility detection rate (IDR) analysis of the four biological replicates^69^. Tn5 transposase offset correction was performed as part of the ENCODE pipeline workflow. Per ENCODE pipeline recommendations, conservative IDR peaks were called with MACS2 using a threshold *p* < 0.01 and a subsequent IDR threshold of 0.05 (GEO accession GSE309644). For high-quality open chromatin regions, we used the top 50% of ATAC peaks (*n* = 39,312) based on peak signal (fold enrichment above background) and statistical significance for further analysis. We defined open chromatin regions as 150 bp windows centered around the summits of the ATAC peaks to further enrich for the most accessible regions of the ATAC-seq peaks. To avoid structural and repair confounders related to active transcription (e.g., single-strandedness, transcription-coupled NER), we also subtracted coding and noncoding genic regions (exons and introns) from the accessible dataset, as defined by the UCSC RefSeq Table Browser for hg19 genome assembly^70^.

### Curation of active TF binding sites

Active TF binding sites for the CS1AN/CSB^WT^ cell line were curated from binding site calls based on nonredundant motif clusters previously reported in Vierstra et al.^29^, which were derived from human and mouse motif models. Due to naming redundancy, four clusters (SMAD, ZNF143, and ZNF85) were removed, of which the cluster that best represented human protein orthologs was kept. Three clusters were removed due to low motif model specificity (KLF/SP/2, KLF/SP/3, and GC-tract). Finally, two clusters (ZNF431 and HD/24) were removed because they lacked a human ortholog in the motif model. The binding sites of the remaining clusters were converted from GRCh38 (hg38) assembly using the UCSC Genome Browser LiftOver tool^70^ for compatibility with hg19. These sites were intersected with genome-wide DNA accessibility data from CS1AN/CSB^WT^ cells to ensure curation of active binding sites in chromatin accessible regions. There were in total 3,693,180 chromatin accessible binding sites for the 225 TF clusters in our study, of which 2,329,615 (63.0%) were in intergenic regions and considered for our analysis. For each motif cluster, the TF binding site calls were ranked by their motif scores using Motif Occurrence Detection Suite (MOODS)^71^, and the top 50% of sites with the highest MOODS scores (including ties) were used for further analysis to enrich for bound sites. For the CTCF motif cluster, we further limited analysis to the top 10,000 sites based on MOODS score, after noting that the top 50% of sites still included a large proportion of unbound sites (Supplementary Fig. 4). Motif clusters that did not have at least one position with an aggregate 30 CPDs were filtered out as our limit of detection for identifying statistically significant trends. From this preprocessing, a total of 225 motif clusters covering 597 distinct human TF proteins were analyzed for the study (Supplementary Data 1). We note that included in these motif clusters are motif models for TFs that are believed to bind to DNA indirectly as co-factors (e.g., SMARCA1), however, these TFs that are indirect binders represent a small minority of the motif models included in our curation. We also note that not all TFs in a particular cluster are expressed in any particular cell type. Thus, when identifying a cluster as modulating UV damage or repair based on the analyses described here, we interpret this to mean that all TFs in the cluster have the potential to modulate damage/repair, but whether they do so or not will depend on the particular cell type, and more specifically on the expression level and activity of each TF in that particular cell type.

We also considered using RNA-seq gene expression data when curating active TF binding sites. Given that RNA-seq data is not available for the exact cells used in our analyses, we instead used human skin fibroblast RNA-seq data from the ENCODE consortium (ENCSR000CUH), with reported posterior mean estimates for transcripts per million (TPM). At the cluster level, we found that a majority of the 225 TF motif clusters contain at least one expressed TF: ~70% of clusters (156) contain at least one TF with TPM ≥ 1, and ~79% of clusters (177) contain at least one TF with TPM ≥ 0.5. We chose not to use TPM values to filter out TF clusters because: (1) it is unclear what gene expression level (or, more precisely, nuclear protein level) is needed for a TF to have a significant effect on UV damage formation or repair; (2) it could be that additional TFs should be assigned to these clusters in the future; and (3) some TFs that are not expressed based on the ENCODE RNA-seq data could be expressed in the cells used to generate the CPD-seq data. Interestingly, when comparing TF motif clusters with vs. without significant effects on CPD formation, 6–4 PP formation, or CPD repair, we found that clusters with significant effects are significantly more likely to contain TFs with TMP ≥ 1 (one-sided Fisher’s exact test *p* = 4.84e-06). In particular, ~87% (73/84) of the clusters identified as significantly modulating CPD formation, 6–4 PP formation, and/or CPD repair contain at least one TF with TMP ≥ 1. Among the 141 clusters without any significant effects, only ~59% (83/141) contain at least one TF that meets this expression threshold. The gene expression data used in this analysis are included in Supplementary Data 1.

### Poisson modelling of CPD formation at TF binding sites

We consider the formation of CPDs as a stochastic Poisson process that consists of discrete, independent rare events where event frequency is dependent on the sequence context of a central dipyrimidine. CPD formation frequencies for all 1024 dipyrimidine-centered hexanucleotide sequences (NNYYNN) were calculated by intersecting human skin fibroblasts CPD-seq data with human skin fibroblasts-specific chromatin accessible regions and dividing total damages by the number of occurrences for each hexanucleotide (Supplementary Data 2). We calculated the expected number of CPDs over k occurrences of a given NNYYNN, n, as, ${r}_{n}k=E[{X}_{n}]={\lambda }_{n}$, where ${r}_{n}$ is the rate of CPD formation for n. By considering the CPD formation rate of each NNYYNN as its own independent Poisson distribution, we were able to leverage the property for sums of Poisson-distributed random variables^72^ to estimate the cumulative amount of damage per position for both motif and motif-complement DNA strands in TF motif clusters. Kolmogorov–Smirnov tests confirmed that each NNYYNN sequence conformed to our proposed Poisson model of CPD formation in irradiated cells (Supplementary Fig. 2). Interestingly, a similar analysis of CPD-seq data in naked DNA revealed that CPD formation does not adhere to the same statistical assumptions, with several NNYYNN sequences deviating significantly from a Poisson process (Supplementary Fig. 5a). Thus, we chose to use only the cellular CPD-seq data to derive our background model of CPD formation.

Active TF binding site windows were defined as ±40 base pairs relative to the center of the TF cluster binding motif. CPD damages were intersected with these windows and summed to determine the cumulative amount of CPD damage per position for both motif and motif-complement DNA strands separately. For each position in the TF binding sites window, we compared the observed CPD counts to the predicted number of CPDs according to our Poisson background model of CPD formation. The predicted amount of cumulative damage was calculated separately at each position and strand in the TF binding site window. The predicted CPD signal for each strand was then scaled by multiplying the predicted CPDs by the average ratio of observed-to-predicted CPD signal in the immediate flanking regions around the TF motif. For compatibility with TF motif clusters exhibiting differing amounts of flanking damage within the same strand on either side of the TF motif (possibly due to palindromic conserved sequence content or co-factor activity), this scaling was done separately for each half of the TF motif. After scaling, a one-sided p-value for the observed CPD signal at each position in the TF binding site window was calculated under a Poisson model. The Benjamini–Hochberg procedure was then used across each TF’s binding site window to correct for multiple testing.

### Analysis of CPD projected versus reported melanoma mutation profiles

Somatic skin cancer mutations were curated from the International Cancer Genome Consortium (ICGC), release 28^39^. Mutations were downloaded from ICGC from projects SKCM-US, MELA-AU, and SKCA-BR from 183 skin cancer donors and filtered for C>T SBS in whole genome sequencing. Mutations that mapped to the TF binding site windows from our CPD formation analysis were used. C>T mutation profiles and statistical significance of mutation enrichment/depletion were determined using a simulation-based approach that shuffled all C>T mutations from open chromatin regions by their trinucleotide sequence context to create a null distribution of expected mutations at each position. This was performed identically to the orthogonal simulation-based method leveraged for CPD formation. Expected mutations per the background model were scaled to average mutation levels in local DNA flanking the TF binding sites. See Supplementary Methods for further details. For mutation analysis of base substitutions generally (not just C>T, e.g., NFY in Supplementary Fig. 12), we performed an analogous analysis using an alternative method by Sahay, H (available at [https://github.com/harshitsahay/TFBS_Mutations]). Briefly, this method evaluates the statistical significance of mutation enrichment across all base substitution types by comparing against a background model where mutations are assumed to be a Bernoulli process and estimated using a Poisson binomial model conditioned on trinucleotide context. We again used mutation data from ICGC for this analysis.

Projected C>T mutation profiles were generated by assuming all CPD-dimerized cytosines from CPD-seq v2.0 of human skin fibroblasts processed immediately after 6 J/m^2^ UVC irradiation were mutated to thymines (See Supplementary Fig. 8 for details). We used ordinary least squares regression on projected versus actual skin cancer mutations to determine the best-fit line and identify positions where C>T mutations projected from initial CPD formation were significantly discordant (${{{\rm{abs}}}}$(z-score) > 2.58) with reported mutation enrichment. For the “random TF” right-hand violin plot in Fig. 3a, the Pearson correlation coefficients were generated by comparing the actual skin cancer C>T mutation profile of a given TF cluster with the projected C>T mutation profiles of 5 randomly selected TF clusters. Each marker represents the average Pearson correlation coefficient between a given TF cluster and these 5 randomized comparisons. All Pearson correlations were computed across TF binding site positions and their flanking regions (±20 bp relative to the center of the binding site).

### Simulation of CPD repair at TF binding sites

Expected CPD levels in human skin fibroblasts after 6 h of repair were simulated from CPD-seq timecourse data with 10,000 bootstrap samples per position along the binding site windows of each TF. To reduce computational workload, the TF binding site windows were narrowed to ±30 base pairs relative to the center of the TF cluster binding motif. Because we found that tetranucleotide sequence context around a central dipyrimidine (NYYN) significantly impacted CPD repair efficiency (Supplementary Fig. 9 and Supplementary Table 1), the bootstrapping at each position was conditioned both on the initial amount of CPDs formed and NYYN composition (Supplementary Methods). CPDs used to model the background were derived from intergenic, open chromatin regions. Genic regions were removed to avoid the confounding effects of transcription-coupled NER (TC-NER). As with all our CPD-seq analyses, we scaled our predictions relative to the signal in flanking DNA around each TF cluster’s binding sites, to focus on effects of TF binding rather than local sources of potential repair variation (e.g., proximity to nucleosomes or transcriptional activity). We then calculated empirical one-sided *p*-values from the simulations for each position and strand along the TF binding site window. Benjamini–Hochberg multiple test correction was then used across all positions in the TF binding site window.

### Generation and processing of UVDE-seq data

We adopted the emRiboSeq protocol^73^ and our own UVDE-seq publication in yeast^43,44^ to accurately map 6–4 PP in human skin fibroblast cells (NHF1 cell line). Briefly, cells were grown to confluence in Dulbecco’s modified Eagle’s medium (DMEM) containing 10% fetal bovine serum at 37 °C and 5% CO_2_. Prior to UV exposure, the media was removed, and the cells were rinsed with 1× phosphate-buffered saline (PBS). Two milliliters of fresh PBS was then added, and cells were exposed to 500 J/m^2^ UVC light based on previous calibration. Cells were then treated with trypsin, removed from the dish, and pelleted. Genomic DNA (gDNA) was pelleted and isolated with GenElute Mammalian Genomic DNA Miniprep kits (G1N70, Sigma-Aldrich), as described in the manufacturer’s protocol. Cells used for naked DNA samples were collected as described, but not exposed to UVC. Following gDNA isolation, naked samples were spotted onto a microscope cover slide on ice and exposed to 400 J/m^2^ UVC light. All samples were then sonicated, and DNA fragments were treated with end-repair and dA-tailing modules (New England Biolabs), and a double-stranded trP1 adapter was ligated to both ends of the fragments. Adapter and primer sequences (used to confirm ligation) are listed in Supplementary Methods. Following trP1 ligation confirmation, free 3′-OH groups were blocked with Terminal Transferase and dideoxy GTP. Samples were then treated with CPD photolyase in the presence of uracil DNA glycosylase inhibitor (UGI), under 365 nm UV light for 2 h at room temperature. DNA fragments were purified and treated with *Thermus thermophilus* UVDE for 45 min at 55 °C. 5′ phosphate groups were removed using shrimp alkaline phosphatase, and DNA was subsequently denatured at 95 °C for 5 min and snap-cooled on ice. A second double-stranded adapter, the A adapter, was then ligated to the 3′-OH created immediately downstream from the cleaved UV lesion and PCR confirmed. DNA containing the biotin label on the A adapter was purified using Streptavidin beads, while the DNA strand lacking the biotin label was removed using 0.15 M NaOH. The remaining single-stranded DNA was then used as a template for second-strand synthesis, and libraries were PCR amplified for ~8 cycles using primers complimentary to the trP1 and A adapters. Samples were then combined and submitted for Ion Proton sequencing (Life Technologies) on an Ion Torrent S5 instrument.

The resulting UVDE-seq sequencing reads were trimmed of barcode sequences and the 3′ nucleotide of the sequencing read, and then aligned to the human (hg19) genome using Bowtie2 software^74^. The corresponding dinucleotide damage sites were identified and counted, as previously described^43,44^. Briefly, aligned reads were processed with SAMtools^75^ and BEDtools^66^, and custom Perl scripts were used to identify the dinucleotide sequence immediately upstream of the 5′ end of each sequencing read. Damage coordinates that mapped to 6–4 PP lesion-forming dipyrimidine sequences (i.e., TT, TC, CT, and CC) were retained for our analyses. See Supplementary Fig. 15 for the complete counts of reads mapped to each dinucleotide type.

### Analysis of 6–4 photoproduct formation at TF binding sites

For our analyses of 6–4 PP formation, we used UVDE-seq cellular DNA replicate 1 (GEO sample accession GSM8986610) and UVDE-seq naked DNA replicate 1 (GEO sample accession GSM8986612). Using custom Python scripts, genomic loci with multiple 6–4 PPs were summed for an aggregate damage count at the given loci. 6–4 PPs in ENCODE hg19 blacklist regions were excluded^67^. We found that, like CPDs, extended sequence context significantly impacted 6–4 PP formation frequency (Supplementary Fig. 1c, d). Therefore, we adapted the previously described hexanucleotide-based Poisson modelling approach used for assessing CPD formation to similarly analyze 6–4 PP formation in TF binding sites (Supplementary Fig. 2). Because a higher UVC dose was required in the UVDE-seq experiments to generate sufficient 6–4 PP signal, we found that UVDE-seq data exhibited more background signal than CPD-seq data. Therefore, we set a more conservative significance threshold across all 6–4 PP analyses, where positions with a BH corrected *p* < 0.01 are considered significant. In the Fig. 5a heatmap summarizing high-confidence results, we additionally spot-checked for type 1 error by performing the same analysis on UVDE-seq data from naked DNA and excluding any significant results that were also present at the same position in the naked DNA condition.

### Structural analysis of AlphaFold 3 predicted TF-DNA complexes

To generate a distribution of structural parameters from representative binding site sequences of TF motif clusters, the top 20 non-redundant binding sites based on MOODS score were selected for each position and dipyrimidine type of interest. Structural measurements were then aggregated across all generated structures containing the sequence feature of interest for the given TF. Therefore, the number of observations for a specific sequence feature can exceed 20, particularly for dipyrimidine positions in conserved motif regions, where most binding site sequences will contain that sequence feature. Each binding site sequence was 20 base pairs long and centered at the TF motif center. Supplementary Data 5 contains the full list of binding sites and protein sequences used, curated from the UniProt Knowledgebase^53^. These sequences served as input to the AlphaFold 3 server web application^52^ to generate free and TF-bound versions of the binding site sequences. For all structures, 3 base pairs at either DNA chain termini were excluded from further analysis due to the consistently lower confidence predictions observed at these positions for all predicted DNA (Supplementary Fig. 13c). To filter for quality AlphaFold 3 predictions, the per-atom predicted local distance different test (pLDDT) score was averaged across each DNA residue for the top-ranking structural prediction per AlphaFold 3 run. Only structures for which all DNA residues had a mean pLDDT score in the “confident” or above range (pLDDT ≥ 70) were retained for further analysis. Base-step *d*_*22*_ distance, *d*_*64*_ distance, of dipyrimidines were computed for each base-step per DNA strand using custom PyMOL Python scripts^76^. *d*_*22*_ was defined as the distance in Ångstroms (Å) between the C_5_–C_6_ bond midpoints between adjacent pyrimidines. d64 was defined as the Å distance between the 5′ pyrimidine’s C_5_ and X_4_ (either O or N) attached to the 3′ pyrimidine’s C_4_. Step base stacking, base pair shift, base pair slide, interbase angle, pseudorotation angle, and sugar puckering classifications of nucleobases were computed using X3DNA-DSSR (v2.5.0)^77^. Base stacking was defined as the overlapping polygon area in Å^2^ when projecting the dipyrimidine base ring atoms (excluding exocyclic atoms) into the mean base pair plane^78^. The sugar ring pseudorotation phase angle of each pyrimidine was also calculated using X3DNA-DSSR as described by Altona and Sundaralingam^79^. Interbase angle was defined as ${{{\rm{interbase}}}}\; {{{\rm{angle}}}}=\scriptstyle\sqrt{{{{{\rm{propeller}}}}}^{2}+{{{{\rm{buckle}}}}}^{2}}$ per the X3DNA-DSSR documentation.

### Statistics

Throughout our analyses, we report z-scores (number of standard deviations from the predicted mean) to communicate the magnitude of observed effects, alongside *p*-values, which communicate statistical significance. Where noted, the Benjamini–Hochberg procedure was used correct for multiple testing using Python’s statsmodels.stats.multitest module (v0.14.0). Statistical tests were performed in Python (v3.11) using scipy.stats (v1.13.1) implementations of kstest, kruskal, mannwhitneyu, and fisher_exact for Kolmogorov–Smirnov, Mann–Whitney *U*, Kruskal–Wallis, and Fisher’s exact tests, respectively. Ordinary least squares regression was performed using statsmodel.formula.api (v0.14.0).

### Reporting summary

Further information on research design is available in the Nature Portfolio Reporting Summary linked to this article.

## Supplementary information

Supplementary Information

Description of Additional Supplementary Files

Supplementary Data 1

Supplementary Data 2

Supplementary Data 3

Supplementary Data 4

Supplementary Data 5

Supplementary Data 6

Reporting Summary

Transparent Peer Review File

## Source data

Source Data

## Data Availability

The UVDE-seq data generated in this study have been deposited in NCBI’s Gene Expression Omnibus under GEO Series accession number GSE297236. The ATAC-seq data generated in this study for the CS1AN/CSB^WT^ cell line have been deposited in NCBI’s GEO under the GEO series accession number GSE309644. AlphaFold 3 structural predictions have been deposited in ModelArchive (https://www.modelarchive.org/) under Project ID ma-wasserm-tfbs [https://modelarchive.org/doi/10.5452/ma-wasserm-tfbs]. Previously published CPD-seq v2.0 datasets used in this study are available in NCBI’s GEO under GEO series accession GSE235483. Source data are provided with this paper.

## Code availability

Code used for the analyses described in this study can be found at GitHub (https://github.com/HanaWasserman/UV_damage_TFBS_analysis).
