# Supplementary material for: High-throughput characterization of transcription factors that modulate UV damage formation and repair at single-nucleotide resolution
Source: Nat Commun. 2026 Jul 10;17:8506. doi: 10.1038/s41467-026-75115-4 (PMC13483863; doi:10.1038/s41467-026-75115-4)
Supplement: Supplementary file 1 [file 41467_2026_75115_MOESM10_ESM.pdf]

nature portfolio

Peer Review File

High-throughput characterization of transcription factors that modulate UV damage formation and repair at single-nucleotide resolution

Corresponding Author: Dr Raluca Gordan

Parts of this Peer Review File have been redacted as indicated to maintain the confidentiality of unpublished data.

Parts of this Peer Review File have been redacted as indicated to remove third-party material.

This file contains all reviewer reports in order by version, followed by all author rebuttals in order by version.

Version 0:

Reviewer comments:

Reviewer #1

(Remarks to the Author)
The study by Wasserman et al provides a high-resolution analysis of how transcription factor binding modules the DNA damage and repair at different positions within and immediately adjacent to the TFBS across multiple TFs. The statistical framework implemented to quantify damage formation and repair is rigorous, and the results obtained at the base-pair resolution are novel, as compared to previous studies. Overall, the manuscript is well-written, and the results support the conclusion. The following are some minor comments for improvements:

a) Fig 1a) The number of active ETS binding sites reported was 15,533. whereas in Fig C, the total ETS binding sites reported were 31,153. Please clarify.

b) Fig 2f) the CPD formation/enrichment signal at position -4/-3 and -3/-2, directly 5' to its CCAT-binding motif, is interesting, despite the low conserved dipyrimidine at these sites. Given that the number of binding sites of YY1 is relatively low (as compared to ETS or CTCF), how confident is the CPD prediction for those low numbers of sites?

c) Why different SD value was used in Fig 1c(4SD) and Fig 2c-h (3SD)?

d) Fig 3a) The Pearson correlation between the projected and observed C>T mutations is unsurprising, because the sequence context is shared by CPD and mutations are similar. In other words, the observed correlation could be simply due to differences in the CPD formation/mutation frequency among the different hexanucleotide contexts (as shown in Fig. 1b).

e) Also, it is unclear how the "Random TF" was calculated in Figure 3a. The method says, "For the Pearson correlation coefficients of projected versus actual mutation profiles shown in Fig. 3a, the distribution of randomized comparisons was generated by calculating the correlation coefficients of a projected mutation profile of actual mutation profiles of 5 randomly selected TFs. Each marker represents the average of the five comparisons.". Does the plot cover only those 5 randomly selected TFs or all TFs, and what were compared in the Pearson correlation?

f) In Fig 4f, the observation that the mutation enrichment at positions 3 and 4 is not significantly explained by the repair reduction contradicts the observations by Poulous et al., Cell Report, 2016. Though this could be driven by the sequence context NYYN, the repair can still be further perturbed by the CTCF binding. In Figure 4h, can the All NYYN be separated by NYYN covered by any TF bound versus unbound to further support this observation?

g) Fig 5f, in LEF1 mutation profile, could the enrichment of C>T at the position in neighboring sites be explained by potential CpG methylation on the opposite strand?

(Remarks on code availability)

Reviewer #2

(Remarks to the Author)
This manuscript by Wasserman et al. presents a thorough analysis of the effects of transcription factor binding on UV DNA damage in skin cancers. The authors use recently developed high resolution techniques to map DNA damage, CPD-seq v2.0 and UVDE-seq, and perform statistical modeling to identify sites where TF binding enhances or protects against DNA damage. They also nicely compare this work in cells to mutations in skin cancer patients. Additionally, they perform CPD-seq v2.0 6 hours post UV damage, and use this time course data to model how TF binding affects repair of DNA damage. They also use AlphaFold3 to model TF-bound and unbound DNA structures to suggest how TF binding can alter DNA geometry to promote damage formation. This work is well done and nicely expands on previous work characterizing how ETS and CTCF binding affects DNA damage, now extending this work to many more classes of DNA binding factors.

My major comment on this work is that the authors claim that they can confidently attribute their findings to TF binding, versus inherent sequence features of the DNA. However, there are no direct measurements of TF binding. Given that the authors are measuring the effects of hundreds of TFs, it would be unreasonable to perform ChIP-seq experiments. The authors did perform CPD-seq and UVDE-seq on naked DNA, and I suggest that the results from these control experiments be shown more clearly in the paper. For example, the analysis shown in Figure S2B is very helpful and should be moved to the main body. The authors claim on lines 157 and 158 that "none of the significant peaks of CPD enrichment or depletion observed in cellular data were present in naked DNA", but they do not present this analysis beyond what is shown for ETS and CTCF in Figure S2. The authors should present similar analysis for the UVDE-seq on naked DNA to accompany Figure 5.

Also, are there any RNA-seq data sets the authors could use to assess TF expression in this cell type? The authors state that this analysis was performed on 597 TFs across 225 clusters, but it is now clear if all 597 TFs are in fact expressed and active in these cells. Given the subtle differences between how TFs within a same family interact with DNA, it is likely that not all TFs within these classes would have the same effects. If the expression data exists, the authors could add this information to Figure 2a. If not, they should add some additional text clarifying that the TFs in these clusters are the TFs that could bind these motifs, possibly in the paragraph containing lines 214-221.

(Remarks on code availability)
The README file for section 4 (analyze repair simulations) is not complete.

Reviewer #3

(Remarks to the Author)
The manuscript by Wasserman et al. describes a comprehensive analysis of the effect of TFs on DNA damage formation in human cells. There have been multiple studies addressing the effect of a limited set of TFs on UV damage formation, focusing primarily on the ETS and CTCF factors and CPD damages. Here, Wasserman et. al. applied a thorough statistical framework and curation of high-quality and high-resolution TF binding and DNA damage data to analyse a comprehensive set of TFs and identify previously un-reported effects (both positive and negative) on damage formation. Furthermore, the apply structural analysis of TF-bound DNA to explain the observed positive and negative influence on damage formation, and link their observation to melanoma mutagenesis data, showing how both damage susceptibility and repair efficiency contribute to the final mutagenic patterns.

I find the manuscript to be clearly written and have no strong critiques. I recommend acceptance to Nature Comm after addressing the following comments:

1) At the end of p.3: "The frequency of CPD formation at any specific…" – CPD/64-PP formation is also very different between the different dimers. Still in the analyses later of sequence context – either 4- or 6-mers, the analysis is for all dimers in aggregate (YY). Why not split according to each dimer to give a more accurate depiction?
2) One thing I found a bit confusing in the analysis of melanoma mutations. At the end of the day - damage enrichment drives mutagenesis – but it doesn't necessarily matter if this is due to sequence context or to bona-fide binding, correct? So it doesn't matter if damages were "enriched" by the TF over expected. Only if the site is highly damageable?
3) Can the 6-4PP damageability explain mutagenesis at sites where CPD damage/repair does not?
4) Were there no sites where TF binding improved repair? If not this is worth mentioning. In general – All the examined sites identified are bound are in accessible regions that are sites of preferred repair – so perhaps there is less dynamic range for improvement compared to reduction.
5) Why was the analysis of mutations done with a tri-nucleotide control model, and repair with tetra-nucleotide? Is it to do with the coverage?
6) For discussion: Is selective pressure relevant for mutations in TF binding sites in cancers?
7) Why was the C1SAN/CSBwt cell line chosen?

8) While damage-antibodies have known biases, are the authors sure endonucleases or photolyases used in the damage-mapping assays do not?
9) Bottom of P.6 – l. 225 – jumping to Figure 6 is a bit problematic. I suggest addressing this point later in results or in discussion

10) I may have missed it but worthwhile to mention that Y=pyrimidine in the text.
11) Typo p. 20, l.648: repair of analysis vs. analysis of repair.

(Remarks on code availability)
I have not tested the code but did check that it is well-documented and relevant scripts were made available.

Reviewer #4

(Remarks to the Author)
The manuscript by Wasserman et al. presents a comprehensive analysis of transcription factor (TF) binding and its influence on UV damage formation (CPD and 6-4PP), repair, and mutagenesis. The authors utilize a statistical framework designed to eliminate sequential bias in TF binding motifs, aiming to isolate the specific effects of protein binding from intrinsic sequence damageability. Furthermore, they employ CPD-Seq v2.0 to detect CPD adducts and their repair at single-nucleotide resolution in NHF1 cell lines following 6J/m2 UV exposure. A key proposal of the study is that TF-induced DNA bending alters the susceptibility of adjacent pyrimidines to damage; the hypothesis was supported through MD simulations.
The integration of structural predictions with damage sequencing data is an interesting approach. However, I have significant concerns. I have outlined these points below for the authors' consideration.

• While the authors developed a sophisticated hexanucleotide-based Poisson model to account for sequence-driven damage bias, the use of local scaling and naked DNA as a filter may not fully eliminate all regional sequencing artifacts, such as mappability variations within the human genome,,. Although the researchers utilized naked DNA maps to 'veto' false positives, a more conservative approach might have been to use naked DNA as the primary baseline for the Poisson model itself, rather than using it only as a secondary statistical filter,,. Furthermore, while the study focused on high-quality ATAC-seq open chromatin regions to reduce noise, it remains possible that subtle differences in accessibility or mappability between different regulatory elements could still influence the observed damage peaks

• The presentation of data as aggregated CPD counts effectively highlights broad trends across transcription factor (TF) families, but it may partially mask the site-to-site heterogeneity within a binding set. While the authors addressed this by stratifying sites based on binding strength (motif quality), which demonstrated a clear dose-response effect, the aggregate signal could still be disproportionately influenced by a subset of highly reactive genomic loci,. Future analyses could benefit from revealing the full distribution of damage across individual sites to ensure the signal is a collective property of the TF's binding behavior rather than the result of a few outliers involved in other regulatory processes.

• Skin cancer mutation analysis focused on the correlation between relative CPD formation and C>T mutations. This analysis relies heavily on the no-selection assumption for cancer mutations. However, since the subject is transcription factor binding sites, some of the mutations might be advantageous for tumorogenesis. It is unclear how the authors considered this effect. Authors also find accumulation of non-C>T mutation types in neighboring TF binding sites that are differentially displaying (6-4)PPs. Here, my question concerns the selection of the mutagenesis behavior.

• What are the alternative mechanisms that cause fewer mutations at TFB2 sites? NER inhibition by TFs would cause the asymmetric effect in the other direction.

• What is the reason to use hg19 rather than GRCh38?

• The study uses 500 J/m² for UVDE-seq, which is significantly higher than the 6 J/m² used for CPD-seq. Why did the authors not utilize publicly available Damage-Seq data (typically ~20 J/m²)? Using comparable dosages would make the CPD and 6-4PP results more directly comparable. It is not clear that why the authors exposed naked DNA samples to 12 J/m² UVC more than they used for cellular exposure for CPD-Seq samples (6 J/m²).

• The manuscript states (Line 83) that this is the "first single-nucleotide resolution, high-throughput assessment of 6-4 PP lesion formation at TF binding sites in human cells, showing dramatic enhancement from binding of specific TFs". This claim appears overstated. As referenced in the main text in a different context, the HS-Damage-Seq technique also achieves single-nucleotide resolution. Furthermore, Hu et al. (2017) (https://doi.org/10.1073/pnas.170652211) previously reported that certain motifs exhibit enriched or depleted 6-4PP levels (see Hu et al., Fig. 2). The authors should revise this statement to accurately reflect the existing literature

• In the analysis of ATAC-Seq data, the authors do not mention correcting for the Tn5 transposase offset. Since Tn5 inserts adapters 9 bp apart, it is standard best practice to shift reads (+4 bp for the + strand, -5 bp for the - strand) to accurately identify the center of the open chromatin region, especially when working with TF binding motifs (see: https://academic.oup.com/nargab/article/5/2/lqad054/7188754). I recommend applying this shift to ensure accurate intersection with motifs.

• Have the authors performed the background analysis for motifs located in non-accessible regions (the ones not intersected with ATAC-Seq peaks)? If the model is robust and accessible regions are in good quality, one would not expect to see the same TF-driven enrichment patterns in these regions. This would serve as a valuable negative control.

• The authors use a hexanucleotide context for the background model (Fig. 1b), but switch to tetranucleotides for the repair simulation model (Line 328). What is the rationale for this switch?

• In Fig. 2a, there seems to be a correlation between increased and decreased sites (z-scores) with the number of binding sites. This suggests that the more sites analyzed, the more likely to “find” significant differential damage formation. A potential artifact should be addressed.

Minor comments:

As quality control graphs for ATAC-Seq data produced, please add insert size histograms for all samples as a supplementary figure (example: https://nbis-workshop-epigenomics.readthedocs.io/en/latest/content/tutorials/data-preproc/data-qc-atac.html , Fig.2).
The authors state they excluded genic regions to avoid transcriptional biases. Given that many TFs bind in promoter-proximal regions which might be transcribed, does excluding genic regions reduce the number of relevant TF binding sites analyzed? A brief note/table on the percentage of total TFBS retained after this filter would be helpful.
Please provide details on the CPD-Seq v2.0 sequencing metrics: How many mapped reads were identified, and what is the percentage of reads containing pyrimidine dimers? Plots showing dinucleotide content frequencies of both CPD and 6-4PP mapped damage reads must be added as supplementary figures.
Figure Clarifications:
o Fig. 1a: The bar plots require a more detailed explanation in the legend or main text.
o Fig. 1b: The sequence context effect increases when moving from tetranucleotide to hexanucleotide analysis. Is this attributed to the influence of adjacent pyrimidines on the immediate flanks? This should be discussed as it influences the background profile.
2. Please explicitly state in the text (around Line 120) that 'Y' denotes pyrimidines to assist a broad readership.
3. Why are there different motif clusters for the same TFs (e.g., NFKB 1/2/3 in Fig. 6a / Supplementary Table1)? In supplementary Table1, sheet number 1 “Motif Cluster to TF mapping” lines 390, 392, 394 seems redundant, for example. Authors should clarify why it is like this.
4. Why was a BH p-value cut-off of 0.05 used for CPDs, but a different cut-off (0.01) used for 6-4PPs (Line 871)? Consistent cut-offs should be used or the discrepancy justified.
5. In Figure 2a, there is a CPD depletion at the motif binding center (0/1) for SOX. In Figure 4d, 0th position is the mutation hotspot and repair is inhibited at the position 0/1. How do the authors explain this?
6. In Figure 4f, it would be beneficial to highlight positions with depleted CPD damage and enhanced repair. These "protected" sites would be expected to have lower mutation rates.
7. Regarding Line 379, can the authors definitively state this is due to poor damage recognition? Other steric factors might be involved.
8. The authors mention that XR-seq data is hard to map to damages. Please elaborate on the specific technical challenges involved.
9. Typos and Links:
o Line 203: There appears to be a typo: "FOS/JUB".
o Line 715-728: "GEOS" should be "GEO"?
o Line 799: The provided GitHub link appears to be invalid/broken.

(Remarks on code availability)

Version 1:

Reviewer comments:

Reviewer #1

(Remarks to the Author)
The authors have addressed all my comments adequately.

(Remarks on code availability)

Reviewer #2

(Remarks to the Author)
The authors have addressed my comments satisfactorily, and I now recommend the manuscript for publication.

(Remarks on code availability)

Reviewer #3

(Remarks to the Author)
The authors have thoroughly addressed all comments, and I am happy to recommend this manuscript for publication in

Nature Communications.

(Remarks on code availability)

Reviewer #4

(Remarks to the Author)
The authors have addressed most of my comments.

However, a major concern still remains to be addressed. The fact that naked DNA samples are not used for normalization is not clearly explained in the main text. I understand that deviations can be explained by other factors, but why those deviations exist needs further explanation in the manuscript. I think authors should openly discuss this problem in the main text.

(Remarks on code availability)

Open Access This Peer Review File is licensed under a Creative Commons Attribution 4.0 International License, which permits use, sharing, adaptation, distribution and reproduction in any medium or format, as long as you give appropriate credit to the original author(s) and the source, provide a link to the Creative Commons license, and indicate if changes were made.
In cases where reviewers are anonymous, credit should be given to 'Anonymous Referee' and the source.
The images or other third party material in this Peer Review File are included in the article's Creative Commons license, unless indicated otherwise in a credit line to the material. If material is not included in the article's Creative Commons license and your intended use is not permitted by statutory regulation or exceeds the permitted use, you will need to obtain permission directly from the copyright holder.
To view a copy of this license, visit https://creativecommons.org/licenses/by/4.0/

REVIEWER COMMENTS

We thank the reviewers for their positive evaluations and constructive comments and suggestions, which helped us strengthen the manuscript. Below we provide a point-by-point response to the reviewers' comments, with our responses shown in blue font. All major changes made to the original manuscript are shown in red font in the revised main text and in the revised supplementary materials document.

Reviewer #1 (Remarks to the Author):

The study by Wasserman et al provides a high-resolution analysis of how transcription factor binding modules the DNA damage and repair at different positions within and immediately adjacent to the TFBS across multiple TFs. The statistical framework implemented to quantify damage formation and repair is rigorous, and the results obtained at the base-pair resolution are novel, as compared to previous studies. Overall, the manuscript is well-written, and the results support the conclusion.

We thank the reviewer for their positive evaluation.

The following are some minor comments for improvements:

a) Fig 1a) The number of active ETS binding sites reported was 15,533. whereas in Fig C, the total ETS binding sites reported were 31,153. Please clarify.

We clarified this in the **Fig. 1** caption. The full set of putative ETS binding sites contains 31,153 sites, which were used in the stratification analysis in **Fig.1c**. But, as described in **Methods** (section "Curation of active TF binding sites"), we only used the top 50% of sites with the highest MOODS motif scores for our statistical analyses, which is why **Fig.1a** includes only 15,533 sites (please note that the number is slightly higher than 31,153 / 2 because genomic sites with the same MOODS score, i.e. ties, are included when selecting the top 50% of sites). We revised **Table S1** to include the number of sites used for each motif cluster.

b) Fig 2f) the CPD formation/enrichment signal at position -4/-3 and -3/-2, directly 5' to its CCAT-binding motif, is interesting, despite the low conserved dipyrimidine at these sites. Given that the number of binding sites of YY1 is relatively low (as compared to ETS or CTCF), how confident is the CPD prediction for those low numbers of sites?

The number of binding sites for YY1 (3,650) is indeed smaller than the number of sites for ETS or CTCF; see revised **Table S1**. Out of the 3,650 YY1 sites, 2,453 (67.2%) and 2,205 (60.4%) sites have dipyrimidines on the motif complement strand at positions -4/-3 and -3/-2, respectively. But the precise dipyrimidines occurring at these positions are not conserved, which is why the YY1 motif has low information content at these positions. We have updated the main text to clarify this point (line 212). The number of dipyrimidines at position -4/-3, on the motif-complement

strand, are: 1405 TT, 638 TC, 264 CT, and 146 CC. The number of dipyrimidines at position -3/-2, on the motif-complement strand, are: 1192 CT, 656 TT, 243 CC, and 114 TC. Counting the number of CPDs occurring at these positions (across all dipyrimidines), we obtained 143 CPDs at position -4/-3 and 157 CPDs at position -3/-2, which was sufficient to achieve high statistical significance when compared to the background models:
Benjamini-Hochberg corrected p-value=4.82×10^{-05}, z-score=5.127 for position -4/-3, and Benjamini-Hochberg corrected p-value<10^{-06}, z-score=9.879 for position -3/-2. Accordingly, we are confident in the predictions reported at these positions.

c) Why different SD value was used in Fig 1c(4SD) and Fig 2c-h (3SD)?

In **Fig. 1c,** four standard deviations was used to enhance visibility for method demonstration. We adjusted the figure to show 3 standard deviations, for consistency.

d) Fig 3a) The Pearson correlation between the projected and observed C>T mutations is unsurprising, because the sequence context is shared by CPD and mutations are similar. In other words, the observed correlation could be simply due to differences in the CPD formation/mutation frequency among the different hexanucleotide contexts (as shown in Fig. 1b).

We thank the reviewer for this comment. Indeed, we do expect CPD formation and mutation frequency to be correlated, as shown in **Fig. 3a**, regardless of whether they are driven by sequence context or TF binding, consistent with CPD formation being a primary driver of UV-linked mutagenesis (as mentioned in our manuscript). We note, however, that our analyses in **Fig. 3** and related text also highlight TF proteins and positions in their binding sites where mutation enrichment does *not* correlate with enrichment in initial CPD formation, suggesting that alternative mechanisms, such as competition with DNA repair, may be driving mutagenesis at these positions.

e) Also, it is unclear how the "Random TF" was calculated in Figure 3a. The method says, "For the Pearson correlation coefficients of projected versus actual mutation profiles shown in Fig. 3a, the distribution of randomized comparisons was generated by calculating the correlation coefficients of a projected mutation profile of actual mutation profiles of 5 randomly selected TFs. Each marker represents the average of the five comparisons.". Does the plot cover only those 5 randomly selected TFs or all TFs, and what were compared in the Pearson correlation?

For the “Random TF” violin plot in **Fig. 3a**, the Pearson correlation coefficients were generated by comparing the actual skin cancer C>T mutation profile of a given TF cluster with the projected C>T mutation profiles of 5 randomly selected TF clusters. Each marker represents the average Pearson correlation coefficient between a given TF cluster and these 5 randomized comparisons. We updated the caption for **Fig. 3** and the **Methods** section (lines 864-869) to more clearly describe this analysis and added a new supplementary figure (**Fig. S8** in the revised manuscript).

f) In Fig 4f, the observation that the mutation enrichment at positions 3 and 4 is not significantly explained by the repair reduction contradicts the observations by Poulous et al., Cell Report,

2016. Though this could be driven by the sequence context NYYN, the repair can still be further perturbed by the CTCF binding. In Figure 4h, can the All NYYN be separated by NYYN covered by any TF bound versus unbound to further support this observation?

We thank the reviewer for bringing up this point. In fact, our results agree with the observations of Poulos et al. that reduced repair at positions 3/4 in CTCF binding sites can explain the mutation enrichment at these positions. The question is what drives the reduction in repair. We argue that this reduced repair is well explained *by sequence alone*, because most of the NYYNs at these positions have a 5' G, a context with low levels of repair both in our study, based on time-course CPD-seq data (**Fig. S9, Table S7**) and in the literature (see, for example, the XR-seq-based study of Morledge-Hampton, Kalyanaraman, and Wyrick, *NAR* 2024, cited in our manuscript). The reviewer's suggestion is interesting but we don't think it would bring clarity to the analyses, for several reasons. First, separating NYYNs would reduce the CPD counts used to estimate the background rates, thus making the estimates less reliable, especially at the 6h time point, which is critical for making statistical inferences about repair. Second, separating NYYNs in TF binding sites vs. non-binding sites would be justified only if a broad range of TFs would be able to bind CPDs in a common NYYN context; given that CPDs cause severe distortions in DNA, and that different structural TF families have different rules for what DNA distortions they accept, it is very unlikely that any particular NYYN context will be bound with high affinity by a wide range of TFs. (We need to keep in mind that, in order for a TF to reduce repair in a particular context, it needs to bind that context when it is damaged.) For these reasons, as well as the fact that there is independent evidence in the literature that the particular contexts at positions 3/4 in CTCF binding sites are slowly repaired, we chose not to perform additional analyses with NYYNs in TF binding sites filtered out.

The reviewer is correct that repair at positions 3/4 could in theory be further perturbed by CTCF binding, and it is possible such an effect may become observable at repair time points other than the 6h time point analyzed here (we discuss this limitation in the Discussion section of our manuscript). However, based on our statistical analyses of repair at 6h, positions 3/4 in CTCF binding sites show only a modest z-score of 1.025, which is not statistically significant (BH-corrected p-value=0.573, **Table S3**).

Finally, we note that our analyses do support CTCF competition with repair as a major determinant of mutation enrichment at *other* positions in CTCF binding sites, consistent with prior literature from us and others (including the study of Poulos et al, as well as a previous study from our group: Sivapragasam et al. *EMBO J.* 2021, cited in our manuscript).

g) Fig 5f, in LEF1 mutation profile, could the enrichment of C>T at the position in neighboring sites be explained by potential CpG methylation on the opposite strand?

Indeed, we thank the reviewer for this insight. We added this to the Discussion section (lines 707-710).

Reviewer #2 (Remarks to the Author):

This manuscript by Wasserman et al. presents a thorough analysis of the effects of transcription factor binding on UV DNA damage in skin cancers. The authors use recently developed high resolution techniques to map DNA damage, CPD-seq v2.0 and UVDE-seq, and perform statistical modeling to identify sites where TF binding enhances or protects against DNA damage. They also nicely compare this work in cells to mutations in skin cancer patients. Additionally, they perform CPD-seq v2.0 6 hours post UV damage, and use this time course data to model how TF binding affects repair of DNA damage. They also use AlphaFold3 to model TF-bound and unbound DNA structures to suggest how TF binding can alter DNA geometry to promote damage formation. This work is well done and nicely expands on previous work characterizing how ETS and CTCF binding affects DNA damage, now extending this work to many more classes of DNA binding factors.

We thank the reviewer for their evaluation.

My major comment on this work is that the authors claim that they can confidently attribute their findings to TF binding, versus inherent sequence features of the DNA. However, there are no direct measurements of TF binding. Given that the authors are measuring the effects of hundreds of TFs, it would be unreasonable to perform ChIP-seq experiments.

Indeed, performing ChIP-seq for hundreds of TFs is a massive effort and not within the scope of this study. Instead, our goal here is to perform a large-scale screen to identify which TFs are most likely to directly influence UV damage formation and repair, and should thus be prioritized for more in depth studies in the future.

The authors did perform CPD-seq and UVDE-seq on naked DNA, and I suggest that the results from these control experiments be shown more clearly in the paper. For example, the analysis shown in Figure S2B is very helpful and should be moved to the main body. The authors claim on lines 157 and 158 that “none of the significant peaks of CPD enrichment or depletion observed in cellular data were present in naked DNA”, but they do not present this analysis beyond what is shown for ETS and CTCF in Figure S2. The authors should present similar analysis for the UVDE-seq on naked DNA to accompany Figure 5.

We thank the reviewer for this helpful suggestion. In response, we added analyses of CPD and 6-4 PP formation in TF binding sites regions in naked DNA (new **Fig. S5** and **S6** in the revised manuscript) to accompany the heatmap analysis shown in **Fig. 2a** and **5a**. We note, however, that although these analyses of naked DNA are informative, we must interpret them with great caution, especially for CPD formation. This is because CPD formation in naked DNA shows markedly different patterns compared to cellular DNA, deviating significantly from a Poisson process for several hexanucleotide sequences (new **Fig. S5a**). These deviations are one of the reasons why we chose not to use the naked CPD-seq data to derive our background model (and instead we used the cellular CPD-seq data across the entire intergenic, chromatin accessible genome). In particular, as expected based on the violations of the Poisson model, the heatmap for CPD formation in naked DNA (new **Fig. S5b**) shows what appears to be a depletion of CPDs for certain TF clusters - these are clusters where the motifs (new **Fig. S5c**) match the

hexanucleotide sequences that deviate from the Poisson model. Thus, these are not true cases of depletion of CPDs, but cases where the modeling assumptions do not hold in naked DNA .

The reason(s) why particular hexanucleotides deviate from the Poisson model in naked CPD-seq data is unclear to us, and is beyond the scope of our current study. It is possible, for example, that without the organizing forces of chromatin and DNA-binding proteins, naked DNA in solution is more susceptible to forming distinct tertiary DNA structures that influence photodimer formation. These observations present an opportunity for future work, as they may help identify sequence contexts that are especially sensitive to structural influences on CPD formation beyond those present in cellular DNA, which have yet to be characterized.

Although we cannot directly use the naked CPD-seq data to build our background model of CPD formation, we nevertheless chose to filter, from our main analyses of TF-induced damage formation, any positions where a significant enrichment or depletion is also observed in naked DNA, to reduce as much as possible the potential for false positives. We updated the main text to reference these new figures and clarify how we use naked DNA in our analyses.

Also, are there any RNA-seq data sets the authors could use to assess TF expression in this cell type? The authors state that this analysis was performed on 597 TFs across 225 clusters, but it is now clear if all 597 TFs are in fact expressed and active in these cells. Given the subtle differences between how TFs within a same family interact with DNA, it is likely that not all TFs within these classes would have the same effects. If the expression data exists, the authors could add this information to Figure 2a. If not, they should add some additional text clarifying that the TFs in these clusters are the TFs that could bind these motifs, possibly in the paragraph containing lines 214-221.

We thank the reviewer for these comments and suggestions. While some paralogous TFs do indeed have subtle differences in the way they interact with DNA, we do not expect such differences to be captured by analyses where putative TF binding sites are identified based on DNA motifs (see, for example, our prior study of paralogous TF: Shen et al, *Cell Systems* 2018, PMID: 29605182). For this reason, in the current study we chose to analyze the TFs based on clusters of proteins with similar motifs.

Still, the reviewer's question regarding the expression levels of TFs is valid. In response, we have added the relevant gene expression data to supplemental **Table S1**. Given that RNA-seq data is not available for the exact cells used in our analyses, we instead used human skin fibroblast RNA-seq data from the ENCODE consortium (ENCSR000CUH). We briefly discuss this data in the revised Methods section (subsection "Curation of active TF binding sites"; lines 794-810), and in the Discussion section (where we had previously briefly discussed TF expression levels; lines 664-673 in the revised manuscript). We have also updated the paragraph mentioned by the reviewer (lines 216-217) in the revised manuscript) to clarify that we are identifying TFs that have the *potential* to modulate CPD formation; and we added a note about this in the Methods section as well.

Briefly, the RNA-seq data showed that a majority of the 225 TF motif clusters contain at least one expressed TF: ~70% of clusters (156) contain at least one TF with TPM (transcripts per million) ≥ 1, and ~79% of clusters (177) contain at least one TF with TPM ≥ 0.5. We chose not to use TPM values to filter out TF clusters because: 1) it is unclear what gene expression level (or, more precisely, nuclear protein level) is needed for a TF to have a significant effect on UV damage formation or repair; 2) it could be that additional TFs should be assigned to these clusters in the future (based on additional motif data that is now coming out from projects such as Codebook (preprint available at PMID 39605729)); and 3) some TFs that are not expressed based on the ENCODE RNA-seq data could be expressed in the cells used to generate the CPD-seq data. Interestingly, when comparing TF motif clusters with vs. without significant effects on CPD formation, 6-4 PP formation, or CPD repair, we found that clusters with significant effects are much more likely to contain TFs with TPM ≥ 1 (one-sided Fisher's Exact Test p=4.84e-06). This suggests that the full scope of TF binding effects on UV damage formation and repair may be broader than what we observed here, and could be further expanded by examining additional cell types in which distinct sets of TFs are expressed. We added this note in the Discussion section (664-673).

Reviewer #2 (Remarks on code availability):

The README file for section 4 (analyze repair simulations) is not complete.

We thank the reviewer for their thoroughness and have updated the README for this portion of the code repository.

Reviewer #3 (Remarks to the Author):

The manuscript by Wasserman et al. describes a comprehensive analysis of the effect of TFs on DNA damage formation in human cells. There have been multiple studies addressing the effect of a limited set of TFs on UV damage formation, focusing primarily on the ETS and CTCF factors and CPD damages. Here, Wasserman et. al. applied a thorough statistical framework and curation of high-quality and high-resolution TF binding and DNA damage data to analyse a comprehensive set of TFs and identify previously un-reported effects (both positive and negative) on damage formation. Furthermore, they apply structural analysis of TF-bound DNA to explain the observed positive and negative influence on damage formation, and link their observation to melanoma mutagenesis data, showing how both damage susceptibility and repair efficiency contribute to the final mutagenic patterns.

I find the manuscript to be clearly written and have no strong critiques. I recommend acceptance to Nature Comm after addressing the following comments:

We thank the reviewer for their positive evaluation.

1) At the end of p.3: “The frequency of CPD formation at any specific…” – CPD/64-PP formation is also very different between the different dimers. Still in the analyses later of sequence context – either 4- or 6-mers, the analysis is for all dimers in aggregate (YY). Why not split according to each dimer to give a more accurate depiction?

We apologize for any confusion regarding our tetranucleotide and hexanucleotide background models. Our models use background frequencies computed for each of the 1,024 NNYYNN hexanucleotides (**Methods**), but we show this data in aggregate in some plots (**Fig. S1a,c**), for ease of visualization. In the revised manuscript, we added supplementary figure panels that further visualize the background frequencies by each dimer - please see new panels **b** and **d** in **Fig. S1**, and new panel **b** in **Fig. S9** of the revised manuscript.

2) One thing I found a bit confusing in the analysis of melanoma mutations. At the end of the day - damage enrichment drives mutagenesis – but it doesn't necessarily matter if this is due to sequence context or to bona-fide binding, correct? So it doesn't matter if damages were “enriched” by the TF over expected. Only if the site is highly damageable?

The reviewer is correct that damage enrichment (driven by either sequence context or TF binding) generally drives mutagenesis, so we expect CPD formation and mutation frequency to be correlated, as shown in **Fig. 3a**, for many TF clusters. We argue, though, that it does matter whether the high level damage is simply due to sequence context vs. increased due to TF binding. TF-driven effects are important to identify because TF binding can vary among cell types and among organisms, so the enrichment of TF-driven mutations will also vary; in contrast, effects due to sequence context will be the same across cell types and individuals.

In addition, as we mentioned above in response to reviewer #1, our analyses in **Fig. 3** and related text also highlight TF proteins and positions in their binding sites where mutation enrichment does *not* align with enrichment in initial CPD formation, suggesting that alternative mechanisms, such as competition with DNA repair, may be driving mutagenesis at those positions.

3) Can the 6-4PP damageability explain mutagenesis at sites where CPD damage/repair does not?

This is a very interesting question. Focusing on TF motif regions +/- 5bp, we found only 4 positions across 3 TF clusters where TF-induced 6-4 PP formation enhancement (BH corrected $p<0.01$; **Table S3**) coincided with C>T mutation enrichment that was not explained by TF-induced CPD damage formation enhancement or inhibition of CPD repair. Among these positions was position 1 in the RFX/3 cluster that we discuss in **Fig. 5g**. The other 3 positions are: position 7 in cluster RFX/3, position -7 in cluster ZIC/2, and position 11 in cluster ZNF143.

We expect that while the binding of some TFs can enhance 6-4 PP formation, the greater DNA distortion induced by 6-4 PP structure (relative to CPDs) may make them: 1) less suitable for TF recognition in binding sites, and 2) a more detectable and specific substrate by UV-lesion

recognizing repair proteins like UV-DBB. Together, these effects result in 6-4 PPs being more efficiently repaired and less potentially mutagenic, which is consistent with prior studies of global genomic nucleotide excision repair in UV-irradiated cells (You et al., 2001; Young et al., 1996; Moser et al., 2005; Osakabe et al., 2015 - cited in our manuscript, see lines 696-699). However, we have not yet confirmed this using time-course UVDE-seq data, as 6-4 PPs are less abundant than CPDs and require irradiation at high UV doses to yield sufficient signal for analysis. Such high doses are cytotoxic, limiting our ability to do time-course UVDE-seq experiments at this time.

We note, however, that even in the absence of a mutation peak directly coinciding with 6-4 PP formation enrichment, the presence of this damage may still have negative effects on the cell, such as altering gene expression, disrupting DNA replication, or producing structural variants, which are outside the scope of this study but will be interesting to investigate in the future.

4) Were there no sites where TF binding improved repair? If not this is worth mentioning. In general – All the examined sites identified as bound are in accessible regions that are sites of preferred repair – so perhaps there is less dynamic range for improvement compared to reduction.

While enhanced repair was not a focus of our study in the initial submission, we agree that it is worthwhile to investigate and discuss - please see new supplemental **Fig. S10** and the corresponding text in the Results section (lines 349-351) in the revised manuscript. Briefly, for each CPD repair simulation test, in our revised study we calculated two one-sided empirical p-values to assess both repair depletion and enhancement. We identified 5 TF clusters that each have one binding site position with significantly enhanced repair. While the mechanism(s) for such an effect will need to be investigated in future studies, we do report these results in our revised manuscript.

5) Why was the analysis of mutations done with a tri-nucleotide control model, and repair with tetra-nucleotide? Is it to do with the coverage?

Indeed, the choices of how wide of a context to use when deriving various background models was related to coverage. For modeling damage formation, the CPD-seq and UVDE-seq data had enough counts at t=0h to derive hexanucleotides models (i.e. we modeled 2-bp on each side of the damageable dimers). When modeling CPD repair, we used a tetranucleotide-based model (i.e. only 1-bp on each side of the damageable dimers) because the number of CPD occurrences of certain hexanucleotides was too low at t=6h. For DNA mutations, we also used 1-bp on each side of the C>T mutations, due to coverage concerns during our simulation studies.

6) For discussion: Is selective pressure relevant for mutations in TF binding sites in cancers?

This is an important and nuanced question. At a broad level, previous studies have found that the pattern of hypermutation at TF binding sites in cancer is not explained by selection (e.g., Kaiser et al. PLOS Genetics 2016, PMID 27490693). In our current study, given that we analyzed tens of thousands of sites for hundreds of TFs, it is unlikely that selective pressure underlies the global

trends we observe. However, we cannot rule out the possibility that a small subset of mutations within TF binding sites is subject to positive selection in cancer cells.

In order to identify such driver regulatory mutations, though, one first needs to build accurate predictive models of which precise mutations in TF binding sites can vs. cannot be enriched due to TF binding; any mutations enriched beyond a background model that includes the contribution of TF binding to mutagenesis would be candidates for further studies into their functional consequences and selection in cancer cells. In this context, our current study provides important information for prioritizing TFs for further mechanistic characterization to build such background models of TF-associated mutagenesis. We added a note about this in the revised manuscript, in the Discussion section (lines 728-733).

7) Why was the C1SAN/CSBwt cell line chosen?

The CPD-seq v2.0 data from the CS1AN/CSB^{WT} cell line was leveraged from our previously published work (Duan et al., PNAS 2024). Originally isolated from a Cockyane syndrome patient, with DNA repair deficiency complemented by stably expressing the wild-type CSB gene, this cell line is well-established, easy to grow, and has been frequently used as a model to study nucleotide excision repair of UV damage.

8) While damage-antibodies have known biases, are the authors sure endonucleases or photolyases used in the damage-mapping assays do not?

The reviewer brings up an excellent point. While the bias of anti-damage antibodies is well known (e.g., CC CPDs are poorly recognized by commonly used anti-CPD antibodies; Mori et al., Photochemistry and Photobiology 1991), the DNA sequence preferences of the endonucleases and photolyases used in the CPD-seq and UVDE-seq workflows have not been characterized to our knowledge. It is in fact very possible that there are biases in the activity of these enzymes. However, by directly converting damage into detectable, precise cleavage events, these enzymatic approaches provide a more direct representation of lesion positions. As such, we believe the CPD-seq and UVDE-seq methods remain the most direct and quantitative methods currently available for genome-wide mapping of UV-induced DNA damage. Importantly, because we train our background models on a large and diverse set of genomic regions (all intergenic, chromatin accessible regions of the human genome), we expect that the potential sequence biases inherent to the enzymes used in CPD-seq and UVDE-seq are captured by our background models, thus allowing us to isolate effects of TF binding on UV damage formation and repair.

9) Bottom of P.6 – l. 225 – jumping to Figure 6 is a bit problematic. I suggest addressing this point later in results or in discussion

We agree with the reviewer’s comment and have removed this figure reference to avoid confusion.

10) I may have missed it but worthwhile to mention that Y=pyrimidine in the text.

We agree with the reviewer and have added that further clarification in the text (line 118).

11) Typo p. 20, l.648: repair of analysis vs. analysis of repair.

We thank the reviewer for their thoroughness. We updated the text accordingly.

Reviewer #3 (Remarks on code availability):

I have not tested the code but did check that it is well-documented and relevant scripts were made available.

Reviewer #4 (Remarks to the Author):

The manuscript by Wasserman et al. presents a comprehensive analysis of transcription factor (TF) binding and its influence on UV damage formation (CPD and 6-4PP), repair, and mutagenesis. The authors utilize a statistical framework designed to eliminate sequential bias in TF binding motifs, aiming to isolate the specific effects of protein binding from intrinsic sequence damageability. Furthermore, they employ CPD-Seq v2.0 to detect CPD adducts and their repair at single-nucleotide resolution in NHF1 cell lines following 6J/m2 UV exposure. A key proposal of the study is that TF-induced DNA bending alters the susceptibility of adjacent pyrimidines to damage; the hypothesis was supported through MD simulations.
The integration of structural predictions with damage sequencing data is an interesting approach. However, I have significant concerns. I have outlined these points below for the authors' consideration.

We thank the reviewer for their thorough and constructive evaluation.

• While the authors developed a sophisticated hexanucleotide-based Poisson model to account for sequence-driven damage bias, the use of local scaling and naked DNA as a filter may not fully eliminate all regional sequencing artifacts, such as mappability variations within the human genome. Although the researchers utilized naked DNA maps to 'veto' false positives, a more conservative approach might have been to use naked DNA as the primary baseline for the Poisson model itself, rather than using it only as a secondary statistical filter.

We agree with the reviewer that naked DNA is an intuitive control for inherent damageability of different DNA sequences, and in the revised manuscript we added analyses and discussion of CPD and 6-4 PP formation in TF binding site regions in naked DNA (new **Fig. S5** and **S6** in the revised manuscript) to accompany the analyses of cellular DNA in **Fig. 2a** and **5a**. Please see our detailed response to reviewer #2 above.

Briefly, our statistical analyses indicate that the patterns of CPD formation in naked DNA are significantly different from cellular DNA, as we observed multiple hexanucleotide sequences that deviate significantly from a Poisson process, leading to misleading patterns of enrichment/ depletion (see new **Fig. S5**). It is possible that without the organizing force of chromatin and DNA-binding proteins, naked DNA in solution is more susceptible to forming distinct tertiary DNA structures that influence photodimer formation. These observations present an opportunity for future work, as they may help identify sequence contexts that are especially sensitive to structural influences on CPD formation beyond those present in cellular DNA in vivo that have yet to be characterized.

Thus, while these analyses of naked DNA are informative, we interpret them with great caution and avoid using them to directly normalize CPD damage in cellular DNA as a way to isolate TF binding effects. As the reviewer noted, however, we do still use the naked DNA as an additional filter, by excluding significant results in the cellular analysis that were also present at the same position in the naked DNA condition. We use this conservative approach in order to focus on the highest-confidence results.

Furthermore, while the study focused on high-quality ATAC-seq open chromatin regions to reduce noise, it remains possible that subtle differences in accessibility or mappability between different regulatory elements could still influence the observed damage peaks

We agree with the reviewer that there are likely subtle differences in chromatin accessibility and/or mappability between regulatory elements. There could also be additional cellular factors (such as the overall epigenomic context, or the local transcriptional activity) that also modulate DNA damage formation and repair at regulatory regions. We note, however, that such factors act at scales larger than 10-20bp, which is the typical length of TF binding sites. To account for these larger-scale variations, for each TF cluster we scale the predicted CPD signal across the binding site region to the DNA immediately flanking the TF binding site, as described in the "Poisson modelling of CPD formation at TF binding sites" **Methods** section. This normalization allows us to isolate effects specifically due to TF binding, as opposed to other contributing local factors like chromatin accessibility. Scaling factors are included in **Table S3** as part of our analysis results. As expected, the scaling factors are modest and centered around ~1, as shown in the strip plots below:

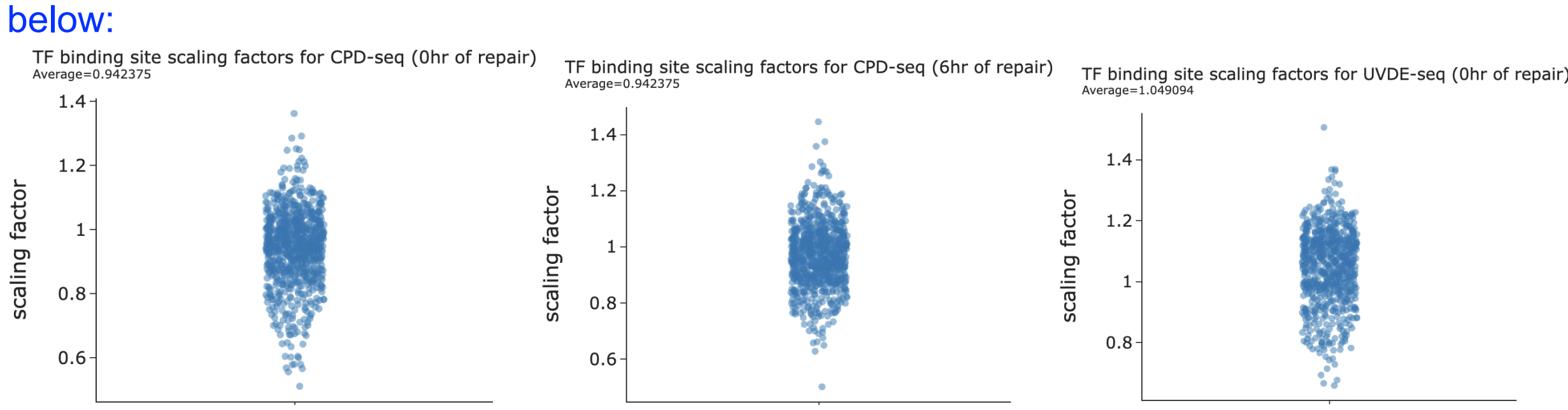

• The presentation of data as aggregated CPD counts effectively highlights broad trends across transcription factor (TF) families, but it may partially mask the site-to-site heterogeneity within a binding set. While the authors addressed this by stratifying sites based on binding strength (motif

quality), which demonstrated a clear dose-response effect, the aggregate signal could still be disproportionately influenced by a subset of highly reactive genomic loci,. Future analyses could benefit from revealing the full distribution of damage across individual sites to ensure the signal is a collective property of the TF's binding behavior rather than the result of a few outliers involved in other regulatory processes.

[REDACTED]

• Skin cancer mutation analysis focused on the correlation between relative CPD formation and C>T mutations. This analysis relies heavily on the no-selection assumption for cancer mutations.

However, since the subject is transcription factor binding sites, some of the mutations might be advantageous for tumorogenesis. It is unclear how the authors considered this effect.

As discussed above in response to reviewer #3, given that we analyzed tens of thousands of sites for hundreds of TFs, it is unlikely that selective pressure underlies the global trends we observe. However, we cannot rule out the possibility that a small subset of mutations within TF binding sites is subject to positive selection in cancer cells. Such mutations, though, cannot be identified without extensive further work to first determine, for each TF, which mutations can versus cannot be enriched due to TF binding. Only mutations enriched beyond a background model that includes the contribution of TF binding to mutagenesis (a model that is not currently available for any TF, and would require substantial future work to build) would be candidates for potential selection in the cancer cells. We added a note about this in the revised **Discussion** section.

Authors also find accumulation of non-C>T mutation types in neighboring TF binding sites that are differentially displaying (6-4)PPs. Here, my question concerns the selection of the mutagenesis behavior.

We assume the reviewer is referring here to our Discussion section, where we mention "When we examined base substitution mutations beyond C>T, we found instances of general SBS enrichment that occurs in the vicinity of strong UV damage hotspots (e.g. NFY, **Fig. S12**)." We provide a discussion of a potential mechanism that would explain this trend, but deciphering such mutagenic mechanisms is outside the scope of our current study, which focuses on damages and mutations within the TF binding sites and not their neighboring regions.

• What are the alternative mechanisms that cause fewer mutations at TFB2 sites? NER inhibition by TFs would cause the asymmetric effect in the other direction.

It is unclear which TF the reviewer is referring to, as there is no "TFB2" protein in our study. More broadly, though, the reviewer poses an interesting question about molecular mechanisms that could lead to a depletion of mutations in TF binding sites. The main mechanism we discuss in our current study is TF-induced inhibition of UV damage formation, linked to structural DNA distortions that make the UV lesions less likely to form. In the revised manuscript we also include a new analysis on enhanced repair within TF binding sites (see new **Fig. S10**), but we find relatively little support for this potential mechanism.

• What is the reason to use hg19 rather than GRCh38?

We used the hg19 reference genome in our analyses because the mutation data utilized in this study is already mapped to the hg19 coordinate system. Converting this data to GRCh38 would require re-aligning and re-annotating the mutation calls, which could possibly introduce errors and discrepancies with the original mutation data.

• The study uses 500 J/m² for UVDE-seq, which is significantly higher than the 6 J/m² used for CPD-seq. Why did the authors not utilize publicly available Damage-Seq data (typically ~20 J/m²)? Using comparable dosages would make the CPD and 6-4PP results more directly comparable. It is not clear that why the authors exposed naked DNA samples to 12 J/m² UVC more than they used for cellular exposure for CPD-Seq samples (6 J/m²).

The specific dosage for the various CPD-seq and UVDE-seq experiments was decided based on several factors. First, we need the dosage to be high enough so that we obtain sufficient damage counts for modeling and statistical analyses; given that 6-4PPs are much less frequent than CPDs, it is necessary to use a higher UV dose to quantify 6-4PP damages (by UVDE-seq) than CPD damages (by CPD-seq). Second, in order to observe the reduction of damage over time in cells, we need the dosage to be low enough to limit cell death and allow enough time for repair, while still observing sufficient damages at a later timepoint (here, 6h). The dosages for CPD-seq have been optimized in our previous study (Duan et al., PNAS 2024). Importantly, the fact that different doses are used in cellular DNA (compared to naked DNA) is not a problem for our study because our background models are derived from cellular DNA CPD-seq (as discussed in detail in our responses above), and thus inherently control for dosage specific effects.

• The manuscript states (Line 83) that this is the "first single-nucleotide resolution, high-throughput assessment of 6-4 PP lesion formation at TF binding sites in human cells, showing dramatic enhancement from binding of specific TFs". This claim appears overstated. As referenced in the main text in a different context, the HS-Damage-Seq technique also achieves single-nucleotide resolution. Furthermore, Hu et al. (2017) (https://doi.org/10.1073/pnas.170652211) previously reported that certain motifs exhibit enriched or depleted 6-4PP levels (see Hu et al., Fig. 2). The authors should revise this statement to accurately reflect the existing literature

We thank the reviewer for their comment and for highlighting the important work by Hu et al., which we reference throughout our manuscript. We revised the text referenced above to read "Additionally, we report the first **large-scale**, single-nucleotide resolution assessment of 6-4 PP lesion formation at TF binding sites in human cells, showing dramatic enhancement from binding of specific TFs, **significantly above the level of 6-4 PP formation expected based on DNA sequence alone.**", which is a clearer statement about our contribution. We note that our statement is not about the resolution of the HS-Damage-Seq data (which is indeed single-nucleotide). We also note that a direct comparison of HS-Damage-Seq data vs. CPD-seq/UVDE-seq data is beyond the scope of our current study. Instead, our statement is about the large scale of our statistical analyses for assessing enrichment of 6-4PP lesions at TF binding sites. Hu et al (2017) did indeed analyze 19 TFs and compared the 6-4PP patterns (as measured by HS-Damage-Seq) at their binding sites, between cellular DNA and naked DNA. But their analysis was limited to only 19 TFs, and no statistical quantification was provided to assess the significance of the reported trends.

We also note that in our Results section "6-4 PP formation is dramatically enhanced by transcription factor binding", we acknowledged that 19 of the TFs analyzed in our study had

been previously examined for their impact on 6-4PP formation (lines 426-429) in the revised manuscript), and we cite the study of Hu et al. For these 19 TFs, it would have been very interesting to compare the results of Hu et al. (based on HS-Damage-Seq) against our results (based on UVDE-seq); unfortunately the exact positions of observed 6-4PP enrichment/depletion were not reported by Hu et al., so we could not perform a full comparison. Lastly, we did compare the results of Hu et al. for specific TFs (e.g., NFY, which was discussed in their manuscript) to what we find in our study.

• In the analysis of ATAC-Seq data, the authors do not mention correcting for the Tn5 transposase offset. Since Tn5 inserts adapters 9 bp apart, it is standard best practice to shift reads (+4 bp for the + strand, -5 bp for the - strand) to accurately identify the center of the open chromatin region, especially when working with TF binding motifs (see: https://academic.oup.com/nargab/article/5/2/lqad054/7188754). I recommend applying this shift to ensure accurate intersection with motifs.

We agree with the reviewer on the importance of correcting for Tn5 transposase offset when processing ATAC-seq data. We used the official ENCODE atac-seq pipeline (https://github.com/encode-dcc/atac-seq-pipeline?tab=readme-ov-file) for processing our ATAC-seq data, which automatically corrects for this offset in its workflow (see page 18 of the ATAC-seq pipeline specifications: https://docs.google.com/document/d/1f0Cm4vRyDQDu0bMehHD7P7KOMxTOP-HiNoIvL1VcBt8/edit?tab=t.0). We have included a note in our Methods section clarifying that the ENCODE pipeline includes this correction (lines 757).

• Have the authors performed the background analysis for motifs located in non-accessible regions (the ones not intersected with ATAC-Seq peaks)? If the model is robust and accessible regions are in good quality, one would not expect to see the same TF-driven enrichment patterns in these regions. This would serve as a valuable negative control.

We thank the reviewer for this thoughtful suggestion. We agree that examining patterns in non-accessible regions is an interesting direction. However, we believe a rigorous analysis of nucleosomal DNA falls outside the scope of the current study for several reasons. First, our background models were trained specifically on accessible chromatin, so any patterns they might detect in nucleosomal regions could simply be artifacts of applying the models to sequences that are very different from the ones they were trained on. There would be no principled way to tell apart such artifacts from potentially true biological signals, given the mismatch between the background models and the types of genomic regions analyzed (i.e. nucleosomal DNA). Second, a growing body of work has shown that certain pioneer factors and other TFs bind nucleosomal DNA in biologically meaningful ways, so a proper analysis would require retraining background models appropriate to these regions and carefully distinguishing genuine nucleosomal binding from noise. This represents a substantial body of work that we believe is better suited for a dedicated follow-up study, and we have a sentence in the Discussion noting this as an important future direction.

• The authors use a hexanucleotide context for the background model (Fig. 1b), but switch to tetranucleotides for the repair simulation model (Line 328). What is the rationale for this switch?

The rationale for this switch is coverage. After 6h of repair time, in which ~62% of CPDs were repaired, the data became sparse at specific NNYYNN hexanucleotides, with 9 out of 1024 possible NNYYNNs not having any detectable DNA damage at 6h. The low counts for certain hexanucleotides precluded estimations of repair for robust model development, which is why we used tetranucleotide-based models instead.

• In Fig. 2a, there seems to be a correlation between increased and decreased sites (z-scores) with the number of binding sites. This suggests that the more sites analyzed, the more likely to "find" significant differential damage formation. A potential artifact should be addressed.

We respectfully disagree with the reviewer on this point. First, we do see clusters with large numbers of binding sites but low z-scores, and vice-versa. Second, having more binding sites (and more CPDs in the binding sites) gives us more power in our statistical tests, enabling higher sensitivity (i.e. identifying significant signals even when they are of lower magnitude). But this is not an artifact. Also, we note that the background level of damage for a cluster is computed by applying the background model to all the sites in that cluster and aggregating the expected damage counts; thus, the size of the cluster is automatically corrected for.

Minor comments:

As quality control graphs for ATAC-Seq data produced, please add insert size histograms for all samples as a supplementary figure (example: https://nbis-workshop-epigenomics.readthedocs.io/en/latest/content/tutorials/data-preproc/data-qc-atac.html , Fig.2).

We thank the reviewer for their helpful suggestion. Insert size histograms for the ATAC-seq samples were added to supplementary **Table S6**.

The authors state they excluded genic regions to avoid transcriptional biases. Given that many TFs bind in promoter-proximal regions which might be transcribed, does excluding genic regions reduce the number of relevant TF binding sites analyzed? A brief note/table on the percentage of total TFBS retained after this filter would be helpful.

The reviewer is correct that to avoid potential confounders related to transcription in open chromatin regions (e.g. transcription-coupled NER or higher incidence of single-strandedness), we excluded TF binding sites in transcribed regions from our analysis. There were in total 3,693,180 chromatin accessible binding sites for the 225 TF clusters in our study, of which 2,329,615 (63.0%) were located in intergenic regions and considered for our analysis. Chromatin accessible regions were determined from the ATAC-seq data described in the **Methods** section. We included this detail in our revised **Methods** section (lines 775-779), and we additionally report the number of binding sites used per TF cluster in revised supplementary **Table S1**.

Please provide details on the CPD-Seq v2.0 sequencing metrics: How many mapped reads were identified, and what is the percentage of reads containing pyrimidine dimers? Plots showing dinucleotide content frequencies of both CPD and 6-4PP mapped damage reads must be added as supplementary figures.

We thank the reviewer for their helpful suggestion. The CPD-seq v2.0 data used in the study was leveraged from our prior study (Duan et al., PNAS 2024). Please see Results section "CPD-seq 2.0 for Genome-Wide UV Damage Mapping in Human Cells" and Figure 2b of our prior paper (screenshot below) for details on the number of mapped reads and distribution of reads across dinucleotide sequences for these CPD-seq v2.0 experiments.

[REDACTED]

We have added a similar figure for the new UVDE-seq data generated in this study, as new **Fig. S15**.

Figure Clarifications:
o Fig. 1a: The bar plots require a more detailed explanation in the legend or main text.

We apologize for the confusion, the bar plots the reviewer is referencing are histograms of CPD count per genomic locus. We have updated the figure to explicitly say that these plots are histograms, and we clarified this in the figure as well.

o Fig. 1b: The sequence context effect increases when moving from tetranucleotide to hexanucleotide analysis. Is this attributed to the influence of adjacent pyrimidines on the immediate flanks? This should be discussed as it influences the background profile.

When moving from tetranucleotide (NYYN) to hexanucleotide (NNYYNN) sequences, we do increase the size of the context, but not the magnitude of the effect. We interpret the reviewer's comment as a possible misunderstanding regarding the difference between increased *variance* and increased *effect*. This may have arisen from the fact that our initial plots of CPD formation fraction at tetranucleotides and hexanucleotides are averaged over many occurrences across the genome for each unique NYYN and NNYYNN sequence, respectively. For tetranucleotide plots, the averages for each unique NYYN convolute extended sequence context effects in contrast to the hexanucleotide plots. Thus the *variance* between tetranucleotides may have appeared to be smaller. To clarify this part of our work, and also in response to a comment from reviewer #3, we added supplementary figure panels that further visualize the background frequencies by each dimer (new panels **b** and **d** in **Fig. S1**). We also note that, according to our analyses as well as

prior studies using orthogonal, gel-based analyses (Lu et al 2021, NAR; cited in our manuscript), additional pyrimidines in the flanks of YYs do often increase damageability, but the trends are more complex than this and they are explicitly modeled in our statistical framework.

2. Please explicitly state in the text (around Line 120) that 'Y' denotes pyrimidines to assist a broad readership.

We agree with the reviewer's suggestion and updated the text (lines 118) to include this clarification.

3. Why are there different motif clusters for the same TFs (e.g., NFKB 1/2/3 in Fig. 6a / Supplementary Table1)? In supplementary Table1, sheet number 1 "Motif Cluster to TF mapping" lines 390, 392, 394 seems redundant, for example. Authors should clarify why it is like this.

We thank the reviewer for raising this point. The TF motif clusters used throughout our study come from Vierstra et al. (2020), who unified thousands of TF binding motif models from major databases (JASPAR, HOCOMOCO, CisBP, UniProbe) into a non-redundant set of motif archetypes clustered by similarity (see figure below from Vierstra et al.). They then scanned the entire genome with these archetypes to produce genome-wide, cell-type-agnostic binding site calls, which are the calls we use here. We chose this resource because it is comprehensive, non-redundant, and leverages the full body of publicly available TF motif data in a single consistent framework.

Importantly, while cluster names generally reflect the associated TF family, a single TF or family can map to multiple clusters (as with NFKB1/2/3). This occurs because different motif models for the same TF family can differ enough in sequence specificity to be partitioned into separate groups during hierarchical clustering, reflecting related but non-identical binding preferences—for example, distinct binding modes of the same factor (see Badis et al., Science 2009, PMID 19443739, for a discussion of TFs with multiple binding modes captured by distinct motifs).

[REDACTED]

Figure from Vierstra et al., 2020

4. Why was a BH p-value cut-off of 0.05 used for CPDs, but a different cut-off (0.01) used for 6-4PPs (Line 871)? Consistent cut-offs should be used or the discrepancy justified.

The different thresholds reflect a deliberate choice driven by the data characteristics of each assay. UVDE-seq requires a higher UV dosage than CPD-seq in order to generate sufficient 6-4 PP damage signal, and this results in higher background noise, as we note in the caption of **Fig. 5** and in **Methods** (lines 925-928). Given this noisiness, we applied a more stringent BH threshold ($p < 0.01$) to the 6-4 PP analyses to be more conservative in calling significant results than we were for the CPD-seq data. We have clarified this rationale in the **Methods** section. All p-values and z-scores for both analyses are provided in **Table S3** so that readers can apply alternative thresholds if desired.

5. In Figure 2a, there is a CPD depletion at the motif binding center (0/1) for SOX. In Figure 4d, 0th position is the mutation hotspot and repair is inhibited at the position 0/1. How do the authors explain this?

We thank the reviewer for raising this apparent discrepancy. The CPD depletion shown in **Fig. 2a** is measured relative to the damage expected at these positions in the absence of SOX binding. The fact that we see this depletion indicates that SOX binding reduces CPD formation at the 0/1 position compared to the expected background level at these sequences. However, the absolute level of CPD damage at this position remains high, because the 0/1 dinucleotide is an intrinsically favorable substrate for CPD formation. Enough damage accumulates there to enable measurement of repair, and our time-course data analyses show that repair of CPD damage is significantly attenuated by the bound TF, as shown in **Fig. 4d**.

6. In Figure 4f, it would be beneficial to highlight positions with depleted CPD damage and enhanced repair. These "protected" sites would be expected to have lower mutation rates.

In the revised manuscript we added an analysis of enhancement of repair (please see new **Fig. S10**). We note, however, that we found no binding site positions, for any of the clusters analyzed, that showed both depleted CPD damage and enhanced repair.

7. Regarding Line 379, can the authors definitively state this is due to poor damage recognition? Other steric factors might be involved.

We agree with the reviewer that other steric factors may contribute, and we cannot definitively exclude all alternative mechanisms. That being said, we favor poor CPD recognition as the most likely explanation, for several reasons. First, the hexanucleotides at this position are poorly repaired in general (i.e. not just within CTCF sites), which points to an intrinsic property of damage recognition rather than a CTCF-specific steric effect. Second, this interpretation is consistent with prior work based on independent data (e.g., Morledge-Hampton, Kalyanaraman, and Wyrick, NAR 2024, cited in our manuscript). Still, we agree that these observations do not rule out contributions from other mechanisms, and we discuss several alternatives in the Discussion (lines 702–719).

8. The authors mention that XR-seq data is hard to map to damages. Please elaborate on the specific technical challenges involved.

We are happy to elaborate. Two features of XR-seq make it less suited to the analyses in our study, compared to CPD-seq/UVDE-seq. First, XR-seq captures damages that are actively being repaired at a given timepoint, providing a snapshot of repair activity at that timepoint rather than a complete picture of genome-wide damage. Damages not being actively repaired at the moment of sampling are missed. Second, XR-seq reads correspond to the ~30-nt fragments excised during repair. These fragments are mapped to the genome at single-nucleotide resolution, so repair activity is localized precisely, but the position of the lesion itself within each excised fragment can vary, which complicates mapping damage locations at single-nucleotide resolution. CPD-seq and UVDE-seq, by contrast, directly map the damage sites themselves. For further technical details on lesion mapping within excised fragments in XR-seq, we refer the reviewer to Hu et al., Genes & Development 2015 (PMID 25934506); Adar et al., PNAS 2016 (PMID 27036006); Hu et al., PNAS 2016 (PMID 27688757); and Li et al., PNAS 2017 (PMID 28607059).

9. Typos and Links:
o Line 203: There appears to be a typo: "FOS/JUB".
o Line 715-728: "GEOS" should be "GEO"?
o Line 799: The provided GitHub link appears to be invalid/broken.

We have fixed the above typos.

REVIEWER COMMENTS

We thank the reviewers for their positive evaluations and constructive comments and suggestions, which helped us strengthen the manuscript. Below we provide a point-by-point response to the reviewers' comments, with our responses shown in blue font.

Reviewer #1 (Remarks to the Author):
The authors have addressed all my comments adequately.

Reviewer #2 (Remarks to the Author):
The authors have addressed my comments satisfactorily, and I now recommend the manuscript for publication.

Reviewer #3 (Remarks to the Author):
The authors have thoroughly addressed all comments, and I am happy to recommend this manuscript for publication in Nature Communications.

We thank the reviewers for their evaluations and their helpful feedback throughout the review process.

Reviewer #4 (Remarks to the Author):
The authors have addressed most of my comments. However, a major concern still remains to be addressed. The fact that naked DNA samples are not used for normalization is not clearly explained in the main text. I understand that deviations can be explained by other factors, but why those deviations exist needs further explanation in the manuscript. I think authors should openly discuss this problem in the main text.

We thank the reviewer for their thoughtful feedback. We agree that the observed differences between CPD formation in naked DNA vs. cellular DNA warrant further investigation. However, a mechanistic characterization of these deviations would fall outside the scope of this study which is focused on TF binding effects on UV damage formation in a cellular context. As such, we designed our analyses to model UV damage formation in vivo, using CPD-seq/UVDE-seq data from cellular DNA, not naked DNA. We explicitly discuss this rationale and the observed discrepancies between naked and cellular DNA in the main text in lines 710–713. We again thank the reviewer and hope our response addresses this remaining concern.
