# Supplementary material for: High-throughput characterization of transcription factors that modulate UV damage formation and repair at single-nucleotide resolution
Source: Nat Commun. 2026 Jul 10;17:8506. doi: 10.1038/s41467-026-75115-4 (PMC13483863; doi:10.1038/s41467-026-75115-4)
Supplement: Supplementary file 2 [file 41467_2026_75115_MOESM1_ESM.pdf]

Table of Contents

SUPPLEMENTARY FIGURES *2*

Supplementary Figure 1. The effect of sequence context on CPD and 6-4 PP formation 2

Supplementary Figure 2. CPD and 6-4 PP formation in cellular DNA adheres to a Poisson process 3

Supplementary Figure 3. CPD formation of NYYN sequences compared with literature 4

Supplementary Figure 4. Differential CPD formation in ETS and CTCF binding sites is a function of TF binding 5

Supplementary Figure 5. CPD formation in naked DNA does not follow a Poisson process 6

Supplementary Figure 6. 6-4 PP formation analysis of UVDE-seq data from naked DNA 8

Supplementary Figure 7. TF binding effects on CPD formation are consistent across analytical and simulation analysis methodologies 9

Supplementary Figure 8. Schematic detailing comparative analysis approach for CPD-projected versus actual skin cancer C>T mutations 10

Supplementary Figure 9. The effect of sequence context on CPD repair 11

Supplementary Figure 10. Additional examples of CPD repair modulation at active TF binding sites 12

Supplementary Figure 11. CPD repair modulation in active CTCF binding sites 13

Supplementary Figure 12. General single base substitution mutation enrichment in NFY binding site flanks 14

Supplementary Figure 13. Structural analysis of TF-bound dipyrimidines using AlphaFold 3 15

Supplementary Figure 14. Bootstrap modelling of CPD repair in TF binding sites using timecourse data 16

Supplementary Figure 15. UVDE-seq read counts per dinucleotide sequence 18

SUPPLEMENTARY TABLES *19*

Supplementary Table 1. Multiple linear regression of tetranucleotide sequence features on CPD repair efficiency 19

SUPPLEMENTARY METHODS AND MATERIALS *20*

Simulation of CPD repair in TF binding sites 20

Simulation of CPD formation and C>T transitions in TF binding sites 21

ATAC-seq oligos and adaptors 22

UVDE-seq oligos and adaptors 22

SUPPLEMENTARY REFERENCES *24*

SUPPLEMENTARY FIGURES

Supplementary Figure 1. The effect of sequence context on CPD and 6-4 PP formation

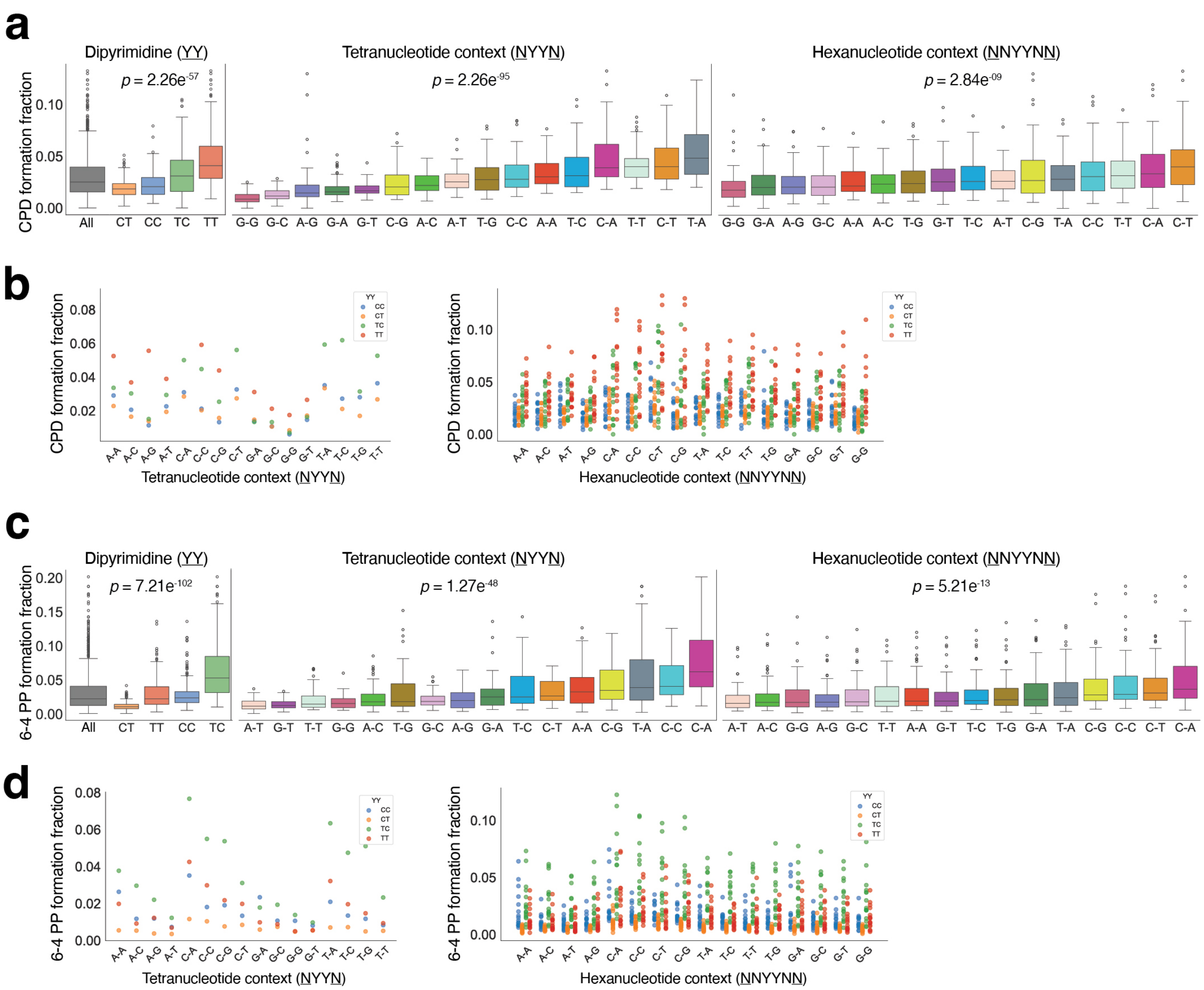

(a) Box plots of CPD formation fraction grouped by dipyrimidines (YY, *n*=4), tetranucleotide (NYYN, *n* =64), and hexanucleotide (NNYYNN, *n* =1,024) sequence context. CPD counts were aggregated from CPD-seq v2.0 data[1] of all intergenic, chromatin accessible regions in human skin fibroblasts, processed immediately after irradiation with 6J/m^2 UVC. Y-axis defined as $\mathrm{CPDcount_k}/\mathrm{count_k}$, where k is the given k-mer sequence. Included are Kurskal-Wallis test *p*-values for each sequence context grouping. **(b)** (Left) Scatter plot of CPD formation fraction per tetranucleotide (left) and hexanucleotide (right) sequence context. The color of markers correspond to the central dipyrimidine type. **(c)** As in panel a, but for 6-4 PP formation from UVDE-seq experiments of all intergenic, chromatin accessible regions in human skin fibroblasts processed immediately after irradiation with 500J/m^2 UVC. **(d)** As in panel b, but for 6-4 PP formation. Source data are provided in Supplementary Data 2.

Supplementary Figure 2. CPD and 6-4 PP formation in cellular DNA adheres to a Poisson process

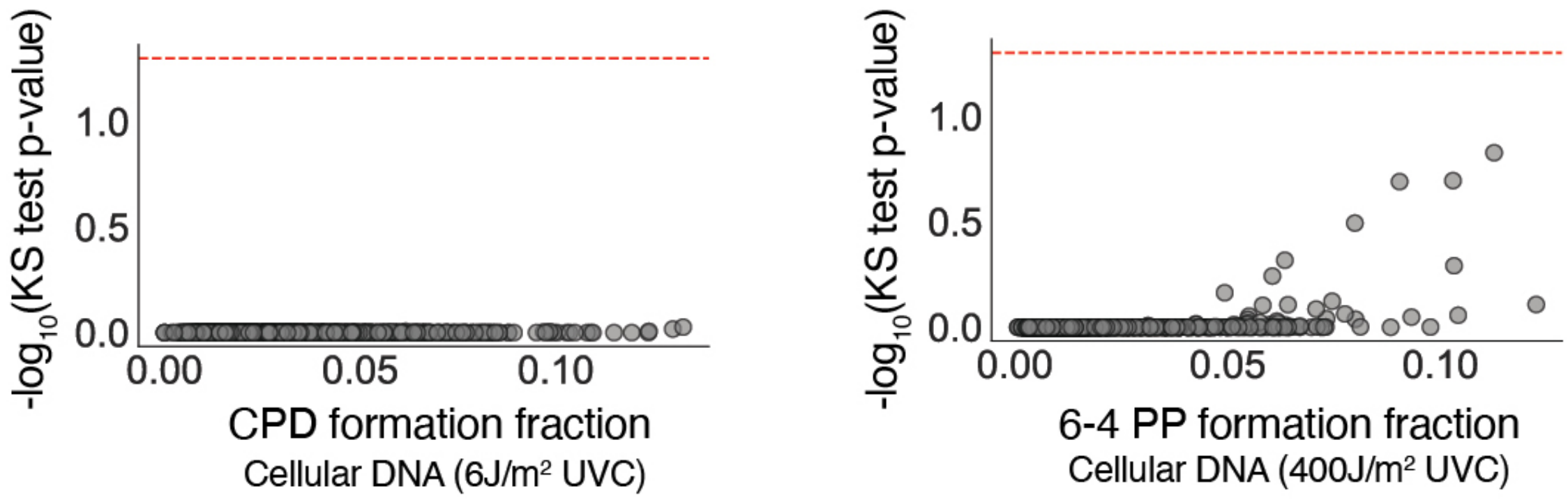

(Left) Scatter plot of $-\log_{10}$ transformed *p*-values computed from Kolmogorov-Smirnov (KS) tests comparing the theoretical Poisson and actual CPD formation count distribution for each NNYYNN sequence. Distributions were derived from CPD-seq v2.0 data in the same genomic regions described in Supplementary Figure 1a. All datapoints are beneath the red dashed line (p=0.05), indicating that the theoretical Poisson distribution does not significantly deviate from the actual CPD data per the KS test for all NNYYNNs. (Right) Equivalent plot but for 6-4 PP formation derived from UVDE-seq data in the same genomic regions described in Supplementary Figure 1c. Source data are provided in the Source Data file.

Supplementary Figure 3. CPD formation of NYYN sequences compared with literature

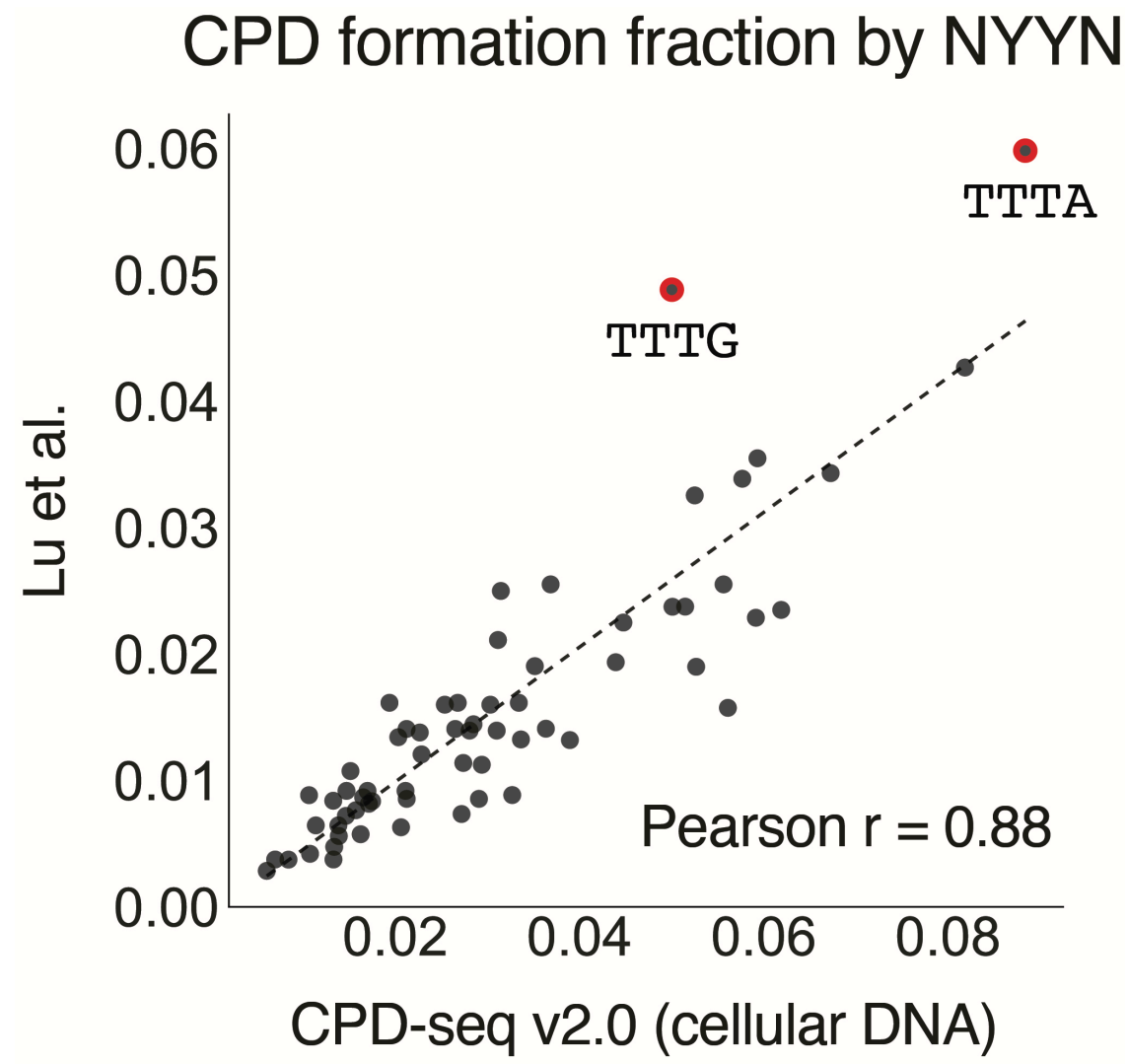

Scatter plot showing correlation of NYYN sequence CPD formation frequencies derived from CPD-seq v2.0 in vivo data published by Duan et al. (x-axis) and in vitro frequencies (y-axis) reported by Lu and colleagues.[2] CPD-seq v2.0 data measurements were calculated from intergenic, open chromatin regions in human skin fibroblasts irradiated with 6J/m^2 UVC. The dashed line is the ordinary least squares fit of the data. Overall, the two experiments are strongly correlated (Pearson r=0.88). Sequence TTTG is an outlier (OLS residual error two-sided p-value=1.3e-5), where the CPD formation frequency reported by Lu et al. is significantly discordant from the CPD-seq v2.0 derived frequencies. This discrepancy may be a reflection of the much higher UVC dosage used by Lu et al. (500J/m^2 over 8 seconds vs. just 6J/m^2 used by Duan et al.). Sustained exposure to high dosages of UVC is known to cause CPD photoreversal, especially for cytosine-containing NYYN sequences[2–4]. Thus, the formation frequency of sequences that are less susceptible to photoreversibility, like TTTG (and TTTA to a lesser extent), may appear disproportionately high due to the large UVC dosage condition used in Lu et al. Source data are provided in the Source Data file.

Supplementary Figure 4. Differential CPD formation in ETS and CTCF binding sites is a function of TF binding

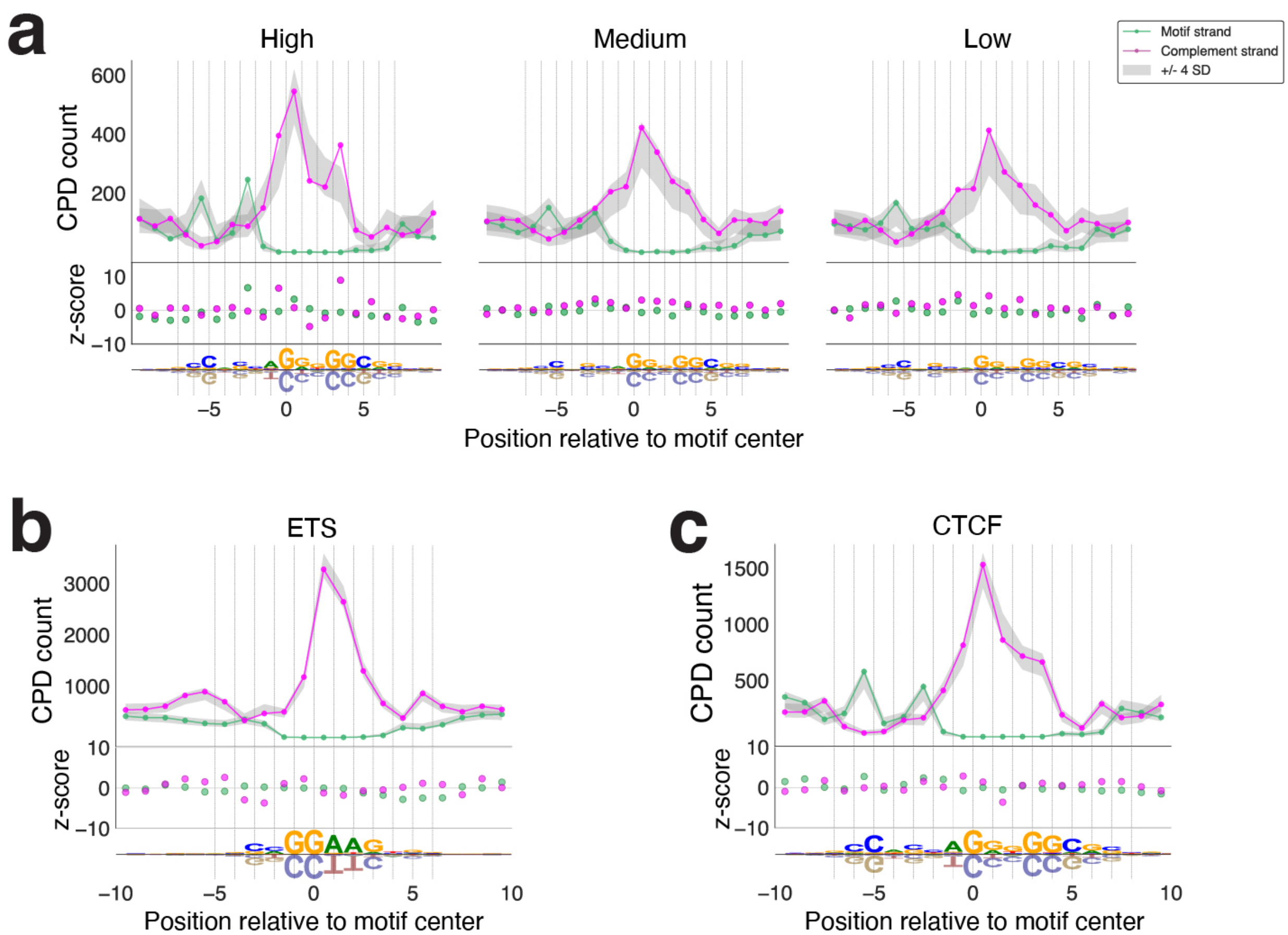

(a) Full analysis of CPD formation in all intergenic, chromatin accessible CTCF binding sites[5] (*n*=56,765), stratified by binding site strength. Binding sites were divided into equal thirds based on MOODS motif quality score[6], with higher scores indicating stronger CTCF binding. Green and pink lines CPD profile plot represent raw CPD counts per position on the CTCF motif and motif-complement strands, respectively. CPD marker positions are between nucleobase positions, indicating the photodimer location. Gray shaded region in the represents expected CPD levels ±4 standard deviations, according to the background Poisson model of CPD formation, conditioned on the cumulative hexanucleotide sequence content at each position. Included below the CPD profiles are z-scores per position, colored by strand. Sequence logos show motif content of sites used in each binding swtrength tier. X-axis denotes position relative to the binding motif center. **(b)** Similar to a, but for CPD formation analysis of high-affinity ETS binding sites (*n*=15,583) in naked DNA that was first extracted from human skin fibroblasts and purified of proteins before irradiation with 12J/m^2. See Duan et al. for details[1]. Background model was developed as described in the "Poisson modelling of CPD formation at TF binding sites" Methods section but using naked DNA CPD-seq v2.0 data. **(c)** Equivalent to b**,** but for CPD formation in high-affinity CTCF binding sites (*n*=28,384) in naked DNA. Source data are provided in Supplementary Data 3.

Supplementary Figure 5. CPD formation in naked DNA does not follow a Poisson process

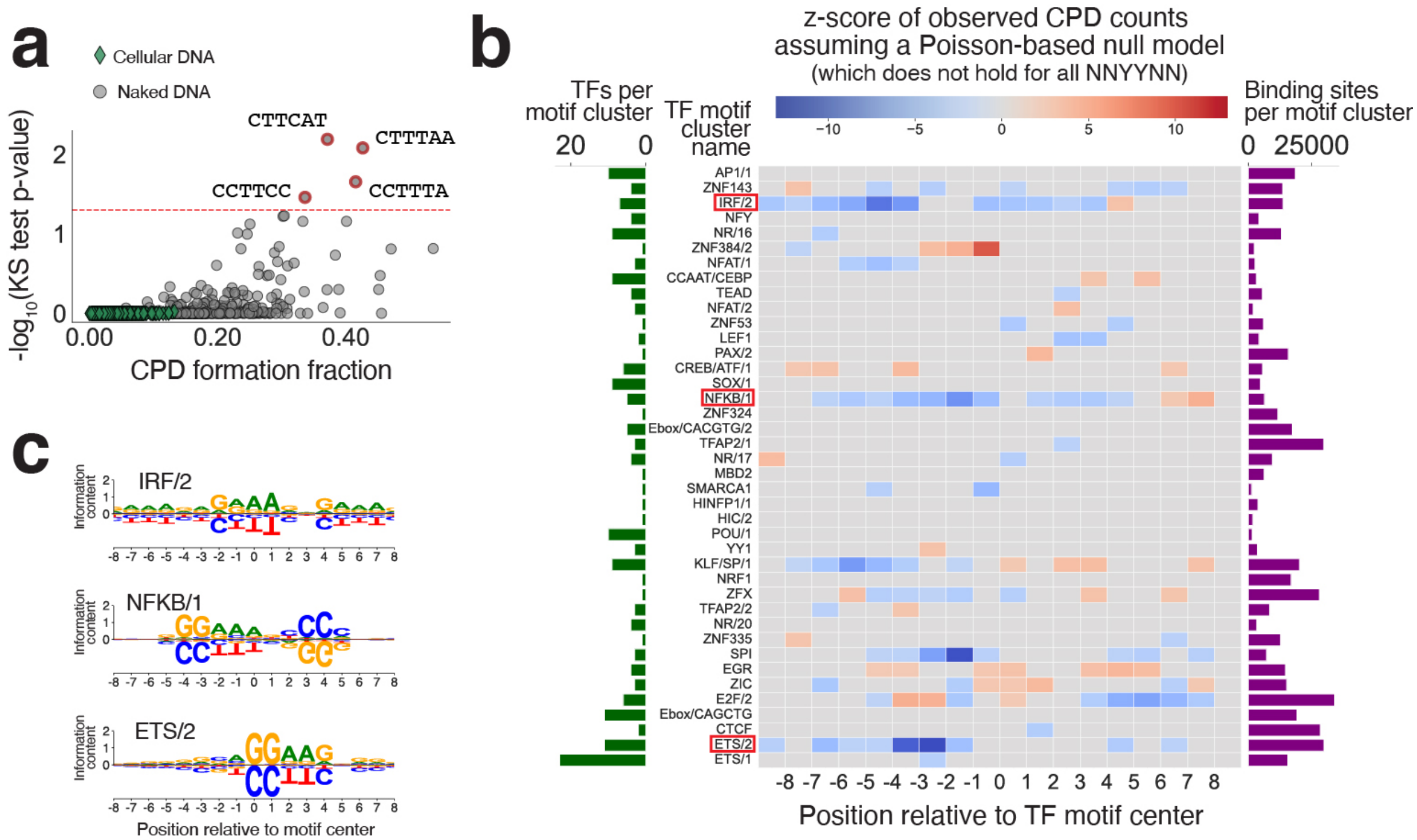

(a) Scatter plot of $-\log_{10}$ transformed *p*-values computed from Kolmogorov-Smirnov (KS) tests comparing the theoretical Poisson and actual CPD formation count distribution for each NNYYNN sequence (*n*=1,024). Gray markers are *p*-values derived from a CPD-seq v2.0 experiment on genomic DNA that was first extracted from cells and purified of proteins (i.e. naked DNA) prior to treatment with 12J/m^2 UVC (GEO accession GSE235483). Green diamond markers are the same *p*-values derived from cellular DNA shown in Supplementary Figure 2 for comparison. NNYYNN sequences that are above the red dashed line (p=0.05) and thus significantly deviate from a Poisson process, are annotated. In comparison with cellular DNA, we note that CPD damage formation of NNYYNN sequences in naked DNA condition do not adhere to a Poisson process. **(b)** Heatmap showing significant enrichment and / or depletion of CPDs across the same TF binding site regions for the 49 TF clusters shown in Figure 2a, based on a Poisson model of CPD formation conditioned on NNYYNN sequences in naked DNA (Poisson test one-sided p<0.05, Benjamini-Hochberg corrected). This model assumes that CPD formation follows a Poisson process determined by local sequence context, which, as indicated in panel a, does not adequately fit naked DNA CPD frequency, specifically. We provide this analysis to illustrate how CPD formation in cellular versus naked DNA is not necessarily analogous. Based on these findings, we discourage using naked DNA to directly normalize CPD formation in cellular DNA to isolate TF binding effects. However, to be conservative and present the highest-confidence results in our heatmap analyses of photodimer formation in cellular DNA, we use this, albeit imperfect, naked DNA analysis to spot-check for type 1 error by excluding any significant results in the cellular DNA condition that were also present at the same position in the naked DNA condition. The z-score of the observed CPD signal was determined per position, for each strand of each TF cluster. At each position, the z-score with the largest magnitude (for either the motif strand or the motif-complement strand) was used as the cell value. Each row is a specific TF cluster. The bar plots on each side of the heatmap illustrate the number of distinct TFs mapped to each TF cluster (left) and the number of binding sites attributed to each TF cluster (right). It is evident that under these model assumptions, there is considerable signal not explained by CPD damageability of naked DNA NNYYNN sequence alone. **(c)**

PWM sequence logos for representative binding sites highlighted in panel b that exhibit pronounced deviations from the Poisson-based sequence model. These regions show substantial unexplained signal under the assumption that CPD formation in naked DNA follows a Poisson process. Notably, these poorly predicted sites are enriched for extended C/T pyrimidine tracts (motif complement strands), similar to the NNYYNN contexts identified in panel a that deviate from a Poisson process. Source data are provided in Supplementary Data 3.

Supplementary Figure 6. 6-4 PP formation analysis of UVDE-seq data from naked DNA

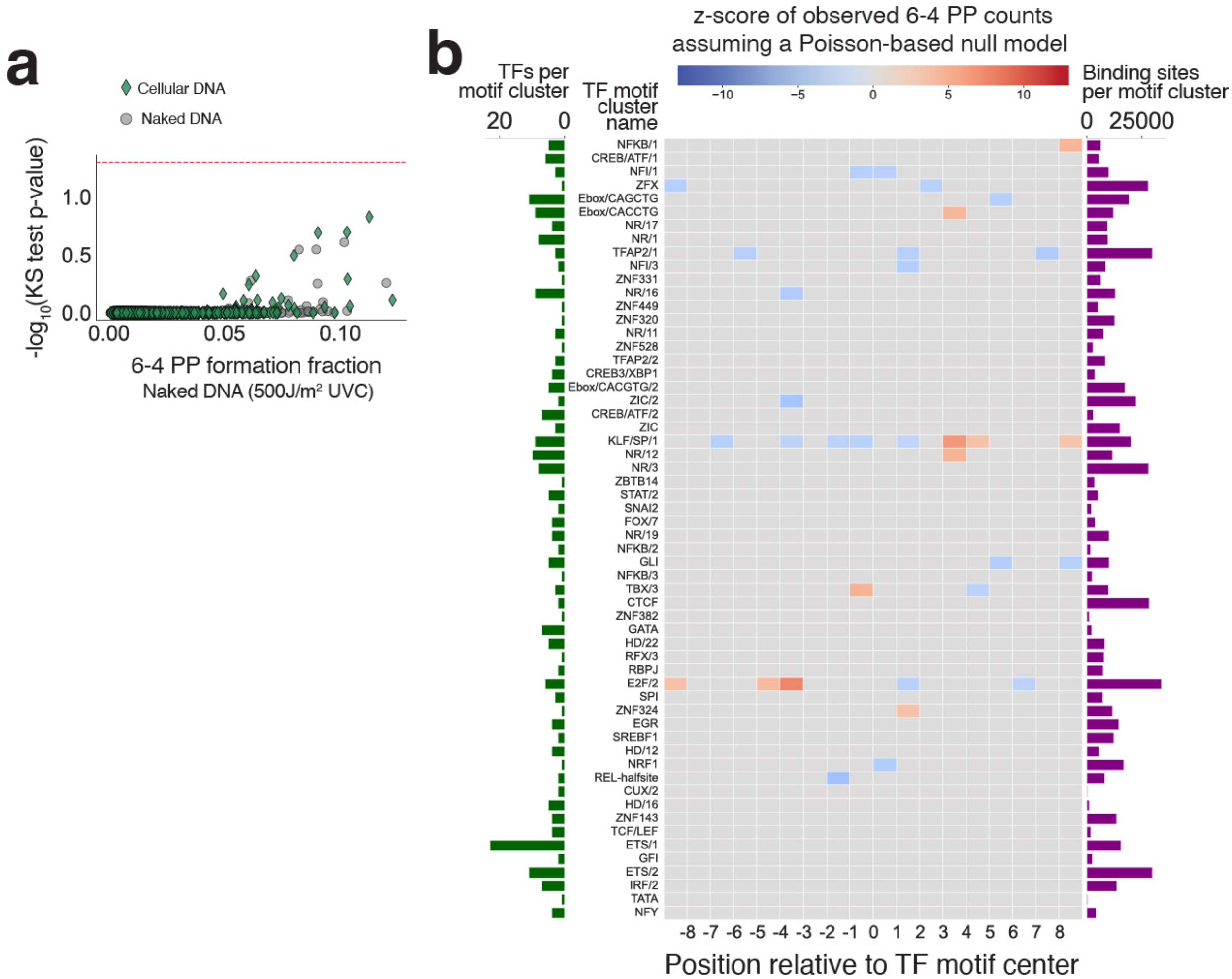

(a) Scatter plot of $-\log_{10}$ transformed *p*-values computed from Kolmogorov-Smirnov (KS) tests comparing the theoretical Poisson and actual 6-4 PP formation count distribution for each NNYYNN sequence. Gray markers are *p*-values derived from a UVDE-seq experiment on naked DNA treated with 400J/m^2 UVC (GEO accession GSE297236). Green diamond markers are the same *p*-values derived from cellular DNA shown in Supplementary Figure 2 for comparison. In contrast with CPD formation, 6-4 PP formation for NNYYNN sequences follow a Poisson process for both cellular and naked DNA. **(b)** Heatmap analysis showing significant enrichment and / or depletion of 6-4 PP formation in naked DNA in the same TF binding site regions for the 63 TF clusters shown in Figure 5a (Poisson test one-sided $p<0.01$, BH corrected). Analysis is based on a Poisson model of 6-4 PP formation conditioned on NNYYNN sequences in naked DNA. Source data are provided in Supplementary Data 3.

Supplementary Figure 7. TF binding effects on CPD formation are consistent across analytical and simulation analysis methodologies

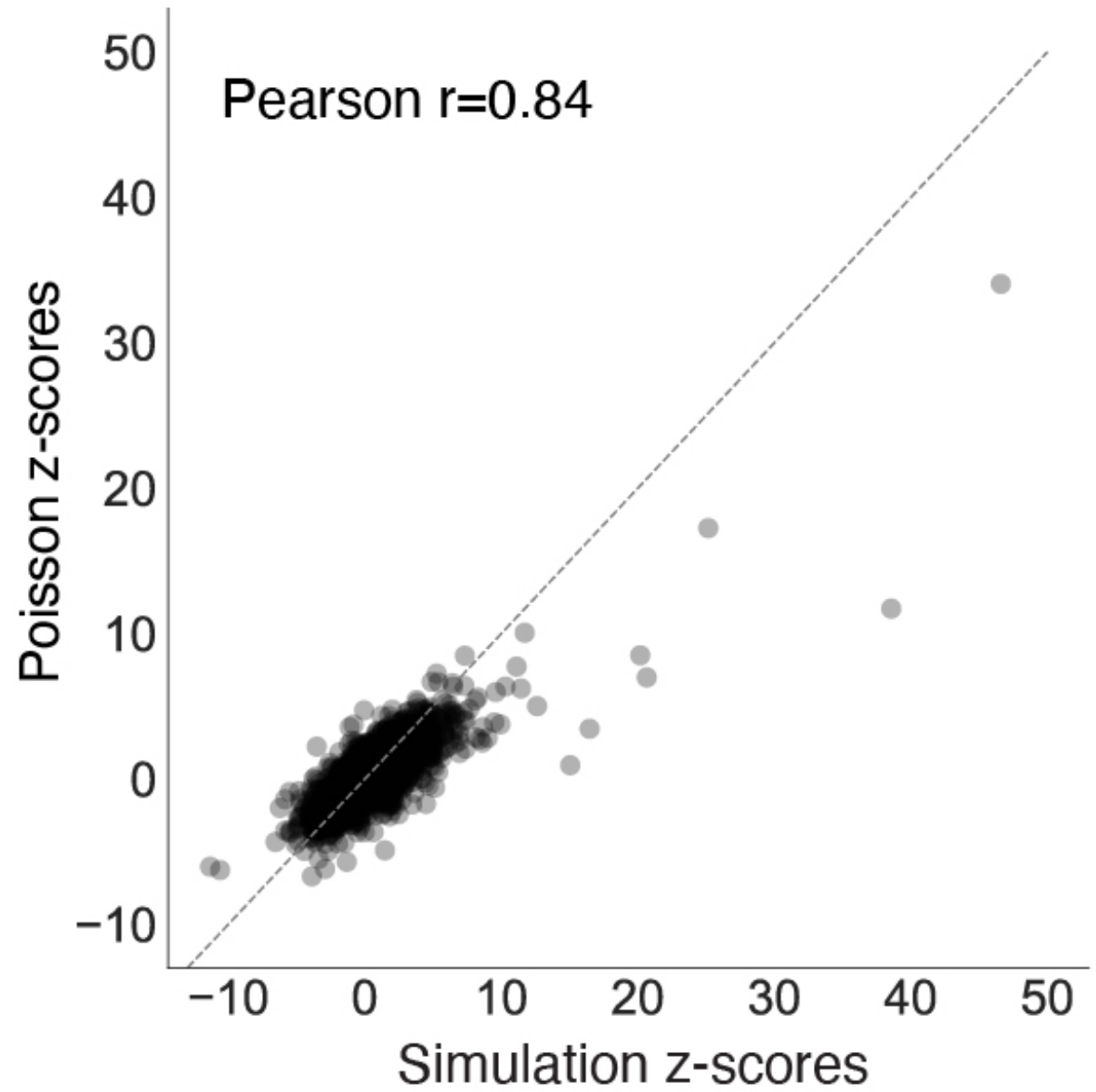

Scatter plot showing correlation between results of CPD formation analysis as evaluated by the simulation permutation testing method (x-axis) and analytical Poisson method (y-axis). Each marker is the z-score of an individual position in the TF binding site window across the 189 TF clusters with sufficient CPD signal for analysis (*n*=15,027). The permutation testing simulation was performed as described in the “Simulation of CPD formation and C>T transitions in TF binding sites” Supplementary Methods section. Poisson method is described in the “Poisson modelling of CPD formation at TF binding sites” Methods section. Source data are provided in the Source Data file.

Supplementary Figure 8. Schematic detailing comparative analysis approach for CPD-projected versus actual skin cancer C>T mutations

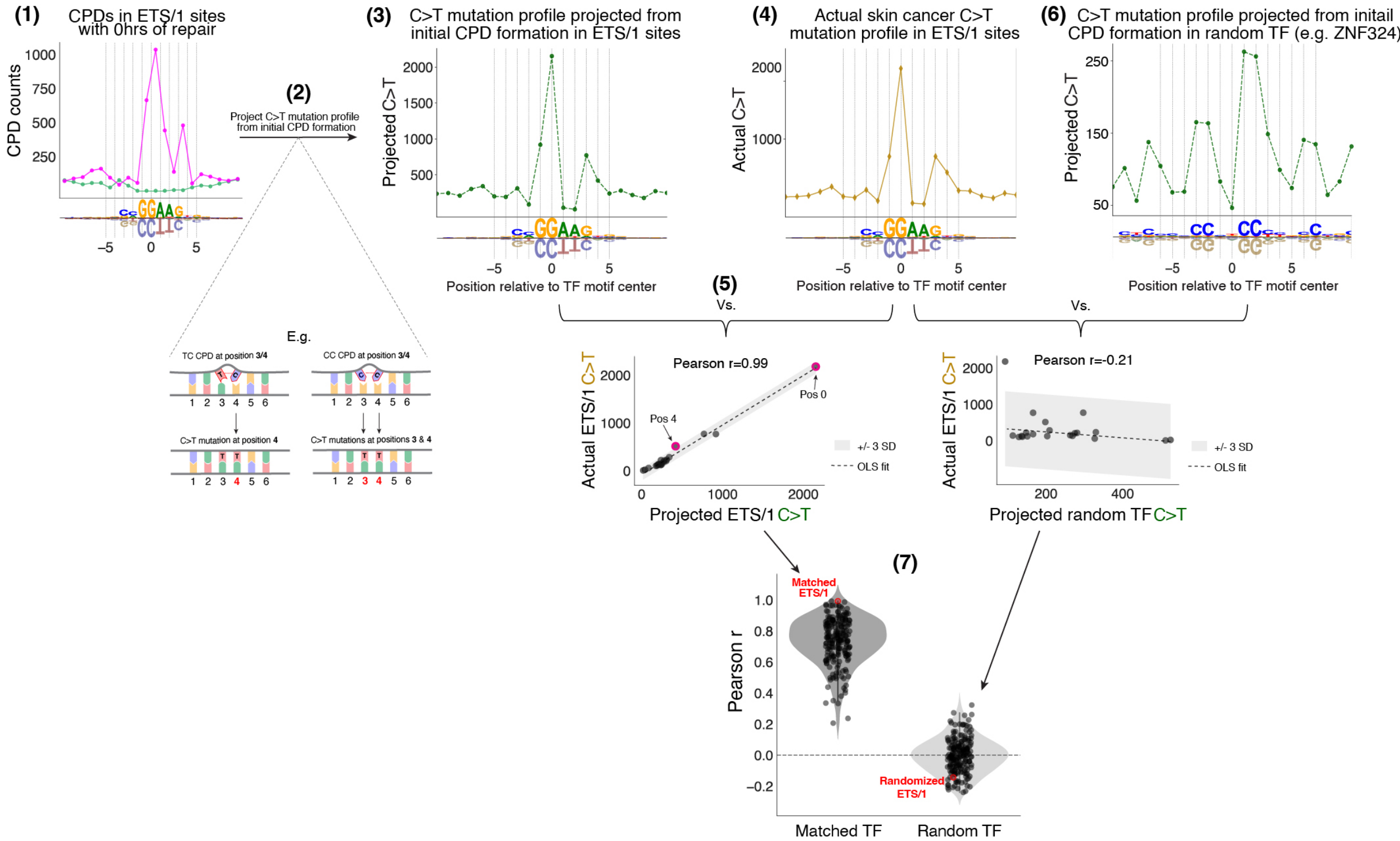

Schematic detailing our approach for comparing C>T mutations projected from initial CPD formation with actual C>T mutations from skin cancer samples (see Methods). (1) Projected C>T mutation profiles were generated by assuming all CPD-dimerized cytosines from CPD-seq v2.0 of human skin fibroblasts processed immediately after 6J/m^2 UVC irradiation were mutated to thymines. (2) For example, a CC CPD between two cytosines at positions i and $i+1$ would then be projected as a CC>TT tandem mutation at position i and position $i+1$. (3) These projected mutations were then aggregated per position across the TF binding sites for cumulative C>T mutation profiles and compared against the equivalent mutation profiles using C>T mutations from (4) actual skin cancers. (5) We calculated the Pearson's correlation coefficient between the projected and actual C>T profiles across all positions in the TF binding site. We used OLS regression on projected versus reported mutations to determine the best fit line. (6) As a null comparison, randomized Pearson correlation coefficients were generated by comparing the actual skin cancer C>T mutation profile of a given TF cluster with the projected C>T mutation profiles of 5 randomly selected TF clusters. (7) For the "Random TF" righthand violin plot in Figure 3a, each marker represents the average Pearson correlation coefficient between a given TF cluster and 5 of these randomized comparisons. All Pearson correlations were computed across TF binding site positions and their flanking regions (± 20 bp relative to the center of the binding site).

Supplementary Figure 9. The effect of sequence context on CPD repair

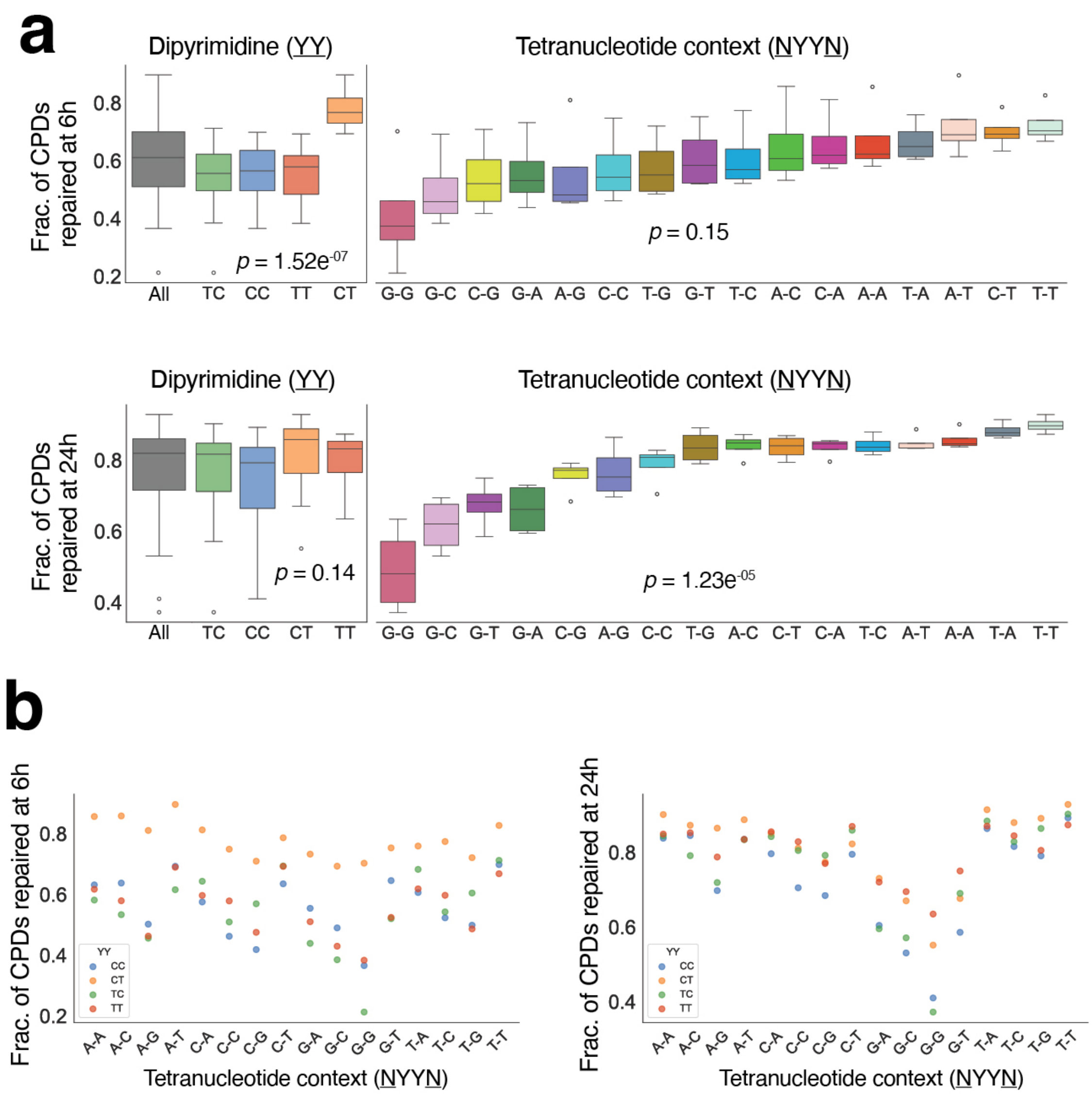

(a) Box plots of CPD repair efficiency grouped by dipyrimidine (YY, *n*=4) and NYYN (*n*=64) sequence context, calculated using CPD-seq v2.0 data in the same genomic regions described in Supplementary Figure 1a from cells irradiated with 6J/m² UVC and given either 6 (top) or 24 (bottom) hours for repair. Y-axis is defined as $1 - (6\text{hr CPD count}_k / 0\text{hr CDP count}_k)$ for a given k-mer, k. Included are Kruskal-Wallis test p-values for each sequence context grouping. **(b)** Scatter plot of CPD repair efficiency per tetranucleotide sequence context for cells given either 6h (left) or 24h (right) for repair after UVC treatment. The color of markers correspond to the central YY type. Source data are provided in Supplementary Data 2.

Supplementary Figure 10. Additional examples of CPD repair modulation at active TF binding sites

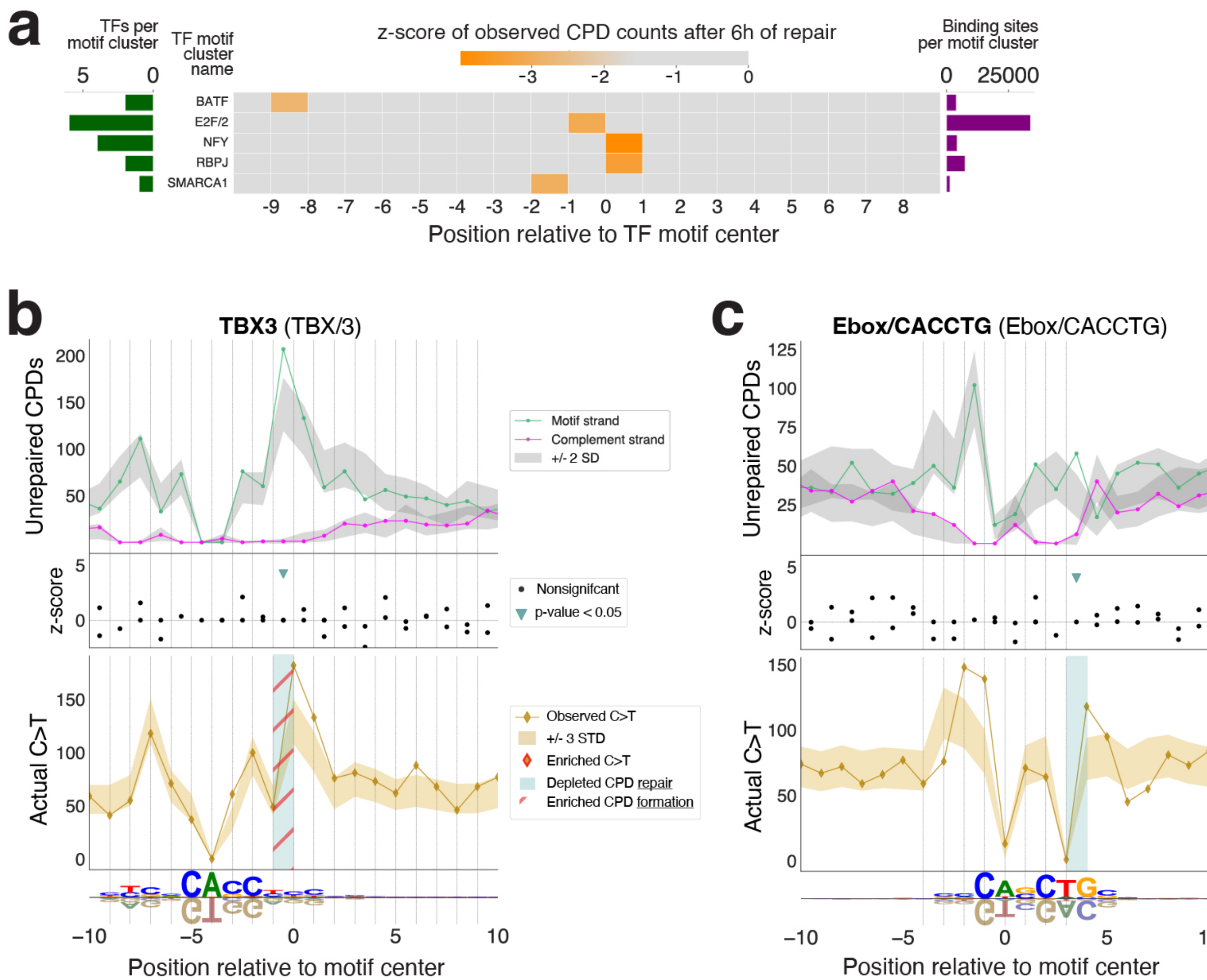

(a) Heatmap summarizing results of 5 TF clusters with significant repair enhancement in their binding sites (bootstrap test one-sided *p*<0.05, BH corrected). At each position, the z-score of unrepaired CPDs 6hrs after irradiation are the cell value, where a large negative z-score indicates significantly depleted levels of unrepaired CPDs, signifying enhanced repair. Each row is a specific TF cluster. The bar plots on each side of the heatmap illustrate the number of distinct TFs mapped to each TF cluster (left) and the number of binding sites attributed to each TF cluster (right). **(b)** Full analysis of CPD repair in active TBX3 binding sites (*n*=9,844) at 6h after irradiation with 6J/m^2. (Top) Observed unrepaired CPD counts aggregated by position on the motif (green) and motif-complement (pink) strand. Gray shaded region denotes ±2 standard deviations of the mean from 10,000 bootstrap simulations under the background repair model. (Middle) Scatter plot of z-scores per position, colored by strand. Triangle markers denote significant repair depletion (bootstrap test one-sided *p*<0.05, BH corrected). (Bottom) C>T mutation analysis for SOX binding sites. Mutations were aggregated across both DNA strands; gold region denotes the expected C>T mutation counts (mean ±3 SD) under the background mutation model. Red diamonds indicate significant C>T enrichment (one-sided bootstrap test of p<0.05, BH-corrected), teal shading represents repair depletion, and red crosshatching represents CPD formation enrichment. **(c)** Similar to panel b, but for active Ebox/CACCTG transcription factor binding sites (*n*=12,143). Source data are provided in Supplementary Data 3.

Supplementary Figure 11. CPD repair modulation in active CTCF binding sites

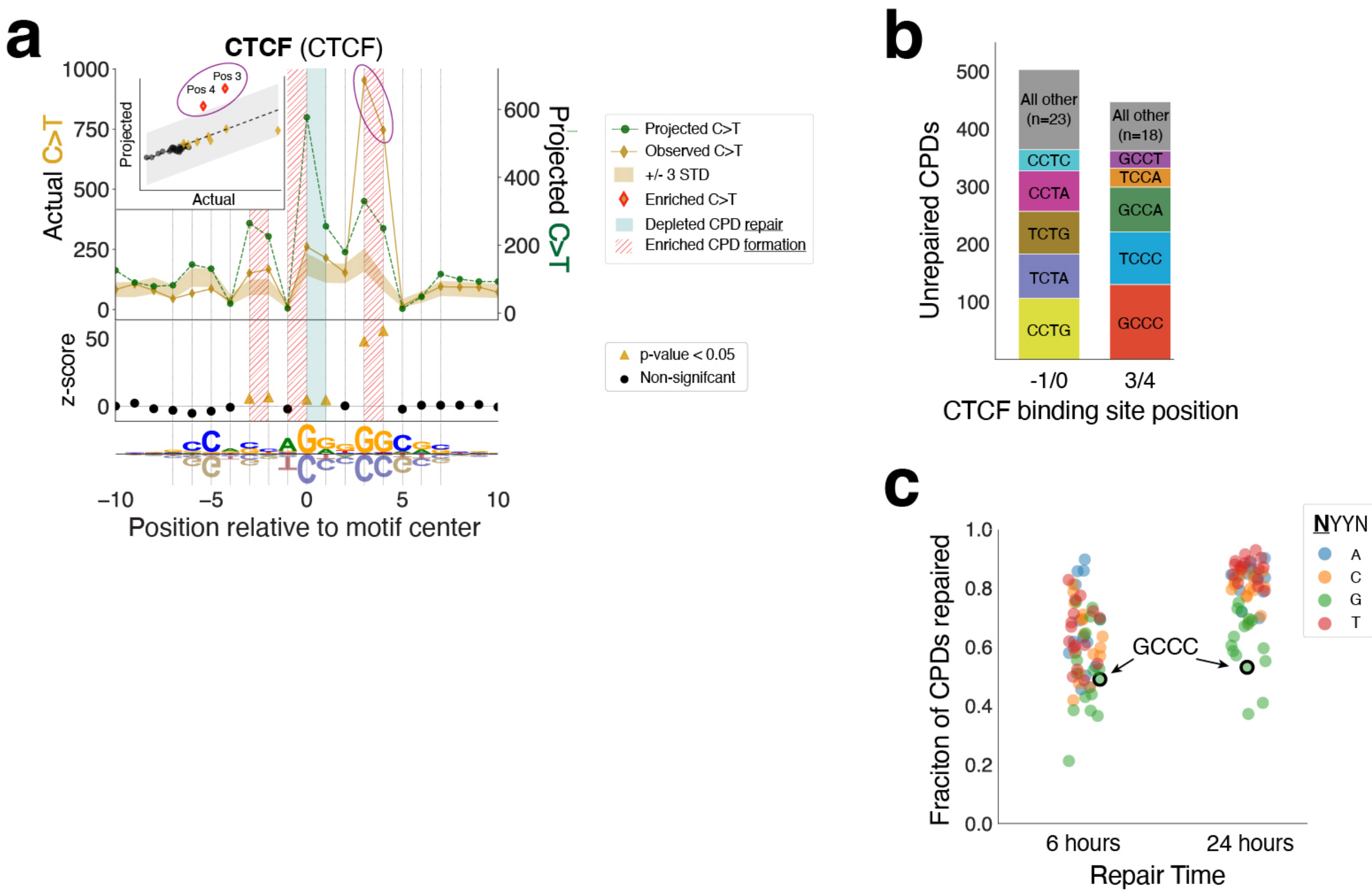

(a) Comparative analysis between the actual and projected C>T mutation profiles in CTCF binding sites (n=10,000). Dark green line shows the mutation counts projected from CPD counts (right y-axis), the gold line represents actual mutation counts from skin cancer samples (left y-axis). Mutations were aggregated across both DNA strands for a total mutation count per TF binding site position. Shaded gold region denotes the ±3 standard deviations of the mean from 10,000 bootstrapped simulations of C>T mutation counts based on a background mutation model (Methods). Z-scores of the observed mutation counts, according to the same background model, are shown for each position. Gold triangle markers denote positions with significantly modulated C>T mutation levels (one-sided bootstrap test, BH-corrected $p<0.05$), pointing in the direction of effect. Red diamonds indicate significant C>T enrichment (one-sided bootstrap test of $p<0.05$, BH-corrected), teal shading represents repair depletion, and red crosshatching represents CPD formation enrichment. Inset shows a direct comparison between the actual C>T mutation counts vs. the projected C>T mutation counts; each point is a position in the CTCF binding site. Gray shaded region is ±3 standard deviations from the ordinary least squares fit of the data (dashed line, Methods). Positions 3 and 4 are significantly discordant from the projected C>T CTCF profile and circled in both the inset and mutation profile (OLS residual error z-score > 2.58). **(b)** Bar plots of tetranucleotide content of CPDs formed immediately after 6J/m^2 UVC irradiation at positions -1/0 and 3/4 on the motif-complement strand of CTCF binding sites. **(c)** Strip plots showing the fraction of CPDs repaired for all 64 NYYN at 6 and 24 hours, where each marker is a unique tetranucleotide colored by their 5' nucleobase. GCCC is annotated. Source data are provided in Supplementary Data 2 & 3.

Supplementary Figure 12. General single base substitution mutation enrichment in NFY binding site flanks

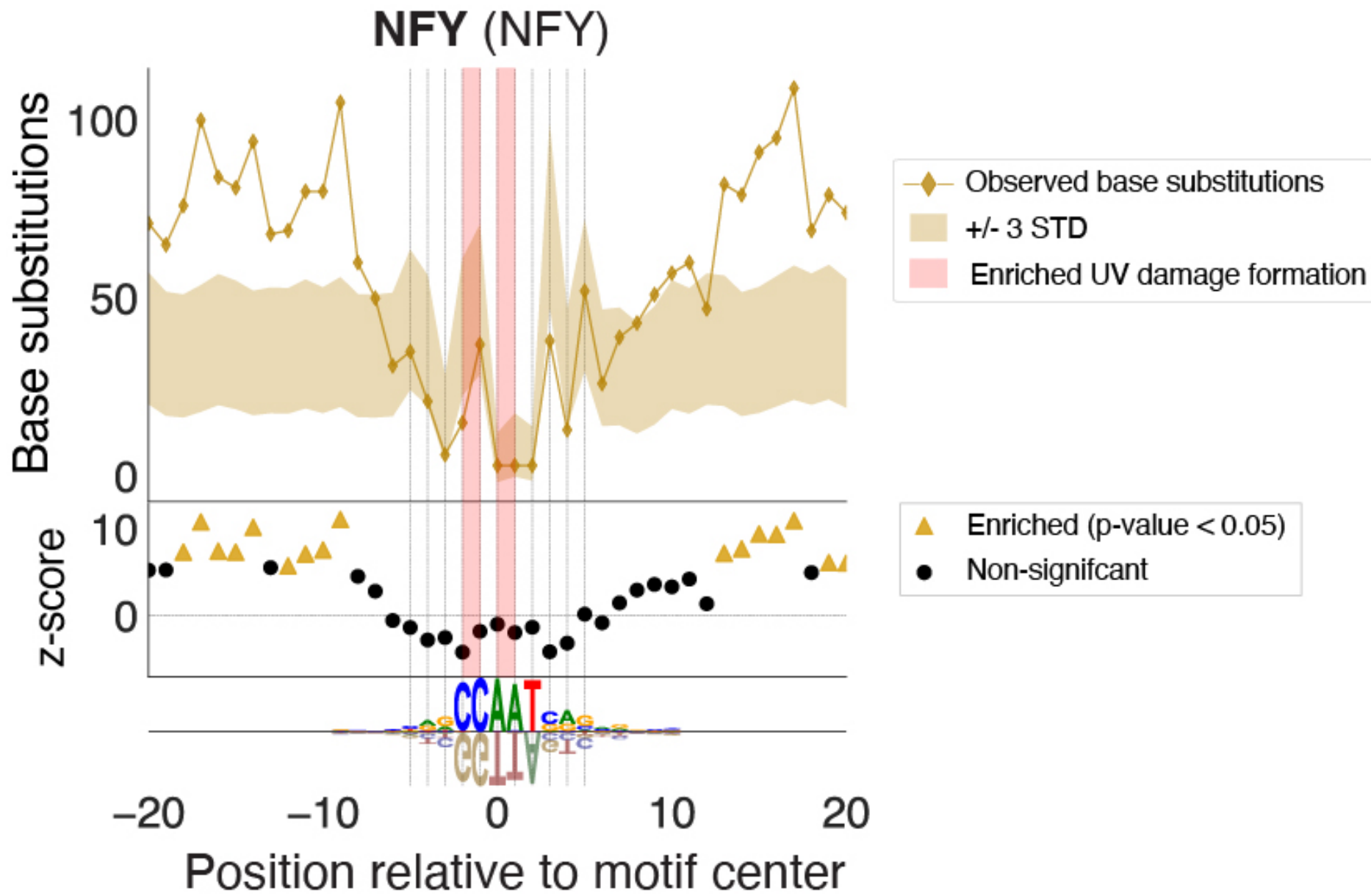

Analysis of all base substitution mutations in active NFY binding sites (*n*=4,195). Mutations were aggregated across both DNA strands for a total mutation count per TF binding site position. Shaded gold region denotes ±3 standard deviations for expected mutation counts according to an analytical background model of mutation frequency across all single base substitution types (Methods). Z-scores of the observed mutation counts, according to the same background model, are shown for each position. Gold triangle markers denote positions with significantly modulated C>T mutation levels (one-sided Poisson binomial test $p<0.05$, BH-corrected), pointing in the direction of effect. UV damage formation hotspot positions identified in our prior analyses are shaded red, where motif position -2/-1 was enriched for 6-4 PPs (z-score=42.69) and motif-complement position 0/1 was enriched for CPDs (z-score=11.21) immediately following UV irradiation. Source data are provided in the Source Data file.

Supplementary Figure 13. Structural analysis of TF-bound dipyrimidines using AlphaFold 3

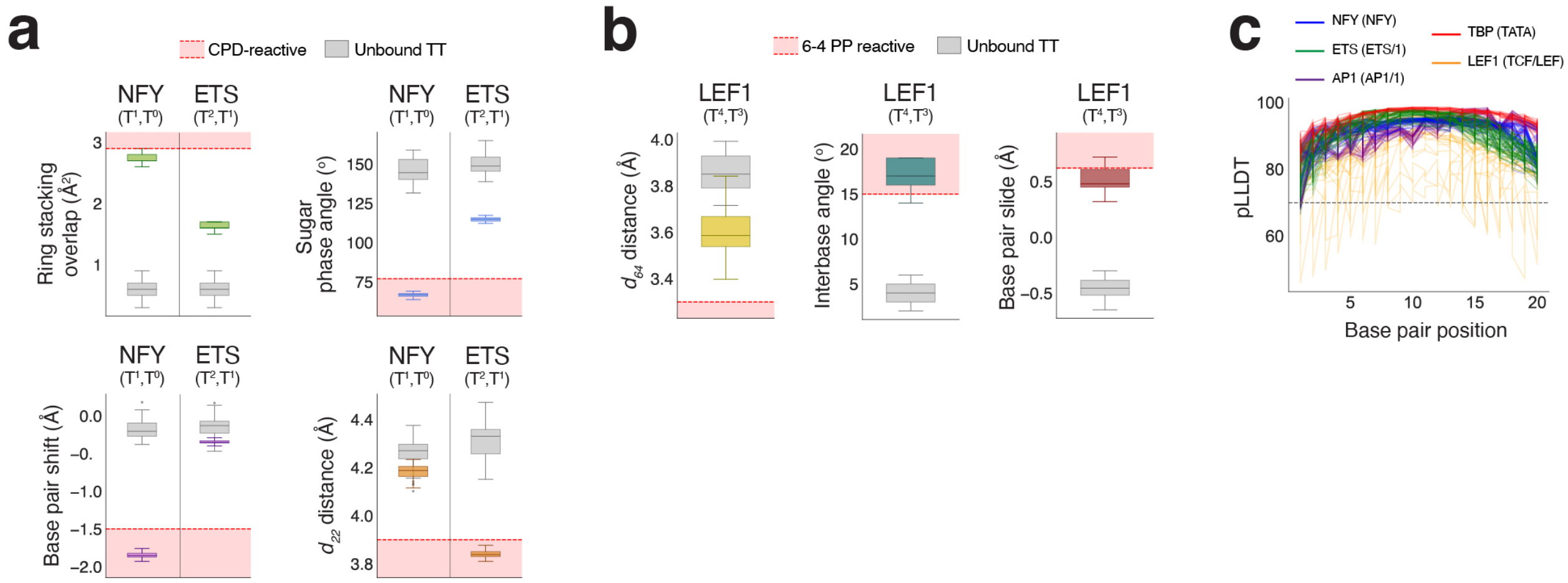

(a) As a control for NFY T^1T^0 (z-score=11.21), we performed a structural analysis (as described in “Structural analysis of AlphaFold 3 (AF3) predicted TF-DNA complexes” Methods section) for T^2T^1 in ETS binding sites, which is also a conserved TT but does not exhibit significant CPD formation enrichment (z-score=1.60). (Left) Boxplots of CPD-relevant structural parameters for T^1T^0 across NFY binding sites when bound by NFY (colored box plots, *n*=60) versus TT steps in corresponding unbound NFY structures (gray boxplots, *n*=46). (Right) Boxplots of CPD-relevant structural parameters for T^2T^1 across ETS binding sites when bound by ETS (colored box plots, *n*=60) versus TT steps in corresponding unbound ETS structures (gray boxplots, *n*=100). Only structures for which all DNA residues had a mean predicted local distance test score (pLDDT) ≥70 were used in the analysis (Methods). Center line, median; boxes limits, first and third quartiles; whiskers, most extreme points within 1.5× the interquartile range. Red shaded regions signify the CPD-reactivity thresholds characteristic of TT photodimer formation in unbound, duplexed DNA reported by Conti et al.[7] While ETS T^2T^1 in the TF-bound state shows a slight increase in base stacking, the average ring stacking overlap, base pair shift, and sugar phase angle remain well outside the thresholds for TT CPD photoreactivity. Interestingly, the ETS T^2T^1 does have a shortened d_{22} distance that falls in the CPD photoreactive range. This short C_5-C_6 interbond distance, in the absence of the necessary base stacking, does not appear to be sufficient for CPD formation enrichment. **(b)** As in a, but for 6-4 PP-relevant structural parameters for position 3/4 on the motif-complement strand of LEF1 binding sites (bound T^4T^3 *n*=20; unbound T-T *n*=20) which we found to be enriched for 6-4 PP formation (z-score=10.05). Red shading indicates 6-4 PP-reactivity thresholds for TT dipyrimidines from Conti et al.[7] **(c)** Line plots of average pLDDT scores per DNA residue position for AF3 structural predictions of NFY, ETS, AP1, TBP, and LEF1 binding sites, bound by their putative TF. The pLDDT score was averaged across each DNA residue for the top-ranking structural prediction per AF3 run. The “confident” pLDDT threshold (pLDDT>=70), per AF3 documentation[8], is shown as a dashed line. Source data are available in the Source Data file.

Supplementary Figure 14. Bootstrap modelling of CPD repair in TF binding sites using timecourse data

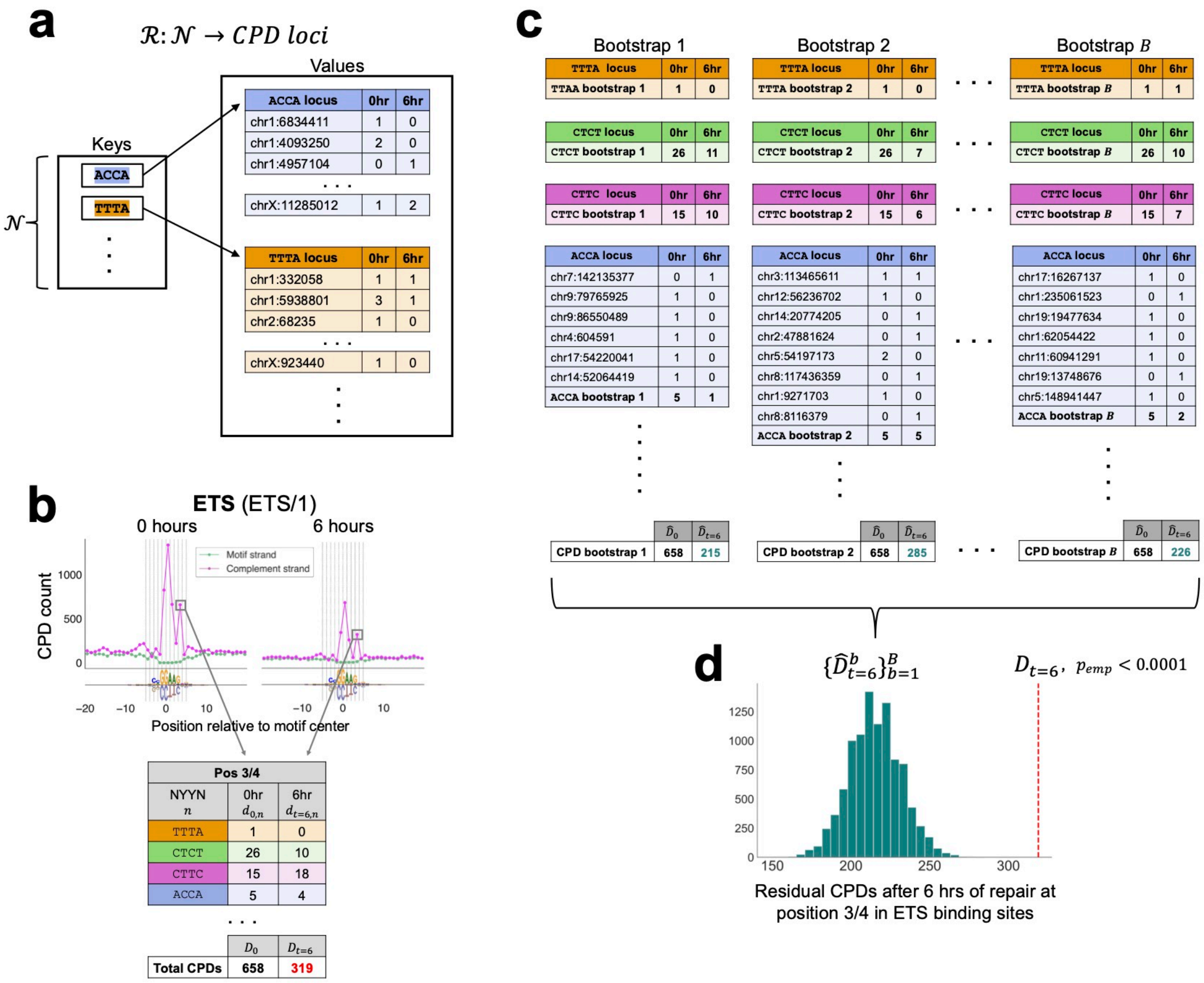

Accompanying figure to the "Simulation of CPD repair in TF binding sites" Supplementary Methods section. **(a)** Repair dictionary where all UV-damageable tetranucleotides (NYYN) are mapped to intergenic, open-chromatin CPD-seq v2.0 data from two repair timepoints and then merged on genomic locus. Each NYYN dictionary value consists of a dataframe where each row is a CPD genomic locus, where each locus has two columns containing the corresponding CPD counts at 0 and 6 hours. Genomic loci that were undamaged in both timepoints are superfluous to the analysis and therefore not included. **(b)** CPD profiles of aggregated ETS binding sites (TF motif cluster: ETS/1) immediately after (e.g. 0hrs, left) and 6 hours (right) after irradiation with 6J/m^2 UVC. Below is an example of the CPD sequence composition at position 3/4 on the motif-complement strand for both repair timepoints with the total CPD damage adding up to 658 and 319 for both 0 and 6 hours, respectively. The CPD counts at 0 hours we refer to as the CPD formation burden. **(c)** Schematic of bootstrapping strategy as described in the Supplementary Methods. For each NYYN, the repair dictionary is randomly sampled with replacement to predict the residual CPD counts after 6 hours of repair, such that the sampled CPD formation burden equals the observed CPD formation burden. The predicted residual CPD counts are summed across all NYYN to obtain the total predicted CPD level after 6 hours of repair. This bootstrap sampling is repeated many times to generate an empirical distribution to compare against observed CPD levels at 6 hours for hypothesis testing. **(d)** Histogram of bootstrapped (B=10,000) residual CPD counts at ETS motif-complement position 3/4 after 6 hours of repair time. Our analysis shows that we observe significantly higher CPD levels (n=319) than

the null distribution at 6 hours, indicating that CPDs are repaired less efficiently when they form at this binding site position.

Supplementary Figure 15. UVDE-seq read counts per dinucleotide sequence

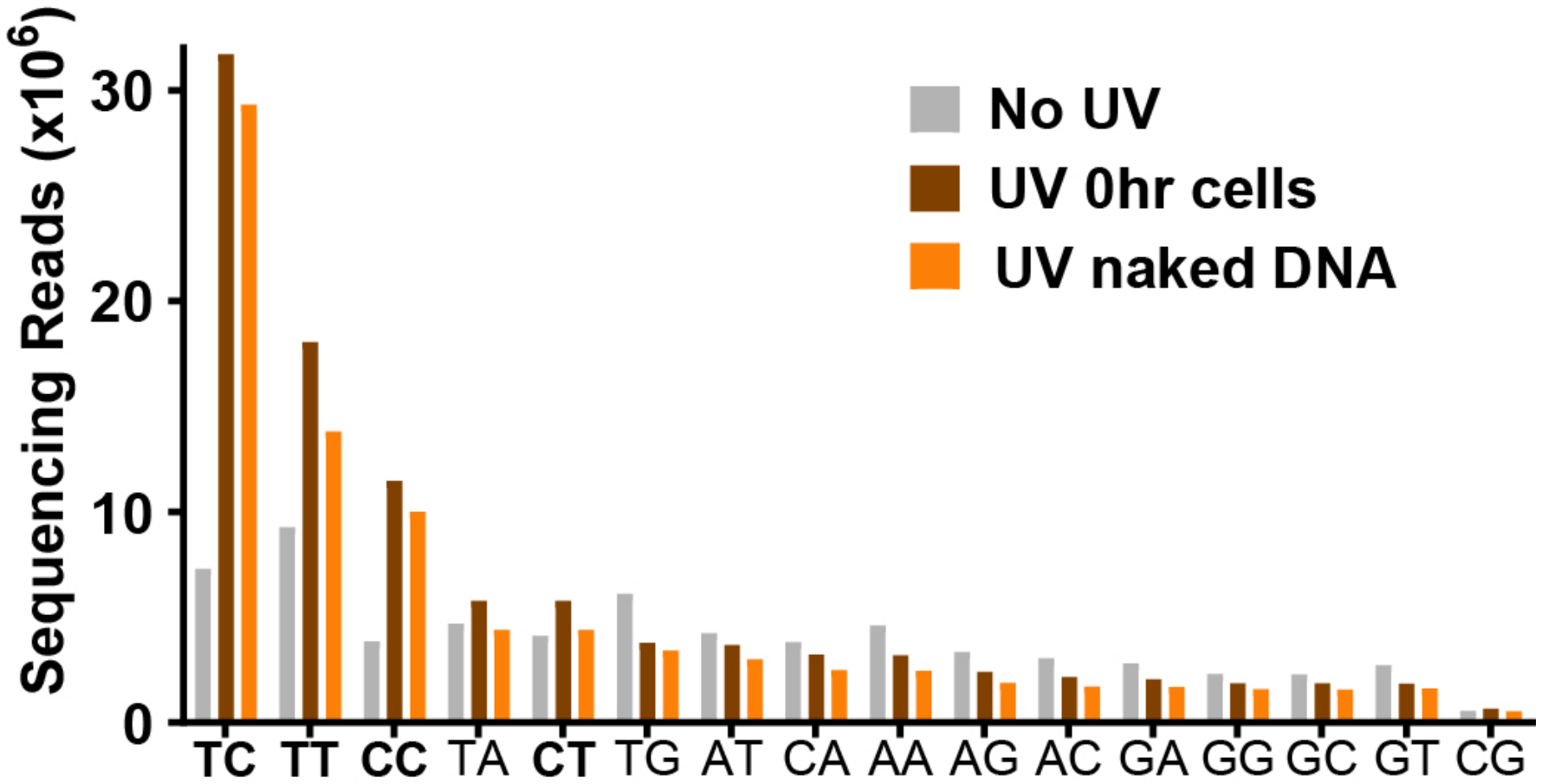

Counts of dinucleotides associated with each UVDE-seq read. Cellular DNA (brown) was irradiated with 500J/m^2 and naked DNA (orange) was irradiated with 400J/m^2 as described in the Methods section. The two nucleotides immediately upstream of the 5′ end of Read 1 on the opposite strand were collected and counted. 6-4 PPs are expected to occur primarily at dipyrimidines, with the expected abundance of lesions at TC > TT > CC > CT (all bolded)[9]. Source data are provided in the Source Data file.

SUPPLEMENTARY TABLES

Supplementary Table 1. Multiple linear regression of tetranucleotide sequence features on CPD repair efficiency

		Coefficient	*β (SE)*	*t*	*p-value*
**6 hrs of repair**<br>R^2=0.869<br>Adj. R^2=0.847	Intercept	0.630217	0.021194	29.736010	3.701761e-35
	YY: CT	0.219292	0.018956	11.568353	3.072142e-16
	5': G	-0.130028	0.018956	-6.859391	6.969866e-09
	3': G	-0.114892	0.018956	-6.060921	1.361651e-07
	3': C	-0.054716	0.018956	-2.886459	5.590889e-03
	3': T	0.052153	0.018956	2.751253	8.064044e-03
	5': C	-0.032108	0.018956	-1.693821	9.606070e-02
	YY: TC	-0.014730	0.018956	-0.777041	4.405256e-01
	5': T	-0.006274	0.018956	-0.330962	7.419542e-01
	YY: TT	-0.001696	0.018956	-0.089444	9.290597e-01
**24 hrs of repair**<br>R^2=0.877<br>Adj. R^2=0.857	Intercept	0.814320	0.017898	45.497084	9.564602e-45
	5': G	-0.216666	0.016009	-13.534284	5.435694e-19
	3': G	-0.096880	0.016009	-6.051716	1.408788e-07
	YY: CT	0.083589	0.016009	5.221484	2.911058e-06
	YY: TT	0.071993	0.016009	4.497113	3.691986e-05
	3': C	-0.038298	0.016009	-2.392330	2.025131e-02
	5': T	0.037196	0.016009	2.323477	2.394812e-02
	YY: TC	0.031564	0.016009	1.971662	5.378042e-02
	5': C	-0.024556	0.016009	-1.533908	1.308908e-01
	3': T	0.004867	0.016009	0.304046	7.622615e-01

Multiple ordinary least squares (OLS) regression analysis was performed using the Python statsmodel.formula.api OLS implementation (v0.14.0). Regression was used to assess tetranucleotide (NYYN) sequence features as predictors of repair efficiency in 6J/m^2 UVC irradiated human skin fibroblasts after 6 and 24 hours of repair. Repair efficiency (i.e. fraction of CPDs repaired) was defined as $1 - (0\text{hr CPD count}/X\text{hr CPD count})$ and derived from all intergenic, open chromatin regions to isolate effects of GG-NER. We note that our results agree with previous studies analyzing sequence specificity of CPD excision using XR-seq data[10,11], with earlier repair (within 6 hours) being highly biased towards CT dipyrimidines, while consistent across both timepoints, dipyrimidines flanked by guanines were repaired significantly slower.

SUPPLEMENTARY METHODS AND MATERIALS

Simulation of CPD repair in TF binding sites

CPD-seq v2.0 timecourse data of human skin fibroblast cells given 0 or 6 hours of repair time after irradiation with 6J/m^2 UVC was used to analyze CPD repair in aggregated TF binding sites using bootstrap sampling. In Supplementary Figure 9 and Supplementary Table 1, we see that dipyrimidine type and tetranucleotide sequence context significantly impacts CPD repair efficiency. Because we are interested in the repair trends of aggregated damage in TF binding sites per position, it is important to consider the relationship between repair efficiency and the amount of cumulative CPD damage formed immediately after irradiation.

CPD formation is a rare, stochastic event such that the damage landscape between CPD-seq experiments is heterogeneous. This means that the same genomic locus between two independent CPD experiments will rarely form a CPD in both cases. While this heterogeneity is theoretically unbiased when estimating CPD repair efficiency when there is a sufficient starting amount of CPDs, as the number of initial CPDs decreases, the signal-to-noise ratio also diminishes, limiting statistical assessment of repair.

Thus, in our analysis of repair efficiency, we consider the residual CPD count, D_t at a given aggregated TF binding site position after a defined repair interval, t, to be dependent on both sequence composition and the CPD damage formation burden immediately after irradiation, D_0.

To serve as a null distribution, we created a synthetic dataset in the form of a repair dictionary, $\mathcal{R}$, where all UV-damageable tetranucleotides (NYYN) are mapped to CPD-seq v2.0 data from two repair timepoints and then merged on genomic locus (Supplementary Figure 14a). The data in $\mathcal{R}$ is derived from intergenic, open chromatin regions to best capture cis-regulatory regions that we assume are the primary binding targets for TFs[12], focusing our analysis on GG-NER mechanisms and excluding TC-NER activity that is mostly active in genic introns and exons[13].

Let p be a position in TF's aggregated binding sites and $\mathcal{N} = \{\mathrm{NYYN}\}$ represent the set of all 64 UV-damageable tetranucleotide sequences.

The total initial CPD formation burden at p, is distributed across $\mathcal{N}$ in varying proportions (Supplementary Figure 14b).

For each sequence $n \in \mathcal{N}$, at position p, define the observed CPD formation burden as $d_{0,n}$.

To simulate repair, we randomly sampled $\mathcal{R}(n)$ with replacement such that,

$$\hat{d}_{0,n} = d_{0,n}$$

where $\hat{d}_{0,n}$ is the sampled CPD formation burden from $\mathcal{R}(n)$ (Supplementary Figure 14c).

Because $\mathcal{R}$ contains genomic loci with paired CPD counts measured at timepoints 0 and t, each sampling is accompanied by the corresponding residual CPD counts for n given repair time t, denoted as $\hat{d}_{t,n}$.

To determine the total predicted CPD damage at p after t hours of repair, we sum $\hat{d}_{t,n}$ across $\mathcal{N}$:

$$\widehat{D}_t = \sum_{n\in\mathcal{N}} \hat{d}_{t,n}$$

We repeat this process for $B = 10{,}000$ bootstrap samples to create an empirical distribution, $\{\widehat{D}_t^b\}_{b=1}^B$ of total residual CPDs at p given repair t (Supplementary Figure 14d).

Using this empirical distribution as our null, we then perform hypothesis testing at p, to assess if the observed residual CPD signal is smaller than expected. Such that for samples in $\{\widehat{D}_t^b\}_{b=1}^B$,

$$H_0: D_t \le \widehat{D}_t^b, \text{ and}$$

$$H_A: D_t > \widehat{D}_t^b$$

We calculate a one-sided empirical p-value as follows:

$$P_{emp} = \frac{1}{B}\sum_{b=1}^{B} I\left[\widehat{D}_t^b \ge D_t\right].$$

If the $P_{emp} < 0.05$ (after BH correction), we reject the null hypothesis and say that the observed residual CPD signal at p is significantly higher than expected after t repair time, indicating that CPDs are repaired less efficiently when they form at p. We infer that such effects are the result of TFs stills successfully complexing with their binding sites even when these affected positions are dimerized as a CPDs, thus competing with repair factors for UV lesion recognition and attenuating the repair process.

Simulation of CPD formation and C>T transitions in TF binding sites

We used a simulation-based method to quantify the significance and magnitude of CPD and C>T mutation enrichment and depletion patterns at each position in the TF binding site windows of the 225 TF motif clusters we curated for the study. This procedure involves a two-step process. First, CPD and C>T counts across intergenic, accessible chromatin regions were extracted, and aggregated by immediate sequence (NYYN for CPDs, and the (NYN for mutations). Then, the total counts observed at each sequence were redistributed uniformly across all positions matching that sequence (or its reverse complement) within the targeted region. This approach preserves the original global damage and mutation counts for each sequence context through multinomial randomization (numpy.random.multinomial), while randomizing the specific genomic locations of these events. This redistribution was performed 10,000 times to model the background CPD and mutation distributions based solely on sequence context.

To evaluate the TF-binding effects on CPD formation and C>T enrichment, we intersected active TF binding site windows (here, defined as ± 20 base pairs relative to the center of the TF cluster binding motif) with actual counts and then again with simulated counts to create a per-position null distributions for CPD formation and C>T enrichment, respectively. Then, the expected counts from the background model were scaled to match the mean frequencies of CPDs or C>Ts in the flanks (defined as further than five base pairs outside of the motif). This scaling was done separately for each strand (both counts on the motif and complement strand) and each half of the TF motif. We then calculated one-sided empirical p-values to quantify the statistical significance of both enrichment and depletion at each position; the *p*-value for enrichment was calculated as the proportion of simulated counts (post-adjustment; out of 10,000) that exceeded

the observed count at that position, and the p-value for depletion was calculated as the proportion of simulated counts that were less than the observed count. BH-corrected *p*-value multiple test correction was then applied across all positions in the TF binding site window. Additionally, we calculated the mean and standard deviation of the simulated null distribution at each position for z-scores, allowing us to compare the magnitudes of enrichment or depletion effects at each position.

ATAC-seq oligos and adaptors

Ad1_noMX: AATGATACGGCGACCACCGAGATCTACACTCGTCGGCAGCGTCAGATGTG

Ad2.1: CAAGCAGAAGACGGCATACGAGATTCGCCTTAGTCTCGTGGGCTCGGAGATGT

Ad2.2: CAAGCAGAAGACGGCATACGAGATCTAGTACGGTCTCGTGGGCTCGGAGATGT

Ad2.3: CAAGCAGAAGACGGCATACGAGATTTCTGCCTGTCTCGTGGGCTCGGAGATGT

Ad2.4: CAAGCAGAAGACGGCATACGAGATGCTCAGGAGTCTCGTGGGCTCGGAGATGT

Ad2.5: CAAGCAGAAGACGGCATACGAGATAGGAGTCCGTCTCGTGGGCTCGGAGATGT

Ad2.6: CAAGCAGAAGACGGCATACGAGATCATGCCTAGTCTCGTGGGCTCGGAGATGT

Ad2.7: CAAGCAGAAGACGGCATACGAGATGTAGAGAGGTCTCGTGGGCTCGGAGATGT

Ad2.8: CAAGCAGAAGACGGCATACGAGATCCTCTCTGGTCTCGTGGGCTCGGAGATGT

Ad1.1:
AATGATACGGCGACCACCGAGATCTACACTAGATCGCTCGTCGGCAGCGTCAGATGTG

Ad1.2:
AATGATACGGCGACCACCGAGATCTACACCTCTCTATTCGTCGGCAGCGTCAGATGTG

Ad1.3:
AATGATACGGCGACCACCGAGATCTACACTATCCTCTTCGTCGGCAGCGTCAGATGTG

Ad1.4:
AATGATACGGCGACCACCGAGATCTACACAGAGTAGATCGTCGGCAGCGTCAGATGTG

Ad1.5:
AATGATACGGCGACCACCGAGATCTACACGTAAGGAGTCGTCGGCAGCGTCAGATGTG

Ad1.6:
AATGATACGGCGACCACCGAGATCTACACACTGCATATCGTCGGCAGCGTCAGATGTG

Ad1.7:
AATGATACGGCGACCACCGAGATCTACACAAGGAGTATCGTCGGCAGCGTCAGATGTG

Ad1.8:
AATGATACGGCGACCACCGAGATCTACACCTAAGCCTTCGTCGGCAGCGTCAGATGTG

UVDE-seq oligos and adaptors

Oligos for first adapter ligation:

trP1-top (5′-CCTCTCTATGGGCAGTCGGTGAT-phosphorothioate-T-3′)

trP1-bottom (5′-phosphate-ATCACCGACTGCCCATAGAGAGGC-dideoxy-3′).

Oligos for second adapter ligation:

A1-top (5′-phosphate-ATCCTCTTCTGAGTCGGAGACACGCAGGGATGAGATGGCdideoxy-3′), A1-bottom (5′-biotin-CCATCTCATCCCTGCGTGTCTCCGACTCAGAAGAGGATNNNNNN-C3 phosphoramidite-3′); A2-top (5′-phosphate-ATCACGAACTGAGTCGGAGACACGCAGGGATGAGATGGCdideoxy-3′), A2-bottom (5′-biotin-CCATCTCATCCCTGCGTGTCTCCGACTCAGTTCGTGATNNNNNN-C3 phosphoramidite-3′); A3-top (5′-phosphate-ATCTCAGGCTGAGTCGGAGACACGCAGGGATGAGATGGCdideoxy-3′), A3-bottom (5′-biotin-CCATCTCATCCCTGCGTGTCTCCGACTCAGCCTGAGATNNNNNN-C3 phosphoramidite-3′). A4-top (5’-phosphate-ATCGCGATCTGAGTCGGAGACACGCAGGGATGAGATGGCdideoxy-3’); A4-bottom (5’- biotin-CCATCTCATCCCTGCGTGTCTCCGACTCAGATCGCGATNNNNNN-C3 phosphoramidite-3’); A5-top (5′- phosphate-ATCCAGTACTGAGTCGGAGACACGCAGGGATGAGATGGCdideoxy-3′); A5-bottom (5′- biotin-CCATCTCATCCCTGCGTGTCTCCGACTCAGTACTGGATNNNNNN-C3 phosphoramidite-3′) A6-top (5′- phosphate-ATCAGTTCCTGAGTCGGAGACACGCAGGGATGAGATGGCdideoxy-3′); A6-bottom (5′- biotin-CCATCTCATCCCTGCGTGTCTCCGACTCAGGAACTGATNNNNNN-C3 phosphoramidite-3′)

Oligonucleotides used for PCR confirmation and library amplification:

Primer A (5′-CCATCTCATCCCTGCGTGTCTCCGAC-3′)

Primer trP1 (5′-CCTCTCTATGGGCAGTCGGTGATT-3′).

SUPPLEMENTARY REFERENCES

1. Duan, M. *et al.* High UV damage and low repair, but not cytosine deamination, stimulate mutation hotspots at ETS binding sites in melanoma. *Proceedings of the National Academy of Sciences* **121**, e2310854121 (2024).

2. Lu, C., Gutierrez-Bayona, N. E. & Taylor, J.-S. The effect of flanking bases on direct and triplet sensitized cyclobutane pyrimidine dimer formation in DNA depends on the dipyrimidine, wavelength and the photosensitizer. *Nucleic Acids Research* **49**, 4266–4280 (2021).

3. Law, Y. K., Forties, R. A., Liu, X., Poirier, M. G. & Kohler, B. Sequence-dependent thymine dimer formation and photoreversal rates in double-stranded DNA. *Photochemical & Photobiological Sciences* **12**, 1431–1439 (2013).

4. Pan, Z., Chen, J., Schreier, W. J., Kohler, B. & Lewis, F. D. Thymine Dimer Photoreversal in Purine-Containing Trinucleotides. *J. Phys. Chem. B* **116**, 698–704 (2012).

5. Vierstra, J. *et al.* Global reference mapping of human transcription factor footprints. *Nature* **583**, 729–736 (2020).

6. Korhonen, J., Martinmäki, P., Pizzi, C., Rastas, P. & Ukkonen, E. MOODS: fast search for position weight matrix matches in DNA sequences. *Bioinformatics* **25**, 3181–3182 (2009).

7. Conti, I. *et al.* Multiple Electronic and Structural Factors Control Cyclobutane Pyrimidine Dimer and 6–4 Thymine–Thymine Photodimerization in a DNA Duplex. *Chemistry – A European Journal* **23**, 15177–15188 (2017).

8. Abramson, J. *et al.* Accurate structure prediction of biomolecular interactions with AlphaFold 3. *Nature* **630**, 493–500 (2024).

9. Bohm, K. A. *et al.* Genome-wide maps of rare and atypical UV photoproducts reveal distinct patterns of damage formation and mutagenesis in yeast chromatin. *Proc. Natl. Acad. Sci.* **120**, e2216907120 (2023).

10. Hu, J., Adar, S., Selby, C. P., Lieb, J. D. & Sancar, A. Genome-wide analysis of human global and transcription-coupled excision repair of UV damage at single-nucleotide resolution. *Genes & development* **29**, 948–960 (2015).

11. Morledge-Hampton, B., Kalyanaraman, A. & Wyrick, J. J. Analysis of cytosine deamination events in excision repair sequencing reads reveals mechanisms of incision site selection in NER. *Nucleic Acids Research* **52**, 1720–1735 (2024).

12. Panigrahi, A. & O'Malley, B. W. Mechanisms of enhancer action: the known and the unknown. *Genome Biology* **22**, 108 (2021).

13. Fousteri, M. & Mullenders, L. H. Transcription-coupled nucleotide excision repair in mammalian cells: molecular mechanisms and biological effects. *Cell Research* **18**, 73–84 (2008).
