# Supplementary material for: High-throughput characterization of transcription factors that modulate UV damage formation and repair at single-nucleotide resolution
Source: Nat Commun. 2026 Jul 10;17:8506. doi: 10.1038/s41467-026-75115-4 (PMC13483863; doi:10.1038/s41467-026-75115-4)
Supplement: Supplementary file 3 [file 41467_2026_75115_MOESM2_ESM.pdf]

Description of Additional Supplementary Files

Supplementary Data 1. Full list of TF motif clusters and TF proteins used in the study and summary of their binding effect on CPD formation, CPD repair, and 6-4 PP formation.

Supplementary Data 2. CPD and 6-4 PP formation frequencies and CPD repair efficiencies by DNA sequence context. Measurements were derived from chromatin accessible, intergenic regions of UV-irradiated human skin fibroblasts that were used for model development.

Supplementary Data 3. Detailed results of CPD formation, 6-4 PP formation, and CPD repair analyses across active TF motif cluster binding sites.

Supplementary Data 4. Detailed results of C>T mutation analysis across active TF motif cluster binding sites.

Supplementary Data 5. Full list of TF binding site sequences and TF protein sequences used in AlphaFold3 runs, and detailed structural measurements for TF binding site positions and dipyrimidines of interest.

Supplementary Data 6. Run summary, metadata, and QC results for ATAC-seq experiments in C1SAN/CSB^{WT} cells.
