# Supplementary material for: High-throughput characterization of transcription factors that modulate UV damage formation and repair at single-nucleotide resolution
Source: Nat Commun. 2026 Jul 10;17:8506. doi: 10.1038/s41467-026-75115-4 (PMC13483863; doi:10.1038/s41467-026-75115-4)
Supplement: Supplementary file 4 [file 41467_2026_75115_MOESM9_ESM.pdf]

nature portfolio

Corresponding author(s): Raluca Gordan, Hana I. Wasserman

Last updated by author(s): 06/08/2026

Reporting Summary

Nature Portfolio wishes to improve the reproducibility of the work that we publish. This form provides structure for consistency and transparency in reporting. For further information on Nature Portfolio policies, see our Editorial Policies and the Editorial Policy Checklist.

Statistics

For all statistical analyses, confirm that the following items are present in the figure legend, table legend, main text, or Methods section.

n/a	Confirmed	
☐	☒	The exact sample size (*n*) for each experimental group/condition, given as a discrete number and unit of measurement
☐	☒	A statement on whether measurements were taken from distinct samples or whether the same sample was measured repeatedly
☐	☒	The statistical test(s) used AND whether they are one- or two-sided<br>*Only common tests should be described solely by name; describe more complex techniques in the Methods section.*
☐	☒	A description of all covariates tested
☐	☒	A description of any assumptions or corrections, such as tests of normality and adjustment for multiple comparisons
☐	☒	A full description of the statistical parameters including central tendency (e.g. means) or other basic estimates (e.g. regression coefficient) AND variation (e.g. standard deviation) or associated estimates of uncertainty (e.g. confidence intervals)
☐	☒	For null hypothesis testing, the test statistic (e.g. *F*, *t*, *r*) with confidence intervals, effect sizes, degrees of freedom and *P* value noted<br>*Give P values as exact values whenever suitable.*
☒	☐	For Bayesian analysis, information on the choice of priors and Markov chain Monte Carlo settings
☒	☐	For hierarchical and complex designs, identification of the appropriate level for tests and full reporting of outcomes
☐	☒	Estimates of effect sizes (e.g. Cohen's *d*, Pearson's *r*), indicating how they were calculated

Our web collection on statistics for biologists contains articles on many of the points above.

Software and code

Policy information about availability of computer code

Data collection	In silico TF-DNA complexes were generated using the AlphaFold 3 web server (2025.06.10 release).
Data analysis	Our analysis pipeline of TF binding effects on UV damage formation and repair was made using custom Python 3.11 code (available at https://github.com/HanaWasserman/UV_damage_TFBS_analysis) using BEDTools 2.13.0, Pandas 2.3.2, Numpy 1.26.1, Scipy 1.13.1, Scikit-learn 1.7.1, Statsmodels 0.14.0, Matplotlib 3.8.1, Seaborn 0.13.2, Logomaker 0.8, Mygene 3.2.2. TF-DNA complexes were analyzed with custom Python 3.11 and bash scripts, using X3DNA-DSSR v2.5.0 and PyMOL 3.1.0. UVDE-seq assay raw FASTQ files were aligned to the human genome assembly hg19 and converted to BAM files using bowtie 2-2.2.6 and SAMtools 1.10. Raw ATAC-seq assay sequencing data was processed using the ENCODE ATAC-seq pipeline 2.2.3 (https://github.com/ENCODE-DCC/atac-seq-pipeline/tree/master) and aligned to the human reference genome assembly hg19. ATAC-seq peaks were converted to hg19 using University of California Santa Cruz Genome Browser LiftOver web server tool (accessed 2024).

For manuscripts utilizing custom algorithms or software that are central to the research but not yet described in published literature, software must be made available to editors and reviewers. We strongly encourage code deposition in a community repository (e.g. GitHub). See the Nature Portfolio guidelines for submitting code & software for further information.

Data

Policy information about availability of data

All manuscripts must include a data availability statement. This statement should provide the following information, where applicable:

- Accession codes, unique identifiers, or web links for publicly available datasets
- A description of any restrictions on data availability
- For clinical datasets or third party data, please ensure that the statement adheres to our policy

The data that support the findings in this study are available as Supplementary Data files in Excel format. The source data for violin, strip, scatter, box, and bar plots in both Main and Supplementary Figures are included in the Source Data file. We also include a sheet in the Source Data ("Additional Figure Info") detailing which Supplementary Data file was used to generate each figure. The UVDE-seq data generated for the study were deposited in the Gene Expression Omnibus (GEO) under accession number GSE297236. The ATAC-seq data generated for study the C1SAN/CSBWT cell line and peak calls were deposited in GEO under accession number GSE309644. For analysis of CPDs, we used a CPD-seq v2.0 dataset published by Duan et al. and deposited under GEO accession GSE235483. TF binding site curation was done from previous published binding site calls by Vierstra J., et al: https://doi.org/10.1038/s41586-020-2528-x, found at https://www.vierstra.org/resources/motif_clustering. In vitro CPD damage formation measurements in varying sequence contexts were curated manually from a paper by Lu C., et al: https://doi.org/10.1093/nar/gkab214. Protein sequences used to generate the AlphaFold3 structures for TF-DNA complex structural analysis can be found in the UniProt Knowledgebase under IDs: P01100, P05412 (AP1); P14921-1 (ETS1); P23511-1, P25208, Q13952-1 (NFY); P20226 (TBP); Q9UJU2 (LEF1). The 360 AlphaFold 3 structures generated for the study have been submitted and accepted to ModelArchive under Project ID: ma-wasserm-tfbs. Gene expression data of human skin fibroblasts included in Supplementary Table 1 is from RNA-seq experiments as part of the ENCODE project consortium (ENCODE: ENCSR000CUH, GEO accession: GSE78610).

Research involving human participants, their data, or biological material

Policy information about studies with human participants or human data. See also policy information about sex, gender (identity/presentation), and sexual orientation and race, ethnicity and racism.

Reporting on sex and gender	Not applicable
Reporting on race, ethnicity, or other socially relevant groupings	Not applicable
Population characteristics	Not applicable
Recruitment	Not applicable
Ethics oversight	Not applicable

Note that full information on the approval of the study protocol must also be provided in the manuscript.

Field-specific reporting

Please select the one below that is the best fit for your research. If you are not sure, read the appropriate sections before making your selection.

☒ Life sciences ☐ Behavioural & social sciences ☐ Ecological, evolutionary & environmental sciences

For a reference copy of the document with all sections, see nature.com/documents/nr-reporting-summary-flat.pdf

Life sciences study design

All studies must disclose on these points even when the disclosure is negative.

Sample size	Analysis of TF binding effects on UV damage formation and repair was done for 225 TF motif clusters curated from Vierstra et al., Nature 2020. For structural analysis of AlphaFold 3-predicted TF-DNA complexes, the top 20 unique TF binding site sequences (based on motif quality score) were used to generate unbound and TF-bound versions of binding site structures per dipyrimidine position of interest. In total, 388 TF-DNA complexes were generated, covering 11 positions across 5 TFs. For ATAC-seq, 4 biological replicates were used for IDR analysis peak calling.
Data exclusions	Vierstra et al. originally published 286 TF motif clusters. Four clusters were removed due to naming redundancy (of which the cluster that best represented human protein orthologs was kept), three clusters were removed due to low motif model specificity and two clusters were removed because they lacked a human ortholog TF in the motif model. Finally, as our limit of detection, we only analyzed TF motif clusters that had at least one position in their binding site with at least 30 aggregated UV lesions formed immediately after UV treatment. After this filtering, our analysis consisted of 225 motif clusters. In addition to intersecting the genome-wide TF motif cluster binding site calls with chromatin accessibility data, for each TF cluster, we excluded the bottom 50% of TF binding sites based on motif quality score (reported by Vierstra et al.) to enrich for actively TF binding sites. Binding sites for KLF/SP/1 and CTCF clusters were further limited to the top 10,000 of binding sites due to the large number of genome-wide binding sites called for these clusters. For all AlphaFold 3 generated structures used in our structural analysis, 3 base pairs at either DNA chain termini were excluded from analysis due to having consistently lower confidence

predictions observed at these positions for all predicted DNA. Only AlphaFold 3 generated structures where all DNA residues had a mean pLDDT score in the "confident" or above range (pLDDT >= 70) were used in analysis.

Replication	Replication through orthogonal validation of results. Our in vivo CPD formation frequency measurements across tetranucleotide sequences correlated strongly (Pearson r=0.88) with previous in vitro experiments of CPD formation in free DNA published by Lu et al. (https://doi.org/10.1093/nar/gkab214). To test the reproducibility of the results from our analytical Poisson model of CPD formation, we analyzed CPD formation in TF binding sites using an orthogonal simulation method which yielded very similar results (Pearson R=0.84). Both replication exercises were successful.
Randomization	A case-control study that would necessitate randomization was not included in this manuscript. During bootstrap and permutation analyses, random sampling was done using pandas.DataFrame.sample implementation in conjunction with a pseudo-random seed generated using numpy.random.seed.
Blinding	Blinding was not relevant to our study as the majority of our analyses were on previously published data, and we conducted no additional experiments to comparing experimental conditions that would necessitate blinding. No animals or human research participants were involved in this study.

Reporting for specific materials, systems and methods

We require information from authors about some types of materials, experimental systems and methods used in many studies. Here, indicate whether each material, system or method listed is relevant to your study. If you are not sure if a list item applies to your research, read the appropriate section before selecting a response.

Materials & experimental systems

n/a	Involved in the study
☒	☐ Antibodies
☐	☒ Eukaryotic cell lines
☒	☐ Palaeontology and archaeology
☒	☐ Animals and other organisms
☒	☐ Clinical data
☒	☐ Dual use research of concern
☒	☐ Plants

Methods

n/a	Involved in the study
☒	☐ ChIP-seq
☒	☐ Flow cytometry
☒	☐ MRI-based neuroimaging

Eukaryotic cell lines

Policy information about cell lines and Sex and Gender in Research

Cell line source(s)	Human skin fibroblast cell lines were used for UVDE-seq (NHF1), CPD-seq v2.0 (CSB/CS1AN^WT), and ATAC-seq (CSB/CS1AN^WT) experiments.
Authentication	Authenticated with genome sequencing
Mycoplasma contamination	Cells were not tested for Mycoplasma contamination
Commonly misidentified lines (See ICLAC register)	None

Plants

Seed stocks	Not applicable
Novel plant genotypes	Not applicable
Authentication	Not applicable
